# *Ortho*-aryl substituted DPEphos ligands: rhodium complexes featuring C–H anagostic interactions and B–H agostic bonds†

**DOI:** 10.1039/d1sc01430g

**Published:** 2021-05-25

**Authors:** James J. Race, Arron L. Burnage, Timothy M. Boyd, Alex Heyam, Antonio J. Martínez-Martínez, Stuart A. Macgregor, Andrew S. Weller

**Affiliations:** Department of Chemistry, University of York Heslington York YO10 5DD UK andrew.weller@york.ac.uk; Chemistry Research Laboratories, University of Oxford Oxford OX1 3TA UK; Institute of Chemical Sciences, Heriot Watt University Edinburgh EH14 4AS UK S.A.Macgregor@hw.ac.uk

## Abstract

The synthesis of new Schrock–Osborn Rh(i) pre-catalysts with *ortho*-substituted DPEphos ligands, [Rh(DPEphos-R)(NBD)][BAr^F^_4_] [R = Me, OMe, ^i^Pr; Ar^F^ = 3,5-(CF_3_)_2_C_6_H_3_], is described. Along with the previously reported R = H variant, variable temperature ^1^H NMR spectroscopic and single-crystal X-ray diffraction studies show that these all have axial (C–H)⋯Rh anagostic interactions relative to the d^8^ pseudo square planar metal centres, that also result in corresponding downfield chemical shifts. Analysis by NBO, QTAIM and NCI methods shows these to be only very weak C–H⋯Rh bonding interactions, the magnitudes of which do not correlate with the observed chemical shifts. Instead, as informed by Scherer's approach, it is the topological positioning of the C–H bond with regard to the metal centre that is important. For [Rh(DPEphos–^i^Pr)(NBD)][BAr^F^_4_] addition of H_2_ results in a Rh(iii) ^i^Pr–C–H activated product, [Rh(κ^3^,σ-P,O,P-DPEphos-^i^Pr′)(H)][BAr^F^_4_]. This undergoes H/D exchange with D_2_ at the ^i^Pr groups, reacts with CO or NBD to return Rh(i) products, and reaction with H_3_B·NMe_3_/*tert*-butylethene results in a dehydrogenative borylation to form a complex that shows both a non-classical B–H⋯Rh 3c-2e agostic bond and a C–H⋯Rh anagostic interaction at the same metal centre.

## Abstract 2

Rh(i) complexes of *ortho*-substituted DPEphos-R (R = H, Me, OMe, ^i^Pr) ligands show anagostic interactions; for R =^i^Pr C–H activation/dehydrogenative borylation forms a product exhibiting both B–H/Rh 3c-2e agostic and C–H/Rh anagostic motifs.

## Graphical abstract

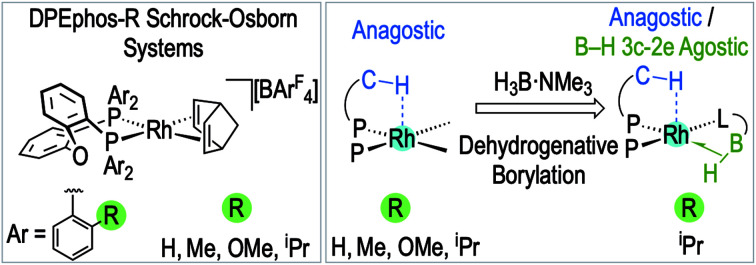

## Introduction

**Fig. 1:**
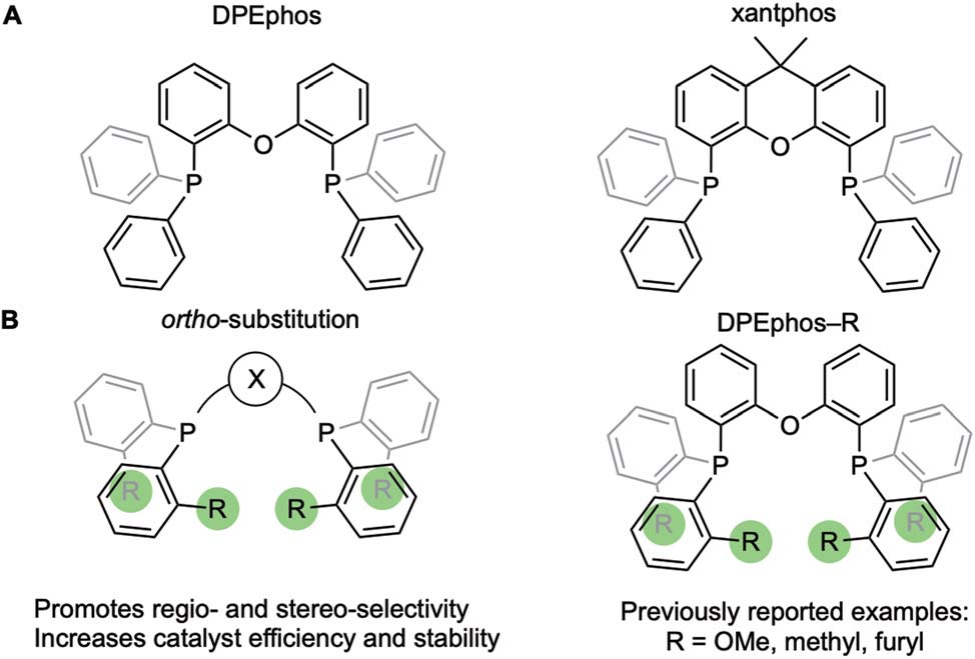
(A) Xantphos and DPEphos ligands. (B) *Ortho*-aryl substitution.

Diphosphine chelates that contain an ether linkage in their backbone, such as DPEphos and xantphos, are an important and popular class of ligand that are used in synthesis and catalysis (Fig. 1A). Initially developed as wide bite-angle, κ^2^-P,P-*cis*-coordinating, ligands for Rh-based hydroformylation catalysis,^1,2^ such ligands also have the ability to act in κ^3^-P,O,P binding modes often leading to hemilabile^3^ behaviour through reversible coordination of the ether linkage in response to changes in the metal coordination sphere or oxidation state. DPEphos is now widely used in a variety of catalytic settings,^4–7^ and the vast majority of applications make use of the commercially available phenyl phosphine derivative. Modification of aryl phosphine ligands, more generally, by introducing steric bulk using *ortho*-substitution has been shown to promote enantioselectivity;^8^ regioselectivity;^9^ overall efficiency and catalyst stability;^10–13^ as well as aryl-group restricted rotation.^14^ Despite these potential advantages, *ortho*-substituted variants of DPEphos (or xantphos) are rare, Fig. 1B, and their use limited to a handful of examples.^11,15–19^

**Fig. 2:**
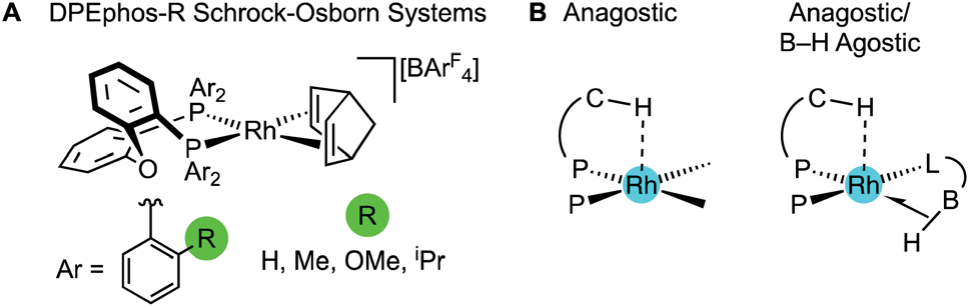
(A) [Rh(DPEphos-R)(NBD)][BAr^F^_4_] systems, and (B) schematic examples of the C–H anagostic interactions and 3c-2e B–H agostic bonds, reported in this contribution.

The cationic Schrock–Osborn [Rh(chelating-phosphine)]^+^ system is widely used in catalysis and synthesis,^20,21^ and the active species are often accessed *via* hydrogenation of a suitable diene precursor, such as [Rh(chelating-phosphine)(NBD)][anion] (NBD = norbornadiene), in a coordinating solvent such as acetone. We have particular interest in such systems with the DPEphos ligand, with regard to their use as pre-catalysts for amine-borane dehydropolymerisation,^22,23^ alkene and alkyne hydroacylation,^24–26^ and alkyne carbothiolation,^27^ amongst other applications. We now report the synthesis of new Schrock–Osborn systems with *ortho*-substituted DPEphos ligands, including a new ^i^Pr-substituted ligand (Fig. 2). A detailed structural, variable-temperature spectroscopic, and computational study reveals these to show well-defined examples of anagostic C–H⋯Rh interactions,^28,29^ even for the previously-reported^24^ parent DPEphos complex; while a reactivity study demonstrates intramolecular C–H activation can occur after hydrogenation of the NBD ligand, that is dependent on the identity of the R-group. Reaction of such a cyclometallated complex with H_3_B·NMe_3_ leads to a dehydrogenative borylation and a complex that features both non-classical B–H 3c-2e agostic^28^ and anagostic C–H structural and spectroscopic features, Fig. 2B. This serves to highlight the key differences between anagostic and agostic motifs of E–H bonds with d^8^-metal centres in a single complex.

In describing the anagostic interactions in these systems we borrow from the analysis of Scherer^30^ who showed that axial positioning of a C–H bond at a square-planar d^8^ metal centre orientates it over a region of charge concentration. When the complex is then placed in a magnetic field (*i.e.*, the NMR experiment) induced current density at the metal results in magnetic field effects that cause the signature downfield chemical shift of the anagostic proton. In our analysis we find that descriptors that define the bonding between the Rh centres and C–H bonds show no correlation with either the observed or computed chemical shifts, supporting Scherer's topological, induced current, description for anagostic interactions.

## Results and discussion

### Synthesis and solid-state structures of the NBD complexes

**Fig. 3:**
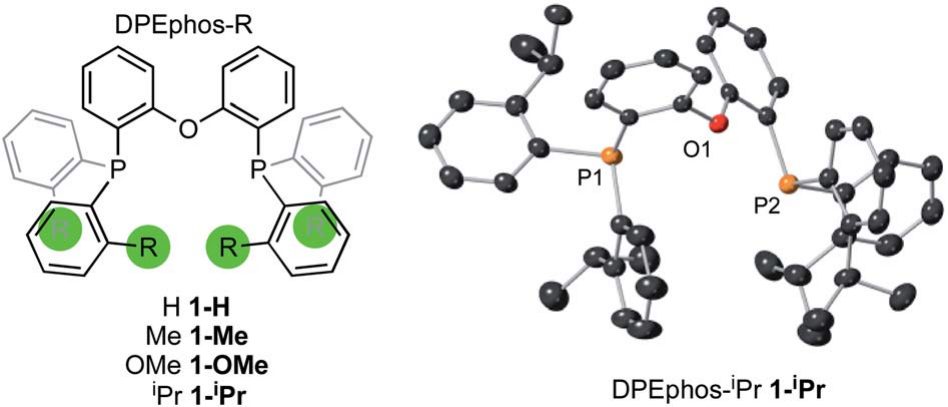
DPEphos-R ligands used in this study. Crystallographically determined structure of **1-iPr**. Ellipsoids shown at the 50% probability level. Hydrogen atoms omitted for clarity. See ESI† for full details.

The *ortho*-substituted DPEphos-R ligands used in this study are shown in Fig. 3: R = H, **1-H**; Me, **1-Me**; OMe, **1-OMe**; and ^i^Pr, **1-iPr**. Ligands **1-H** and **1-Me** are commercially available, **1-OMe** was prepared using the reported procedure.^17^ DPEphos-^i^Pr, **1-iPr**, is a new ligand and was prepared as an analytically pure white solid from reaction of the corresponding dichlorophosphine with *ortho*-isopropyl phenyl lithium (ESI†). The solid-state structure is shown in Fig. 3. In the room temperature ^31^P{^1^H} NMR spectrum a single ^31^P environment is observed at *δ* −37.6.

Interestingly, the room temperature ^1^H NMR spectrum is rather simple with only a single (integral 24 H) environment observed for the ^i^Pr-methyl groups – despite their diastereotopic nature in the solid-state structure. This suggests inversion at P is a low energy process for free **1-iPr**,^31^ which has been shown to be the case for other bulky ^i^Pr-substituted tris-aryl phosphines.^32^

**Scheme 1:**
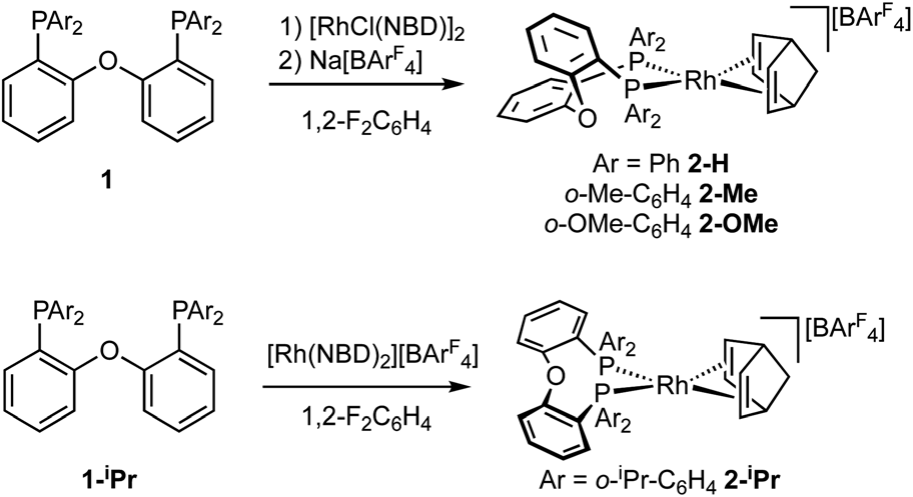
Synthesis of the new Rh-complexes.

**Fig. 4:**
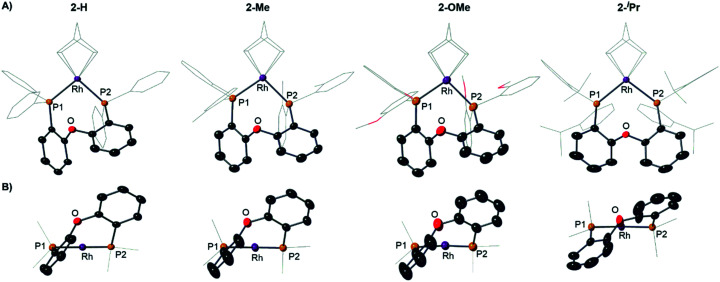
(A) Solid-state structures of the cations in **2-H**, **2-Me**, **2-OMe** and **2-iPr** as determined by single-crystal X-ray diffraction. Displacement ellipsoids are shown at the 50% probability level. Hydrogen atoms and [BAr^F^_4_]^−^ anions not shown. Selected DPEphos-R and NBD groups shown as wireframe. (B) End-on view highlighting the relative orientation of the DPEphos backbones. Bond lengths and angles are given in the ESI (Table S2).†

The target, Schrock–Osborn, [Rh(DPEphos-R)(NBD)][BAr^F^_4_] complexes [Ar^F^ = 3,5-(CF_3_)_2_C_6_H_3_] were prepared by addition of the DPEphos-R ligands to the appropriate Rh-precursor. [Rh(DPEphos-H)(NBD)][BAr^F^_4_], **2-H**, has already been reported to be formed from addition of **1-H** to [Rh(NBD)Cl]_2_, using Na[BAr^F^_4_] to extract the halide (Scheme 1).^24^ A slightly refined method, using 1,2-F_2_C_6_H_4_ as a solvent, was used to make [Rh(DPEphos-R)(NBD)][BAr^F^_4_], R = H, **2-H**; Me, **2-Me**; and OMe, **2-OMe**. For the bulkier ligand, **1-iPr**, [Rh(NBD)_2_][BAr^F^_4_] was used to make **2-iPr**. The new complexes were isolated in moderate to good yield (65 to 85%), as crystalline solids. Fig. 4 shows the solid-state structures of the cations in these new complexes as determined by single-crystal X-ray diffraction. While **2-H** is known,^24^ the solid-state structure had not been reported, and so is included here. All the cations have pseudo square planar Rh(i) centres, with the NBD ligands binding η^2^η^2^, and *cis*-κ^2^-P,P DPEphos-R ligands. Bond lengths and angles are generally unremarkable (ESI†). The closest Rh⋯O distance in **2-OMe** is 3.081(3) Å from an axially-orientated methoxyl group – which is clearly non-bonding.

Notable differences, however, come from the relative orientation of the DPEphos-R diphenylether backbone, Fig. 4B. For **2-H**, **2-Me** and **2-OMe** this lies above the P–Rh–P plane sitting in an asymmetric envelope-like conformation.^33^ If retained in solution this would give the cation *C*_1_ symmetry (*i.e.* none). The ^i^Pr groups in **2-iPr** force a, non-crystallographic, *C*_2_-axis. Reflecting the increase in steric bulk, the Rh–P distances are ∼0.1 Å longer and the P–Rh–P bite angle ∼ 3° wider in **2-iPr** compared with the other complexes (Table S2†). In all cases the DPEphos ether oxygen atom sits distant from the Rh-centre [3.498(8)–3.5545(18) Å]. For all, there are aryl or methyl C–H bonds in the *ortho*-aryl groups that are axially positioned above the Rh-square plane, *i.e.* potential anagostic interactions. These are discussed in detail after the solution NMR spectroscopic data have been presented that signal this orientation.

### Variable temperature solution NMR spectroscopy and the identification of anagostic interactions in solution and solid-state

**Fig. 5:**
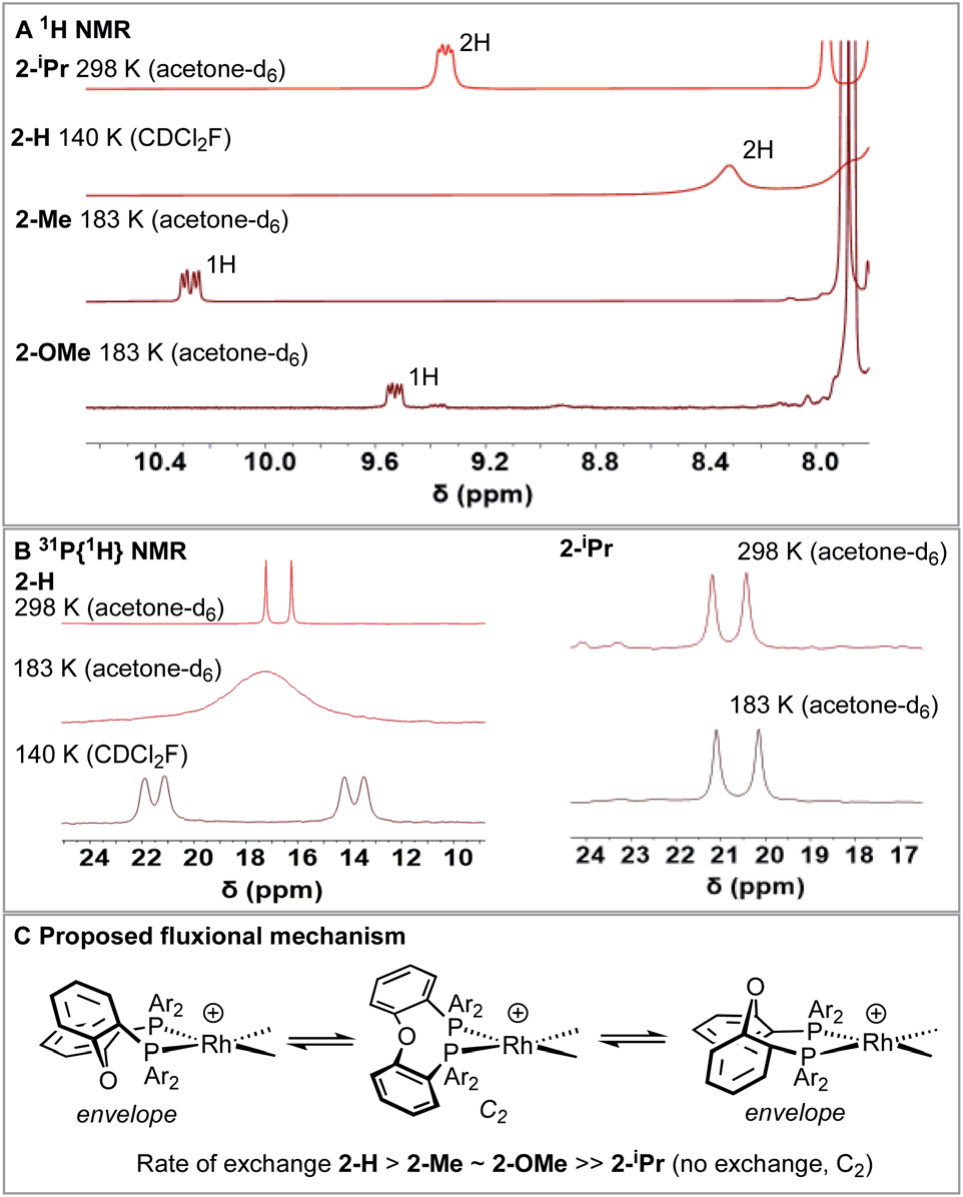
(A) Low-field (*δ* 7.8–10.5) region of the ^1^H NMR spectra for the [Rh(DPEphos-R)(NBD)][BAr^F^_4_] complexes showing the shifted signals (temperature and solvent as noted) (B) ^31^P{^1^H}NMR spectra for **2-H** and **2-iPr** at various temperatures. (C) Proposed fluxional process.

Room temperature NMR spectra of the Rh–NBD complexes indicate fluxional behaviour in solution that is dependent on the identity of the phosphine ancillary group. For **2-H**^24^ a very simple, sharp, set of signals is observed for the room temperature ^1^H NMR spectrum (*i.e.*, a single NBD alkene environment), along with a single environment in the ^31^P{^1^H} NMR spectrum. Together these indicate time averaged *C*_2v_ symmetry in solution. For **2-Me** broad signals are observed in both the ^1^H NMR and ^31^P{^1^H} NMR spectra, with the latter showing two species: one with a single ^31^P environment and one with inequivalent environments. For **2-OMe** the situation is similar, except that only one – very broad – environment is observed in the ^31^P{^1^H} NMR spectrum. These data, in comparison with the solid-state structures, suggest fluxional processes are operative in solution that are fast for **2-H**, but slower for **2-Me** and **2-OMe** and also involve observable equilibrium populations of different conformers. For bulky **2-iPr** the NMR spectra are again sharp, but now indicate *C*_2_, rather than *C*_2v_, symmetry for the NBD (four signals) and DPEphos-^i^Pr (two methine, four CH_3_ and one ^31^P environment) ligands *via*^1^H and ^31^P{^1^H} NMR spectroscopy. In the low-field region of the ^1^H NMR spectrum of **2-iPr** a distinct, relative integral 2H, signal is observed at *δ* 9.34 that shows coupling to P and H [*J*(PH) = 17, *J*(HH) = 7 Hz], Fig. 5A. There is no evidence for Rh–H coupling.

**Fig. 6:**
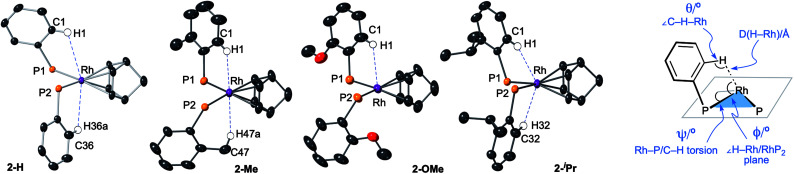
Views of the NBD complexes highlighting the close C–H⋯Rh, anagostic, interactions with selected structural markers. Diphenyl ether linkages on the DPEphos-R ligands are not shown. Hydrogen atoms are placed in calculated positions.

While such downfield shifted signals are not observed in the room temperature ^1^H NMR spectra of the other complexes, progressive cooling to much lower temperatures reveals similarly shifted peaks and corresponding changes in the ^31^P NMR spectra. For **2–H** cooling to 183 K (acetone-d_6_) results in very broad signals in the ^1^H NMR spectrum, suggesting the low temperature limit had not been reached. By using CDCl_2_F^34^ as a solvent a ^1^H NMR spectrum could be obtained at 140 K in which a low-field shifted, albeit broad, signal (2H) is observed at *δ* 8.32. For **2-Me** and **2-OMe** similar behaviour is observed on cooling but now 243 K and 203 K, respectively, are sufficient to reveal downfield-shifted aromatic resonances.^35^ However, these integrate to only 1H each, at *δ* 10.27 and *δ* 9.53 respectively (in acetone-d_6_, 183 K). **2-Me** also shows a downfield shifted methyl resonance at *δ* 3.68 (3H, 183 K). For **2-Me** and **2-OMe** four different NBD alkene environments are observed in the low temperature ^1^H NMR spectra, along with two mutually coupled signals in the corresponding ^31^P{^1^H} NMR spectra [*e.g. J*(PP) = 28 Hz **2-Me**] that also couple to ^103^Rh. For **2-H** these signals are broad even at 140 K (fhwm = 80 Hz) and the ^31^P–^31^P coupling is not resolved, Fig. 5B. These data point to fluxional processes that are arrested, or considerably slowed, at low temperature to give structures that are similar to those determined in the solid-state, *i.e.* an envelope-like conformation of the DPEphos-R ligand. On increasing the temperature, conversion between enantiomeric *C*_1_ forms *via* a *C*_2_ intermediate is proposed, Fig. 5C. This has been modelled for **2-Me** using line-shape analysis (see ESI†). Related ring-flipping processes in POP-type ligands have been reported previously.^36,37^ For **2-iPr** there is no change on cooling (Fig. 5B), the ∼*C*_2_-symmetric solid-state structure is retained in solution at room temperature. It is thus not fluxional. These observations are consistent with relative steric bulk of the *o*-substituents: **1-H** < **1-Me** ∼ **1-OMe** ≪ **1-iPr**. Downfield chemical shifts in the ^1^H NMR spectrum can be diagnostic of anagostic C–H interactions, which are located above a region of charge concentration at a d^8^ metal centre, *i.e.* an occupied d_*z*^2^_ orbital.^30,38–40^ These are distinct from agostic,^28^ 3c-2e, bonds that are characterised by donation from a C–H bond into an unoccupied metal orbital and upfield chemical shifts in the ^1^H NMR spectrum. The fluxional processes operating at room temperature mean these characteristic signals are only resolved on cooling, apart from for **2-iPr** in which the static structure makes them persistent. We next turn to inspecting the solid-state structures of the NBD adducts more closely to identify such anagostic interactions: Fig. 6 and Table 1.

**Table 1:** Structural and spectroscopic data that describe the C–H⋯Rh interactions in the DPEphos-R complexes

	**2-H**	**2-Me**	**2-OMe**	**2-iPr**
*Θ* (°)^a^	114.7, 122.6	129.8, (144.2)^c^	121.6	132.7, 135.6
*Ψ* (°)^a^	42.0, 1.7	1.3^c^	−6.2	−1.4, −8.2
*Φ* (°)^a^	63.1, 58.0	64.3, (69.3)^c^	63.9	64.3, 64.3
Rh⋯H1 (Å)	2.92, 2.97	2.57, (2.63)^c^	2.88	2.58, 2.47
*δ*(H) (ppm)	8.32	9.97, (3.56)^c^	9.19	9.14
Δ*δ*(H) (ppm)^b^	+0.99 to 1.11^d^	+2.82, (+1.3)^c^	+2.34	+1.85
*J*(PH) (Hz)	Broad	17	17	18
*J*(HH) (Hz)	Broad	8	7	8

a See Fig. 6 for definitions.

b Difference in chemical shift of H1 (500 MHz, CD_2_Cl_2_, 203 K) compared with free ligand (CD_2_Cl_2,_ 295 K).

c Numbers in parenthesis associated with methyl groups.

d The *ortho* phenyl protons in DPEphos-H could not be unambiguously identified.

All four complexes show relatively close C–H⋯Rh approaches from an *ortho* C(aryl)–H group in the phenyl phosphine (H atoms in calculated positions, see Table 2 for computational analysis). For **2-H** there are two, albeit long (∼2.9 Å); for **2-iPr** there are also two, but these are considerably shorter (∼2.5 Å); while **2-OMe** has a single close C(aryl)–H⋯Rh distance (∼2.9 Å). **2-Me** shows two different types: C(aryl)–H⋯Rh (2.57 Å), and C(Me)–H⋯Rh (2.63 Å). The phenyl rings associated with these C(aryl)–H⋯Rh contacts generally align with the associated Rh–P vector (C–H/Rh–P torsion angles, *Ψ*, 8.2 to 1.3°) and the C–H⋯Rh angle (θ) is rather open (121.6–144.2°). Although **2-H** has one phenyl ring twisted away from this (*Ψ* = 42.0, *θ* = 114.7°), the Rh⋯H distance is similar. The number of these close C–H⋯Rh distances correlates well with relative integrals of the downfield shifted signals observed in the ^1^H NMR spectra: **2-H**, 2H; **2-Me**, 1H (aryl), 3H (methyl); **2-OMe**, 1H; and **2-iPr**, 2H. As there is no crystallographically imposed symmetry in the solid-state we assume any equivalent environments observed in solution arise from very low energy fluxional processes. The changes in chemical shifts of these C–H protons due to the presence of the Rh(i) centre have been experimentally determined by comparison with the free ligands, as aided by ^1^H/^1^H COSY, HMBC and HSQC experiments. While all shift downfield, the variation observed shows no strong correlation with any of the structural descriptors discussed above, as detailed in Table 1. However, in a more general sense, for all the complexes the angle formed between the RhH vector and the RhP_2_ plane (*ø*) shows the C–H proton is orientated towards the apical position (which at the limit *ø* = 90°). Thus, following Scherer's analysis,^30^ the positioning of the C–H bond over a region of charge concentration (occupied d orbitals, *ø* approaching 90°) induces the downfield chemical shift in the NMR spectrum that is diagnostic of an anagostic interaction. In contrast, orientation of a C–H bond toward a charge depleted region (a vacant orbital in the metal coordination plane, *ø* approaching 0°) results in upfield-shifted signals that are characteristic of agostic, 3c-2e, bonding. Such demarcations are not always clear-cut, however, as axial sites can also display Lewis-acidic character.^29,41^

**Table 2:** Computed metrics for the C–H⋯Rh interactions in the DPEphos-R complexes^a^

Cation	Bond path	Distance/Å	QTAIM (au)			NBO donor–acceptor interactions (kcal mol^−1^)		NMR/ppm	
			*ρ*(*r*)	∇^2^*ρ*(*r*)	*q*_H_	σ_C–H_ → Rh^b^	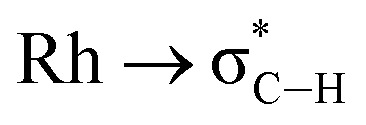 c	*δ*(H)_calc_^d^	*δ*(H)_exp_
**[2-H]+**	Rh⋯H1	2.83	0.012	+0.036	0.031	0.57	1.33	+9.5	+8.32
	Rh⋯H36a	2.87	0.011	+0.030	0.028	0.52	1.22	+9.1	
**[2-Me]+**	Rh⋯H1	2.45	0.022	+0.053	0.026	0.69	4.38	+10.6	+9.97
	Rh⋯H47a	2.51	0.020	+0.045	0.027	0.49	4.29	+6.0 (+3.9^e^)	+3.56
**[2-OMe]+**	Rh⋯H1	2.79	0.013	+0.035	0.047	0.33	1.91	+9.6	+9.19
**[2-iPr]+**	Rh⋯H1	2.33	0.026	+0.059	0.024	2.08	8.98	+9.9	+9.14
	Rh⋯H32	2.45	0.021	+0.050	0.027	1.71	6.70	+9.8	

a QTAIM and NBO data are based on the experimental crystal structures; computed chemical shifts are based on the lowest energy conformations.^45^

b Sum of donation into the two 
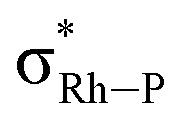
 NBOs.

c Sum of donation from the Rh lone pairs and σ_Rh–P_ bonding NBOs.

d Data are weighted averages taking into account all low energy conformations.

e Average of all three Me hydrogens. See ESI for full details.

**Fig. 7:**
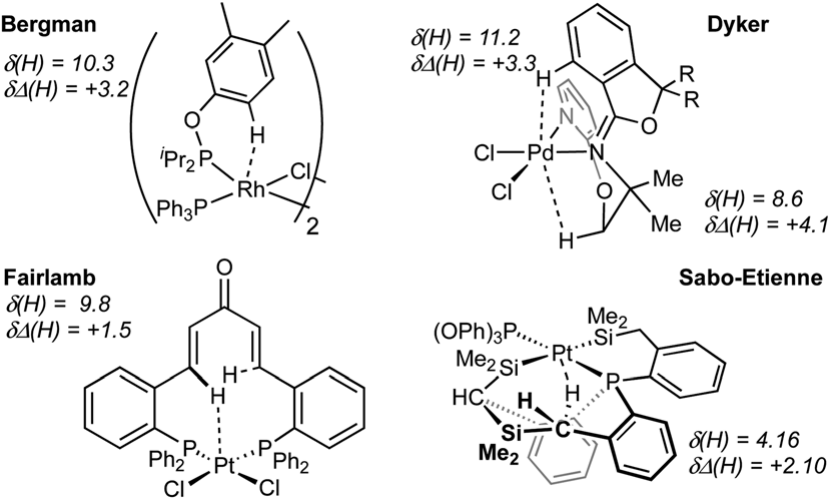
Examples of previously reported anagostic C–H⋯M interactions.

While with hindsight it is not surprising that the most sterically bulky ligand, DPEphos–^i^Pr, enforces an anagostic interaction at room temperature, the presence of both aryl and, rarer,^42,43^ alkyl anagostic interactions in **2-Me** is perhaps more notable. What was unanticipated is that in the parent DPEphos–H complex such interactions are also present – albeit only observed at very low temperature in solution. Similar properties (C–H⋯M, 2.23–3.01 Å, low-field chemical shifts and apical approaches of C–H groups to d^8^ metal centres), have been discussed by others, including: Bergman,^44^ Dyker,^42^ Fairlamb,^45^ and Sabo-Etienne,^38^Fig. 7.

So, while the presence of anagostic C–H⋯Rh(i) interactions has been demonstrated here experimentally by both structural and spectroscopic studies, the correlation between the observed chemical shifts and measured structural descriptors is less obvious. We thus turned to a computational analysis to examine the nature of these anagostic C–H⋯Rh(i) interactions more closely.

### Computational studies: structures, bonding and chemical shifts

**Fig. 8:**
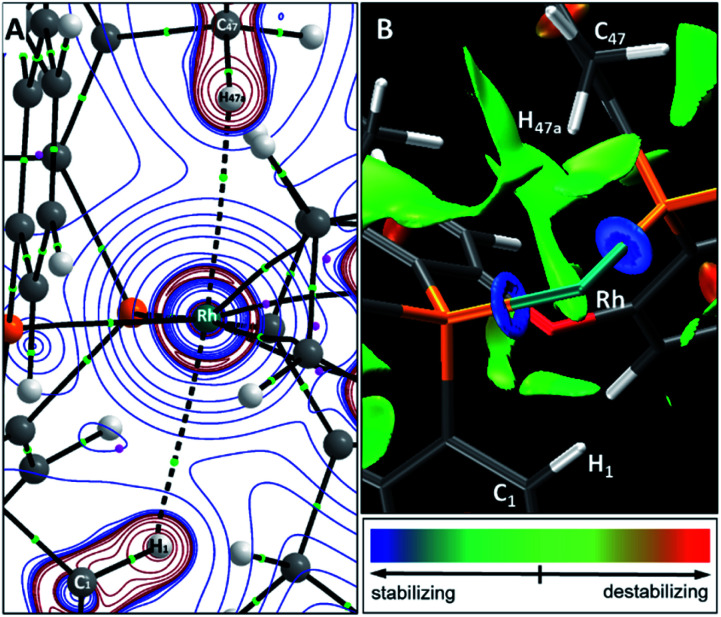
(A) Molecular graph of the **[2-Me]+** cation showing the contour plot of the Laplacian in the H_47a_RhH_1_ plane. Bond critical points and ring critical points are shown as green and pink spheres respectively; blue contours show areas of charge depletion, red contours charge accumulation; (B) non-covalent interaction plot highlighting weak stabilising Rh⋯H_1_ and Rh⋯H_47a_ interactions; the NBD ligand is removed for clarity and the isosurface is generated for *σ* = 0.3 au and −0.07 < *ρ* < 0.07 au. Key shows isosurface colouring.

Computed metrics for the Rh⋯H–C moieties in the isolated cations of all four DPEphos-R complexes are provided in Table 2. Geometries for these analyses are based on the experimental structures with the heavy atoms fixed at their observed positions and the H atoms optimised. The calculated Rh⋯H distances are therefore *ca.* 0.1 Å shorter (and the C–H bonds *ca.* 0.15 Å longer) than those determined experimentally. Fig. 8 displays the molecular graph, the topology of the Laplacian and a non-covalent interaction (NCI) plot for the cationic portion of **2-Me**, **[2-Me]+**, where we have chosen to showcase the system featuring both aryl- and alkyl-C–H⋯Rh anagostic interactions. The presented data are representative of all four cations and equivalent figures for the remaining systems are provided in the ESI.† The bond critical point (BCP) metrics indicate the presence of weak Rh⋯H–C interactions with low BCP electron densities, *ρ*(*r*), small positive values for the Laplacian and small, positive charges on the anagostic H atoms. In **[2-Me]+** the Rh⋯H_47a_ (alkyl) interaction is slightly weaker than the Rh⋯H_1_ (aryl) interaction, although this likely reflects the longer Rh⋯H_47a_ distance rather than any intrinsic difference. Plots of *ρ*(*r*) and ∇^2^*ρ*(*r*) against the computed RhH distances provide excellent correlations (Fig. S42 and S43†) and the strongest Rh⋯H–C interactions are seen in **[2-iPr]+**. This is mirrored in the NBO 2nd order perturbation analyses that show the major component, 
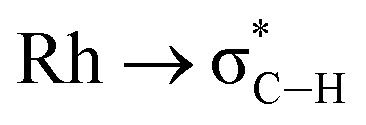
 donation, to increase upon shortening the Rh⋯H distance. σ_C–H_ → Rh donation shows a similar trend but this is minimal, even in **[2-iPr]+**. This weak Rh⋯H–C interaction therefore shares some characteristics of a H-bond^46,47^ and this is also evident in the NCI plot of **[2-Me]+** where light turquoise (*i.e.* weakly stabilising) regions are seen along the Rh⋯H_1_ and Rh⋯H_47a_ vectors.

The Laplacian plot around the Rh atom in **[2-Me]+** indicates that both the Rh⋯H_1_ and Rh⋯H_47a_ bond paths pass through regions of axial charge concentration. Thus both C–H bonds are oriented towards areas of charge accumulation at Rh, consistent with the downfield ^1^H anagostic chemical shift.^30^ Computed ^1^H NMR chemical shifts reproduce these downfield shifts for all four cations. In this case the calculations were performed on the fully optimised structures to model behaviour in solution. In general, the computed chemical shifts lie further downfield than the experimental values. The largest discrepancy is for **[2-H]+** and this may reflect that the low temperature limit had not been achieved experimentally. In addition, conformational searching revealed additional low energy structures that also contribute to the final observed chemical shift.^48^ For **[2-Me]+** the static structure in the calculations reveals the large downfield shift associated with the Me proton H_47a_ (*δ*_calc_ = +6.0 ppm) while the average of all three Me protons is 3.9 ppm, in good agreement with experiment (*δ* 3.56) where the methyl group will be freely rotating leading to a weighted-average chemical shift.

Interestingly, although there is a clear relationship between the Rh⋯H–C distance and the computed bonding metrics, no such correlation is seen with the computed chemical shifts of the anagostic hydrogens (Table 2). Thus, the nature of the Rh⋯H–C interaction does not relate to the extent of the downfield chemical shift, suggesting the orbitals involved are not responsible for the chemical shift. Instead the situation is more consistent with Scherer's observations^30^ that it is the spatial positioning of the anagostic H above the d^8^ square-planar metal coordination plane (*i.e. ø*) together with the complex interplay of induced current densities that are responsible for the precise chemical shift observed. Thus while the computation of a weak M⋯H bond path and weak 
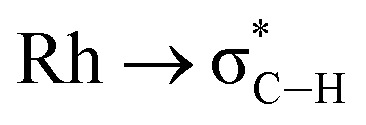
 donation are usually features that are associated with an anagostic interaction,^40^ they are not in themselves responsible for the signature downfield chemical shifts observed in NMR spectra that signal the positioning of the C–H bond relative to the metal centre.

### Reactivity: hydrogenation of NBD, reversible C–H activation, and a complex with both anagostic and B–H agostic motifs

The Schrock–Osborn [Rh(DPEphos-R)(NBD)]^+^ complexes are precatalysts for a variety of important transformations.^20^ Activation is often by hydrogenation *in situ* in a coordinating solvent, for example acetone to form [Rh(DPEphos-R)(acetone)_2_]^+^ (**3-R**) and free norbornane (NBA).^9,24,49^ [Rh(DPEphos-H)(acetone)_2_][BAr^F^_4_]^25^ has been reported using this method, and we now extend this methodology to the complexes **2-Me**, **2-OMe** and **2-iPr**. The product of these reactions is dependent on the R-substituent, with more electron donating/bulkier substituents resulting in Rh(iii) hydride products.^50^

**Scheme 2:**
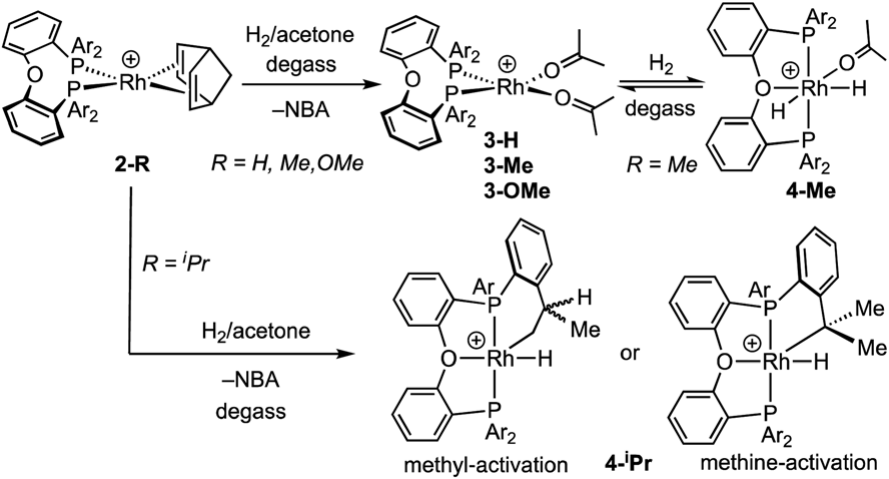
Hydrogenation of NBD adducts **2-R**. [BAr^F^_4_]^−^ anions not shown.

Addition of H_2_ to yellow acetone-d_6_ solutions of **2-Me** or **2-OMe**, followed by degassing, results in the hydrogenation of bound NBD and the *in situ* formation of the red acetone adducts^49^**3-Me** and **3-OMe** (Scheme 2). These adducts could not be isolated and presented broad signals at room temperature in their ^1^H and ^31^P NMR spectra. Free NBA was observed to be formed by ^1^H NMR spectroscopy. For R = Me, if the solution is not degassed post H_2_ addition, the yellow Rh(iii) dihydride complex, [Rh(DPEphos-Me)(H)_2_(acetone)][BAr^F^_4_], **4-Me**, is formed quantitatively. Degassing results in loss of H_2_ and the formation of red **3-Me**. Complex **4-Me** is characterised at 298 K by the observation in the ^1^H NMR spectrum of a broad, relative integral 2H, hydride resonance at *δ* −19.5 in the region characteristic of hydride ligands, and a broad signal in the ^31^P{^1^H} NMR spectrum at *δ* 26. Cooling to 183 K reveals sharper signals, and thus that a fluxional process is occurring, likely reversible dissociation of acetone.^51,52^ A major and a minor species are observed (5 : 1 ratio) at low temperature, both with inequivalent hydrides [*ca. δ* −18 and −20] that integrate in total to 2H and show coupling to Rh, P and the other hydride [dddd]. In the ^31^P{^1^H} NMR spectrum signals are observed that show large *J*(PP) coupling [*ca.* 340 Hz] and small *J*(RhP) [*ca.* 117 Hz] – identifying them as being in a *trans* arrangement on a Rh(iii) centre.^53^ These data, alongside selective decoupling experiments (ESI†), allow a structure to be assigned for **4-Me** as shown in Scheme 2, that is similar to [Rh(κ^3^-P,O,P-xantphos)(H)_2_(acetone)][BAr^F^_4_].^51^ The two different species observed at low temperature are assigned to conformers arising from different orientations of the *ortho*-Me substituted phenyl groups that undergo restricted P–C rotation.^10,14^

For the DPEphos-^i^Pr ligand the product of hydrogenation in acetone is different, and a Rh(iii)-hydride ^i^Pr-cyclometallated product is formed, [Rh(κ^3^-P,O,P-DPEphos-^i^Pr′)(H)][BAr^F^_4_] **4-iPr** [DPEphos-^i^Pr′ = (*o*-^i^Pr-C_6_H_4_)_2_P(C_6_H_4_)O(C_6_H_4_)P(*o*-^i^Pr-C_6_H_4_)(*o*-(CH_2_CH_3_CH)C_6_H_4_)]. **4-iPr** is fluxional in solution, and is unchanged when free H_2_ is removed under vacuum. At room temperature in acetone-d_6_ solution the formation of **4-iPr** is signalled by a, integral 1H, environment observed at *δ* −19.81 [dt, *J*(RhH) = 29, *J*(PH) 15 Hz], an alkyl region that shows a complex set of overlapping resonances (further complicated by the presence of free NBA), and a very broad ^31^P{^1^H} NMR spectrum [*δ* 20.8]. Warming to 338 K sharpens the ^31^P NMR spectrum, so a broad apparent doublet is observed at *δ* 21.7;^54^ while the ^1^H NMR spectrum at this temperature retains a sharp multiplet hydride signal. There is some decomposition on warming. Progressive cooling moves though a coalescence regime, ∼243 K, so that at 183 K a sharp ^31^P{^1^H} NMR spectrum is observed that shows three major sets of inequivalent phosphine environments, between *δ* 4 and *δ* 41, all with *trans* P–P coupling [*J*(PP) ∼360 Hz] and *J*(RhP) coupling indicative of a Rh(iii) centre [*J*(RhP) = 112–121 Hz].^53^ In the ^1^H NMR spectrum (183 K) at least three different hydride multiplet environments are observed between *δ* −19.40 and −19.95 [*J*(RhH) = 29–31 Hz from selective decoupling], that combined integrate to a single proton.^55^ No H–H coupling is observed, which is different from dihydride **4-Me**.

Collectively these NMR data suggest complex **4-iPr** is formed as a mixture of at least three ^i^Pr-cyclometallated species, that interconvert on the NMR timescale at room temperature by a process that does not break and exchange the Rh–H bond. Reversible reductive elimination and exchange with other C–H groups in the ligand would be expected to result in loss of the hydride signal and associated coupling if it occurred on the NMR timescale.^56–60^ We thus propose that this fluxional process is associated with a restricted P–C rotation^10,14^ of the bulky ^i^Pr-aryl groups that leads to different, but exchanging, rotamers^61^ of the same *ortho*-metalled isomer. In the absence of a single-crystal X-ray structure we cannot definitively assign a structure to **4-iPr** as one where the ^i^Pr methine or methyl group has undergone C–H activation, and both motifs are known.^62^ While we cannot unequivocally rule out a ground-state structure arising from methine-^i^Pr activation, we favour methyl activation as the hydride peaks correlate to methyl, aromatic and methine signals in the low temperature NOESY spectrum (ESI†). Very similar spectra are obtained on hydrogenation in 1,2-F_2_C_6_H_4_ or *o*-xylene solvent (ESI†), meaning there is no evidence for significant solvent coordination at the Rh(iii) centre, or agostic interactions, the latter albeit expected to be weak.^63,64^ The hydride is located *trans* to the coordinated oxygen on the basis of the observed chemical shift.^65^

**Scheme 3:**
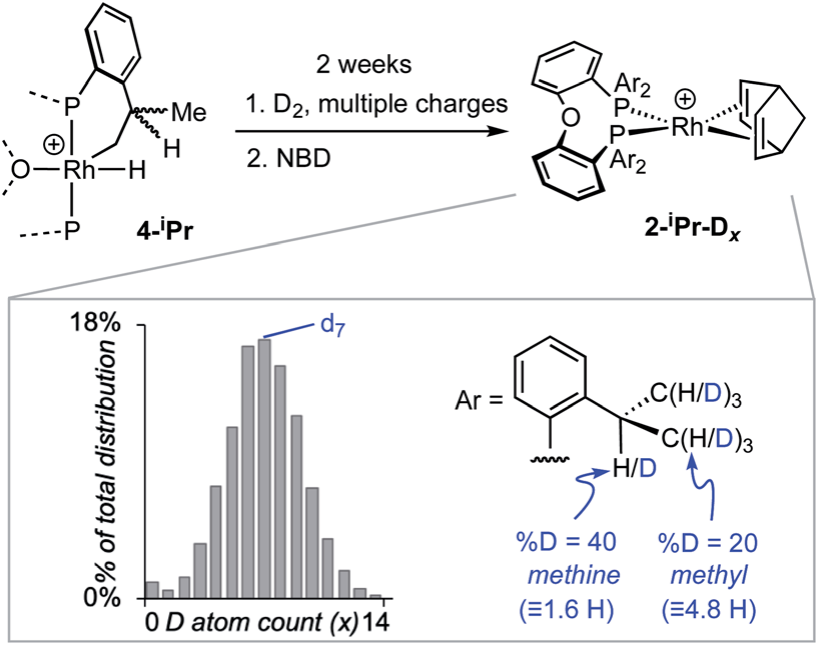
H/D exchange in **4-iPr** and trapping with NBD to form **2-iPr-Dx**. Inset shows the distribution of isotopologues of **2-iPr-Dx** as measured by ESI-MS and analysed using an in-house Python script. [BAr^F^_4_]^−^ anions not shown.

**Scheme 4:**
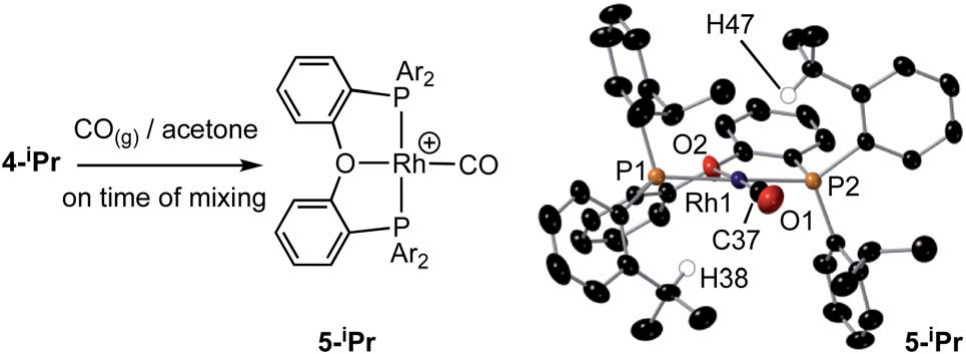
Reaction of **4-iPr** with CO, and solid-state structure of **5-iPr** highlighting the position of anagostic contacts. [BAr^F^_4_]^−^ anions not shown. Displacement ellipsoids are shown at the 50% probability level. Rh1–P1, 2.3145(7); Rh1–P2, 2.3027(8); Rh1–C37, 1.819(4); Rh1–O2, 2.128(3); Rh–H38, 2.821; Rh–H47, 2.627; P1–Rh1–P2, 162.41(4); O2–Rh1–C1, 177.0(1).

While reversible cyclometallation of **4-iPr** is not observed on the NMR timescale, it does occur on the laboratory timescale as probed by a variety of experiments, Schemes 3 and 4:

(i) Addition of NBD quantitatively reforms **2-iPr** on time of mixing.

(ii) Repeated charging of an *o*-xylene solution of **4-iPr** over two weeks with D_2_ results in a significant, but slow, reduction in intensity of the hydride signal and the concomitant appearance of signals in the hydride and alkyl regions of the ^2^H NMR spectrum. Subsequent addition of NBD results in the formation of **2-iPr-Dx**, that could be reliably analysed using electrospray ionisation mass spectrometry (ESI-MS) and NMR spectroscopy. Processing of the resulting isotope pattern for the cation in **2-iPr-Dx** (ESI†) reveals a distribution of isotopologues, *x* = 0 to 14, centred around *x* = 6 to 8 (Scheme 3). That both methyl and methine C–H activation occurs is demonstrated in the ^1^H NMR spectrum of **2-iPr-Dx** that shows a reduction in intensity for both these environments, corresponding to 20% D and 40% D incorporation respectively (4.8 D and 1.6 D respectively). No H/D exchange is observed in the C–H bonds of the aryl groups.^66^**4-Me** undergoes no exchange under the same conditions.

(iii) Addition of CO to **4-iPr** results in the quantitative formation of the Rh(i) complex [Rh(κ^3^-P,O,P-DPEphos-^i^Pr)(CO)][BAr^F^_4_], **5-iPr**, the structure of which has been determined by single-crystal X-ray diffraction (Scheme 4). Complex **5-iPr** has two anagostic C–H⋯Rh interactions, similar to **2-iPr**, but now from two methine C–H groups (H38, 2.821 Å, *ø* = 59.0°; H47, 2.671 Å, *ø* = 64.0°). In solution at 298 K the cation displays time averaged *C*_2v_ symmetry by NMR spectroscopy. Two methine environments are observed in the ^1^H NMR spectrum, one shifted significantly downfield from the other: *δ* 4.74 and 3.10 (2 H integral each), and the former signal is assigned to the anagostic pair H38/H47 (*δ*_calc_ = 4.6).

**Scheme 5:**
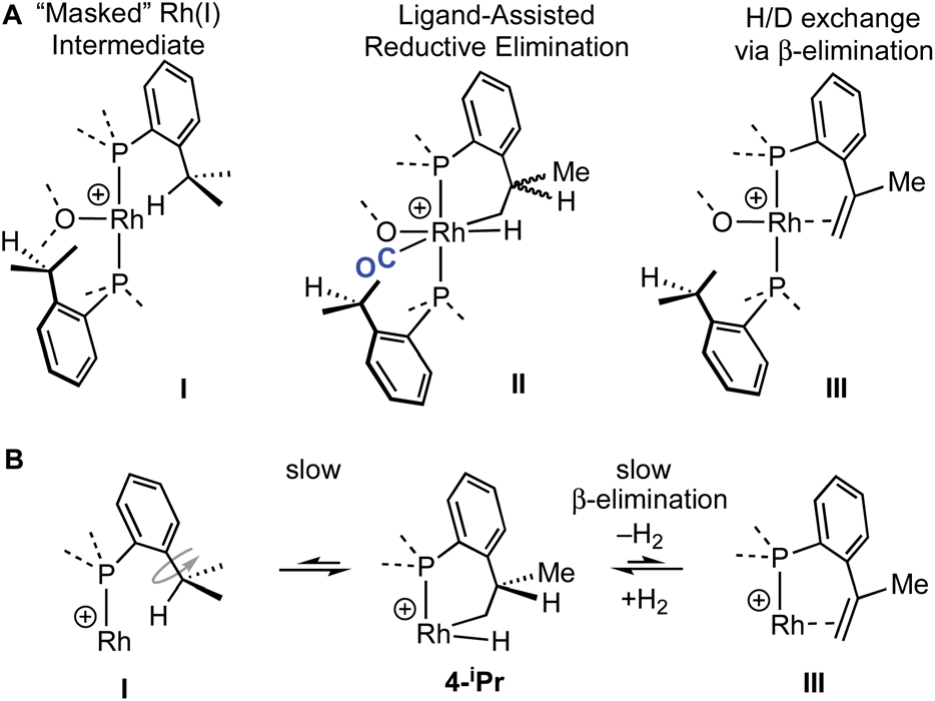
(A) Possible intermediates for the formation of Rh(i) complexes, and H/D exchange starting from **4-iPr**. (B) Proposed mechanism for H/D exchange.

The reaction of **4-iPr** with CO and NBD on time of mixing indicates that this Rh(iii) complex acts as a “masked”^57^ source of Rh(i). While this suggests a kinetically accessible Rh(i) intermediate could be in equilibrium with **4-iPr** (**I**, Scheme 5), invoking this as the only accessible intermediate would not account for H/D exchange observed on addition of D_2_ nor the fluxional process observed on the NMR timescale that retains the Rh–H bond. Alternatively, ligand-assisted reductive elimination^67–69^ from a Rh(iii) intermediate (**II**) could result in the direct formation of a Rh(i) product without involving **I**, to give **2-iPr** (NBD) or **5-iPr** (CO, shown).

The lack of H/D exchange for **4-Me** suggests that if C–H oxidative addition does operate for this complex, subsequent exchange with D_2_ at the Rh(iii) centre^70^ (*e.g. via* a σ-CAM process^71^) must be a high energy, inaccessible process. By extension, the H/D exchange observed in **4-iPr** likely also proceeds by an alternative mechanism and we propose a β-elimination/dehydrogenative process *via* an intermediate such as **III**, as previously used to explain, albeit faster, well-defined reversible C–H activation processes.^72,73^ Subsequent addition of D_2_ would then provide pathways for methine and hydride D-incorporation. An additional slower, reversible, reductive elimination to form **I** would account for both multiple methyl H/D exchanges within one ^i^Pr group and for more than one ^i^Pr group undergoing H/D exchange (*i.e.*, *d*_x_ > 7 Scheme 3).^66^ Consistent with this, HD_(dissolved)_ is also observed [*δ* 4.39, *J*(HD) = 43 Hz]. The overall very slow H/D exchange indicates relatively significant barriers operate for the formation of **I**, consistent with the observation of an intact Rh–H group on the NMR timescale.

**Scheme 6:**
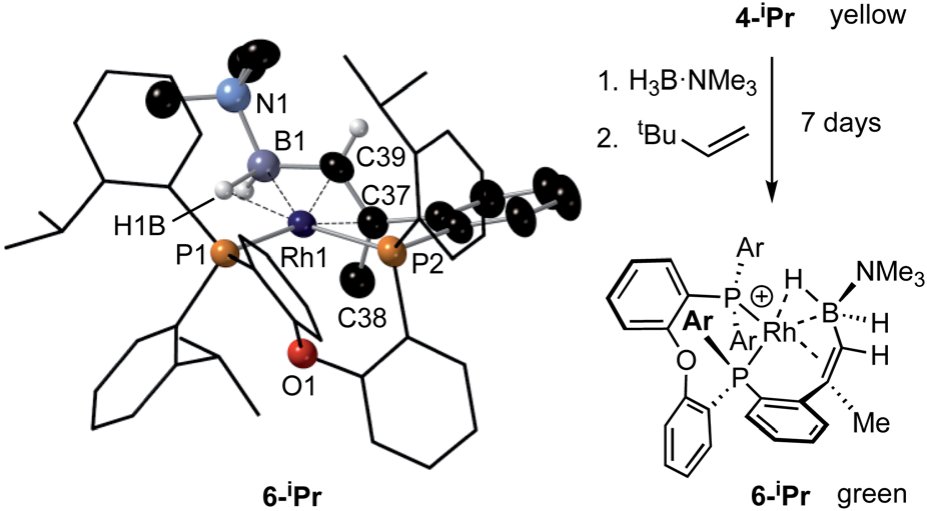
Synthesis and solid-state structure of **6-iPr**. Displacement ellipsoids are shown at the 30% probability level. Selected bond distances and angles: Rh1–P1, 2.3361(9); Rh1–P2, 2.2696(11); Rh1–B1, 2.391(6); Rh1–C37, 2.266(3); Rh1–C39, 2.152(3); Rh1–H1B, 1.99(5); B1–C39, 1.557(6); C37–C38, 1.511(6); C37–C39, 1.392(6); P1–Rh1–P2, 100.59(4); B1–C39–C37, 123.2(3); Rh1–H1B–B1, 87.8(18).

While the intermediate **III** has not been observed, indirect evidence that it is kinetically accessible comes from the reaction of **4-iPr** with H_3_B·NMe_3_ and the hydrogen acceptor *tert*-butyl ethene (*t*be). This, slowly (7 days), but cleanly, forms a new product, in which a cyclometallated ^i^Pr-group has formally undergone a double-dehydrogenative borylation^74–76^ with H_3_B·NMe_3_ to form a Rh(i) vinylborane complex [Rh(κ^2^-P,P-(DPEphos-^i^Pr″)-η^2^-BH_2_NMe_3_)][BAr^F^_4_], **6-iPr**, which is isolated in good yield (88%) as a green analytically pure solid. The solid-state structure of **6-iPr** is shown in Scheme 6. This reveals a Rh(i) centre complexed with a chelating vinyl amine-borane [C39–C37, 1.392(6) Å] that coordinates to the Rh(i) centre through the alkene and a non-classical B–H 3c-2e agostic^28^ bond[Rh⋯H1B, 1.99(5); Rh⋯B, 2.391(6) Å]. This last distance is suggestive of an η^2^-interaction of the B–H bond with the Rh(i) centre, underscored by the rather closed Rh–H1B–B angle, 87.8(18) Å.^77,78^ The Rh–P bond opposite the weaker *trans*-influence B–H agostic bond is correspondingly shorter than that opposite the alkene. Room temperature NMR data are fully consistent with the crystallographically determined structure, showing two inequivalent, mutually coupled, environments in the ^31^P{^1^H} NMR spectrum. In the ^1^H{^11^B} NMR spectrum a relative integral 1H vinyl [*δ* 3.86], B–H(terminal) [*δ* 1.89, d, *J*(HH) 14 Jz; *δ*_calc_ = +2.2] and agostic B–H⋯Rh [*δ* −7.54, *J*(RhH) 14, *J*(PH) 52, *J*(HH) 14 Hz; *δ*_calc_ = −6.4] are observed. The agostic B–H signal is significantly upfield shifted compared to both the terminal B–H and the free vinyl borane PhCH=CPh(BH_2_·NMe_3_), *δ* 2.40.^79^ The ^11^B{^1^H} NMR spectrum shows a very broad signal centred at *δ* −10.2 assigned to the borane. The chelating motif of the amine-borane in **6-iPr** is similar to that reported for RuH_2_{η^2^,η^2^-HCHB(N^i^Pr_2_)CH_2_C_6_H_4_PPh_2_}(PCy_3_), **A**, which also shows a similar chemical shift for the η^2^-M⋯H–B interaction in the ^1^H spectrum.^80^ The M⋯B distance in **6-iPr** is longer however, [2.391(6) *versus* 2.173(3) Å] reflecting that there is no vacant p-orbital on boron available for back donation from the metal, unlike for **A**.

**Fig. 9:**
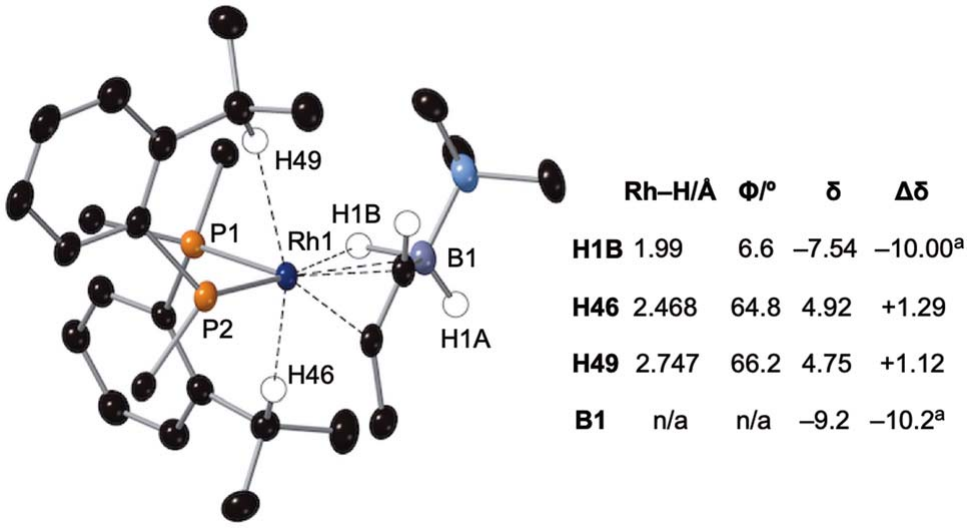
Comparison of selected structural and spectroscopic data for the anagostic/B–H agostic interactions in **6-iPr**. Selected aryl groups are removed for clarity. ^a^Chemical shifts compared with the vinyl borane PhCH=CPh(BH_2_·NMe_3_).^79^

Particularly noteworthy in the ^1^H NMR spectrum of **6-iPr** are two downfield shifted signals (1H relative integral each) at *δ* 4.92 and 4.75 (*δ*_calc_ = 5.2 and 4.7), which are comparable to the signals assigned to anagostic C–H hydrogens in **5-iPr**. Closer inspection of the solid-state structure shows that the methine C–H protons H49 and H46 are in close approach to the Rh(i) centre and orientated above and below the RhP2B1 plane, Fig. 9, (*ø* = 66.2° and 64.8°). In comparison, the upfield shifted signal, at *δ* −7.54, is due to the agostic 3c-2e Rh⋯H–B motif that sits squarely in the RhP_2_ plane (*ø* = 6.6°). **6-iPr** thus highlights, in a single complex, the relationship between the orientation of the approaching E–H bond to the metal centre: the C–H anagostic interaction lying above the d^8^ metal coordination plane and the 3c-2e B–H→Rh agostic bond sitting within the coordination plane.

Selected data from the computational analysis of **[6-iPr]+** are shown in Table 3 and suggest the Rh⋯H46 interaction is similar in strength to the Rh⋯H1 interaction in **[2-iPr]+**. Both these C–H⋯Rh anagostic interactions exhibit relatively weak 
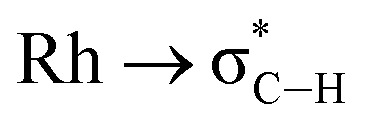
 donation. In contrast the 3c-2e B–H⋯Rh agostic motif is markedly stronger and is now dominated by very strong donation from an occupied B–H orbital into an unoccupied Rh-orbital that NBO analysis quantifies at 52.4 kcal mol^−1^, *i.e.* a 3c-2e bond. This is significantly stronger than in the related [(NNN)Rh(H_3_BNMe_3_)]^+^ adduct (NNN = 2,6-bis-[1-(2,6-diisopropyl-phenylimino)ethyl]pyridine),^77^ consistent with a much shorter computed Rh⋯H distance (1.78 A *cf.* 1.91 A) and longer B–H distance (1.35 A *cf.* 1.28 A) in **6-iPr**.

**Table 3:** Computed metrics for X–H⋯Rh (X = B, C) interactions in **[6-iPr]+**

	Distance/Å	*ρ*(*r*)^a^	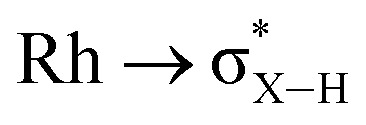 b	σ_X–H_ → Rh
Rh⋯H1B	1.78	0.083	6.71	52.38
Rh⋯H46	2.38	0.024	10.95	0.95
Rh⋯H49	2.71	0.015	9.59	0.73

a BCP electron densities in au.

b NBO donor–acceptor interactions in kcal mol^−1^.

**Scheme 7:**
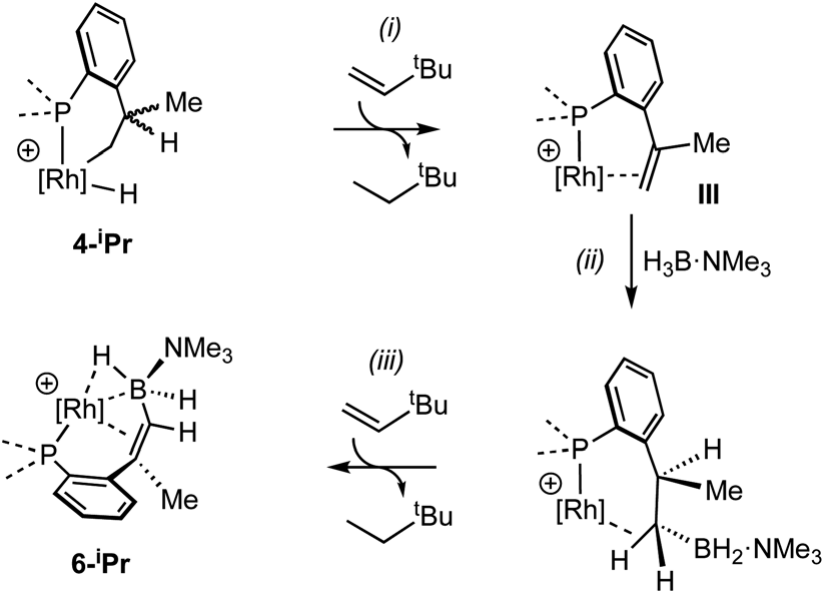
Suggested outline mechanism for the dehydrogenative borylation **4-iPr**. Only key ligands shown. [BAr^F^_4_]^−^ anion omitted for clarity.

A suggested, abbreviated, mechanism for the formation of **6-iPr** is shown in Scheme 7: (i) dehydrogenation of an ^i^Pr group gives intermediate **III** (Scheme 5),^72,81^ (ii) hydroboration of the alkene using H_3_B·NMe_3_;^82^ (iii) followed by dehydrogenation, *via* C–H activation/β-elimination.^75,76^ Throughout tbe acts as a sacrificial hydrogen acceptor. While this scheme captures the gross transformations, the precise order of events currently remains unresolved.

## Conclusions

We have shown that aryl-group *ortho*-substitution in [Rh(NBD)(DPEphos-R)]^+^ leads to differences in structures, fluxional processes and reactivities – which can be related to the steric bulk of the *ortho*-group. Broadly speaking, OMe and Me substituents lead to solid-state and solution structures that are not too dissimilar to parent DPEphos. With the ^i^Pr group fluxional processes in solution are retarded, and C–H activation processes occur. DPEphos-^i^Pr thus cannot be considered an innocent ligand, this being related – more broadly – to the decomposition pathways of parent DPEphos that occur *via* C–O bond cleavage.^27,83^

**Fig. 10:**
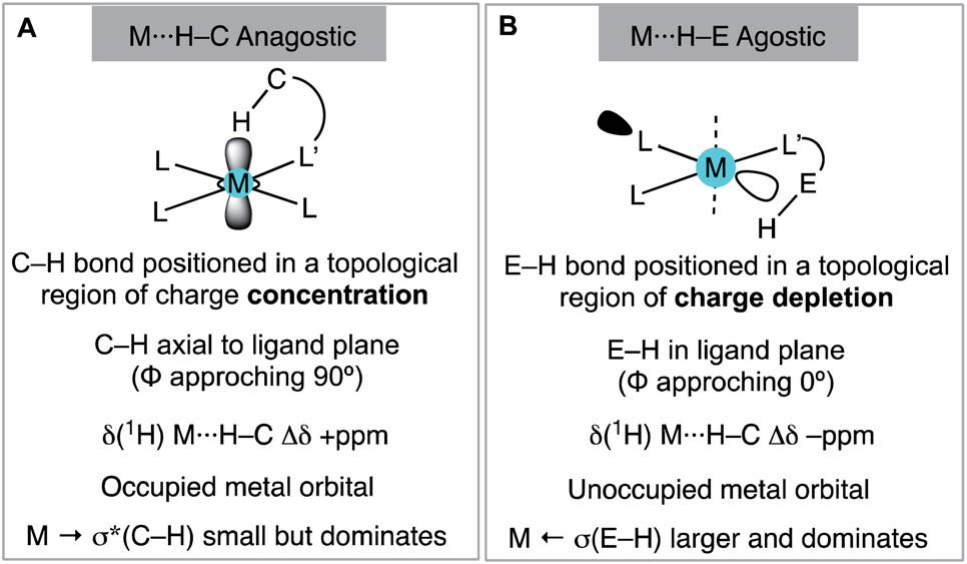
Structural, electronic and NMR properties of anagostic interactions (A) and E–H agostic bonds (B), as based upon Scherer's analysis.

Common to all the Rh(i) DPEPhos-R complexes structurally described herein (with their associated NBD, CO or vinylborane co-ligands) is the observation of downfield-shifted signals in their ^1^H NMR spectra that signal an anagostic M⋯H–C interaction,^28^ for which the steric bulk of the ligand determines the temperature at which they are observed. As discussed previously,^30,38,40,45^ while such anagostic interactions are associated with weak 
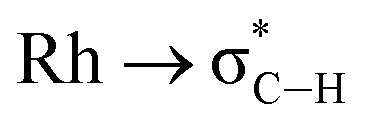
 and minimal σ_C–H_ → Rh orbital donations, the driver for the downfield chemical shift of the C–H protons observed in the ^1^H NMR spectrum does not come from these. Instead, the positioning of the anagostic hydrogen with reference to different regions of valence shell for the d^8^ metal centre is important, as Scherer^30^ has previously elegantly described for Rh(CAAC)(CO)Cl systems (CAAC = cyclic alkyl-aminocarbene). Our observations here, on a consistent set of complexes, reinforce this analysis. Thus, when the hydrogen atoms are forced, through steric constraints, to sit in an axial position (*ø* approaching 90°) that places them above a region of charge concentration, the associated magnetically-induced current density results in a downfield shift in the NMR spectrum, Fig. 10A. This analysis differentiates anagostic interactions from 3c-2e agostic bonds, the latter being characterised by upfield shifts in their ^1^H NMR spectra due to the associated hydrogen atoms being located in a region of charge depletion in the ligand plane of a d^8^ ML_3_ type fragment (Fig. 10B, *ø* approaching 0°). Complex **6-iPr** offers E–H bonds (E = C, B) in both these topologies, and thus shows both upfield and downfield chemical shifts in the ^1^H NMR spectrum. While, as for **6-iPr**, any agostic bond will likely show a significantly stronger 3c-2e σ_X–H_ → Rh interaction compared to the weak 
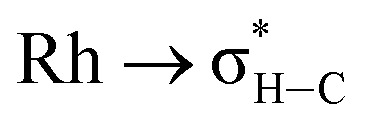
 donation associated with the anagostic interactions, the relationship, if any, between these bonding descriptors and the observed chemical shift has yet to be demonstrated.

These observations reinforce the analysis that the chemical shift changes observed by ^1^H NMR spectroscopy in d^8^ square planar complexes with anagostic C–H bonds located above the ligand plane result from topologically enforced ring current effects, rather than signalling an interaction that has a considerable orbital contribution. In this regard they are perhaps more related to the chemical shift changes that are well-established for protons that are forced to sit in topologically distinct regions close to arenes.^30,84,85^ We thus suggest there is a clear demarcation between anagostic interactions, and agostic, 3c-2e, bonds; differences that arise from both the topological orientation and the nature of the orbital interactions that prevail for each.

## Data availability

All data is in the ESI. There is no more to deposit.

## Author contributions

J. J. R.: conceptualisation, experimentation, data analysis, drafting the manuscript; A. L. B.: conceptualisation and computational analysis; T. M. B., A. H. and A. M. M.: NMR data fitting and single crystal X-ray analysis; S. A. M., A. S. W.: conceptualisation, project coordination, writing the final manuscript with contributions from all authors.

## Conflicts of interest

There are no conflicts to declare.

## Supplementary Material

SC-012-D1SC01430G-s001

SC-012-D1SC01430G-s002

SC-012-D1SC01430G-s003
