# Supplementary material for: Ortho-aryl substituted DPEphos ligands: rhodium complexes featuring C–H anagostic interactions and B–H agostic bonds
Source: Chem Sci. 2021 May 25;12(25):8832–43. doi: 10.1039/d1sc01430g (PMC8246285; doi:10.1039/d1sc01430g)
Supplement: SC-012-D1SC01430G-s001 [file SC-012-D1SC01430G-s001.pdf]

Electronic Supplementary Material (ESI) for Chemical Science.
This journal is © The Royal Society of Chemistry 2021

Supporting Information

Ortho-aryl substituted DPEphos ligands: Rhodium Complexes Featuring C-H Anagostic and B-H Agostic Motifs

James J. Race,[a,b] Arron L. Burnage,[c] Timothy M. Boyd,[a,b] Alex Heyam,[b] Antonio Martinez-Martinez,[b] Stuart A. Macgregor,[c]* Andrew S. Weller[a]*

[a.] Department of Chemistry, University of York, Heslington, York, YO10 5DD (UK).

[b.] Chemistry Research Laboratories, University of Oxford, Oxford OX1 3TA (UK)

[c.] Institute of Chemical Sciences, Heriot Watt University, Edinburgh EH14 4AS (UK)

Table of Contents

General experimental methods 3
Synthesis of ligand 1-^{i}Pr and complexes 2-R 4
  1-^{i}Pr 4
  2-H, 2-Me and 2-OMe 8
  2-^{i}Pr 11
Structural Data of Complexes 2-R 12
Variable temperature solution NMR spectroscopy of 2-R 13
  2-H 13
  2-Me 15
  2-OMe 17
  2-^{i}Pr 19
  Modelling of fluxional behaviour in 2-Me and 2-OMe 20
Identification of Anagostic Motifs 23
  Aryl anagostic coupling constants 23
  Assignment of *ortho*-hydrogen atoms in free ligands 1-R 25
  Difference in chemical shift in free ligand and in anagostic motifs 33
Computational Studies: Structures, Chemical Shifts and Bonding 35
  Computational Details 35
  Electronic Structure Analysis 36
    Computed Atomic Charges 50
  Comparison of BCP Metrics with Rh···H distances 52
  Computed NMR Data 54
Hydrogenation of 2-Me 56
Hydrogenation of 2-OMe 61
Formation of 4-^{i}Pr 64
Reformation of 2-^{i}Pr by addition of NBD to 4-^{i}Pr 69
Deuterium Incorporation into 4-^{i}Pr 71
Formation of [Rh(κ^{3}-P,O,P-DPEphos-^{i}Pr)(CO)][Bar$^{F}{}_{4}$] 5-^{i}Pr 73
Formation of [Rh(κ^{2}-P,P-(DPEphos-^{i}Pr'-η^{1}-BH_2NMe_3)][BAr$^{F}{}_{4}$] 6-^{i}Pr 76
Crystallographic and Refinement Data 79
  Tables of data 80
  Additional Comments on Crystal Structures and Refinement Data 82
References 83

General experimental methods

All experiments were performed under an atmosphere of argon, using standard Schlenk techniques on a dual vacuum/inlet manifold unless specified. Glassware was dried in an oven at 140 °C overnight or flame dried under vacuum prior to use. Pentane, hexane, THF, diethyl ether and CH_2Cl_2 were dried using an MBraun SPS-800 solvent purification system and degassed by three freeze-pump-thaw cycles. 1,2-difluorobenzene was stirred over Al_2O_3 for two hours and then CaH_2 overnight before vacuum transfer and subsequent degassing by three freeze-pump-thaw cycles. Dichloromethane-D_2 (CD_2Cl_2) was dried overnight with CaH_2 and acetone-D_6 with K_2CO_3 before vacuum transfer and subsequent degassing by three freeze-pump-thaw cycles and was stored over 3 Å molecular sieves. *O*-xylene was stored over 3 Å molecular sieves**.**

$[RhCl(NBD)]_2$ and $[Rh(NBD)_2][Bar^F{}_4]$ (NBD = norbornadiene, $[Bar^F{}_4]^-$ = B(3,5–$(CF_3)_2C_6H_3)_4$) were prepared via the literature procedures.[1] All other reagents were purchased from commercial vendors and used as received. *o*-Me-DPEphos **1-Me** was purchased from Merck and used as received.

NMR data was collected on either a Bruker 400 MHz, Venus400, a Bruker Ascend 400 MHz spectrometer or Bruker 500 MHz AVC. Residual protio solvent resonances were used as a reference for ^1H NMR spectra. $^{31}P\{^1H\}$ NMR spectra were referenced externally to 85 % H_3PO_4. All chemical shifts (δ) are quoted in ppm and coupling constants in Hz.

Electrospray ionization mass spectrometry (ESI-MS) was carried out using a Bruker MicroTOF instrument directly connected to a modified Innovative Technology glovebox[2] unless otherwise specified. Typical acquisition parameters were used (sample flow rate 4 μL min^{-1}, nebulizer gas pressure: 0.4 bar, drying gas: argon at 333 K flowing at 4 L min^{-1}, capillary voltage: 4.5 kV, exit voltage: 60 V). The spectrometer was calibrated using a mixture of tetraalkyl ammonium bromides $[N(C_nH_{2n+1})_4]Br$ (n = 2-8, 12, 16 and 18). Samples were diluted to a concentration of 1 x 10^{-6} M in the specified solvent before sampling by ESI-MS.

Elemental analyses were conducted by Mr. Stephan Boyer at London Metropolitan University and Graeme McAllister at the University of York.

Synthesis of ligand 1-^{i}Pr and complexes 2-R

1-^{i}Pr

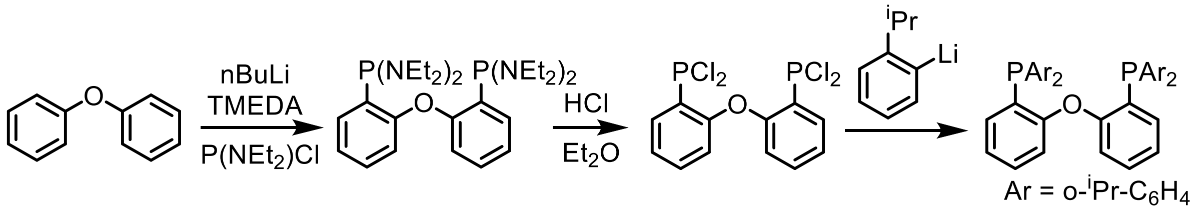

Scheme S1. Synthetic procedure to form **1-^{i}Pr**.

The synthesis followed literature procedures to form the chlorodiphosphine.[3, 4] In a separate flask the aryllithium reagent (1.2 equiv) was prepared at -78 °C from the addition of TMEDA (0.46 ml, 3.1 mmol) to *n*-butylithium (2.5 M in hexanes, 1.04 ml, 2.6 mmol) then dropwise addition of 1-bromo-2-isopropylbenzene (0.39 ml, 2.6 mmol) and the mixture was left to stir at –78 °C for 1 hour. Dropwise addition of this aryl lithium solution to the chlorophosphine (200 mg, 0.54 mmol) in THF (5 ml) also kept at -78 °C formed a clear orange solution after 1 hour of stirring at -78 °C. Methanol (0.5 ml) was added at -78 °C and the mixture was warmed to room temperature (ligand is air stable). Saturated [NH_4]Cl solution (10 ml) was added and the mixture was separated, and the organic phase extracted with pentane (3 x 10 ml). The organic phases were collated and the solvent removed *in vacuo* to yield the crude product as a pale yellow solid which was recrystallised in hot pentane to yield pure **1-^{i}Pr** as a white solid (115 mg, 0.17 mmol, 30% yield).

^{31}P{^{1}H} NMR (162 MHz, $CDCl_3$): δ -37.6 ppm (s).

^{1}H NMR (400 MHz, $CDCl_3$, 298 K): δ 7.27 (m, 8H, Ar), 7.04 (m, 6H, Ar), 6.88 (m, 6H, Ar), 6.73 (ddd, *J* = 2, 4 and 8 Hz, 2H, Ar), 6.36 (dd, *J* = 4 and 8 Hz, 2H, Ar), 3.63 (septet, J_{HH} = 7 Hz, 4H, C*H*CH_3), 1.06 (d, J_{HH} = 7 Hz, 24H, CHCH_3).

^{13}C{^{1}H} NMR (126 MHz, $CDCl_3$, 298 K): δ 159.4 (d, J_{PC} = 19 Hz, Ar), 153.4 (d, J_{PC} = 28 Hz, Ar), 134.4 (s, Ar), 134.4 (s, Ar), 134.3 (s, Ar), 134.1 (s, Ar),129.8 (s, Ar), 128.9 (s, Ar), 125.7 (s, Ar), 125.1 (d, J_{PC} = 5 Hz, Ar), 123.2 (s, Ar), 118.0 (s, Ar), 31.1 (d, J_{PC} = 27 Hz, CH) and 23.9 (d, J_{PC} = 10 Hz, CH_3).

EI-MS (acetonitrile): *m/z* $[M]^+$ 706.4 (calc. 706.35) with the correct isotope pattern.

Multiple samples were submitted for elemental analysis, but no results were within 0.4% of the theoretical percentage mass by weight for carbon or hydrogen. Persistent pentane may be the cause of the inconsistent elemental analysis (see ^{1}H NMR spectrum below).

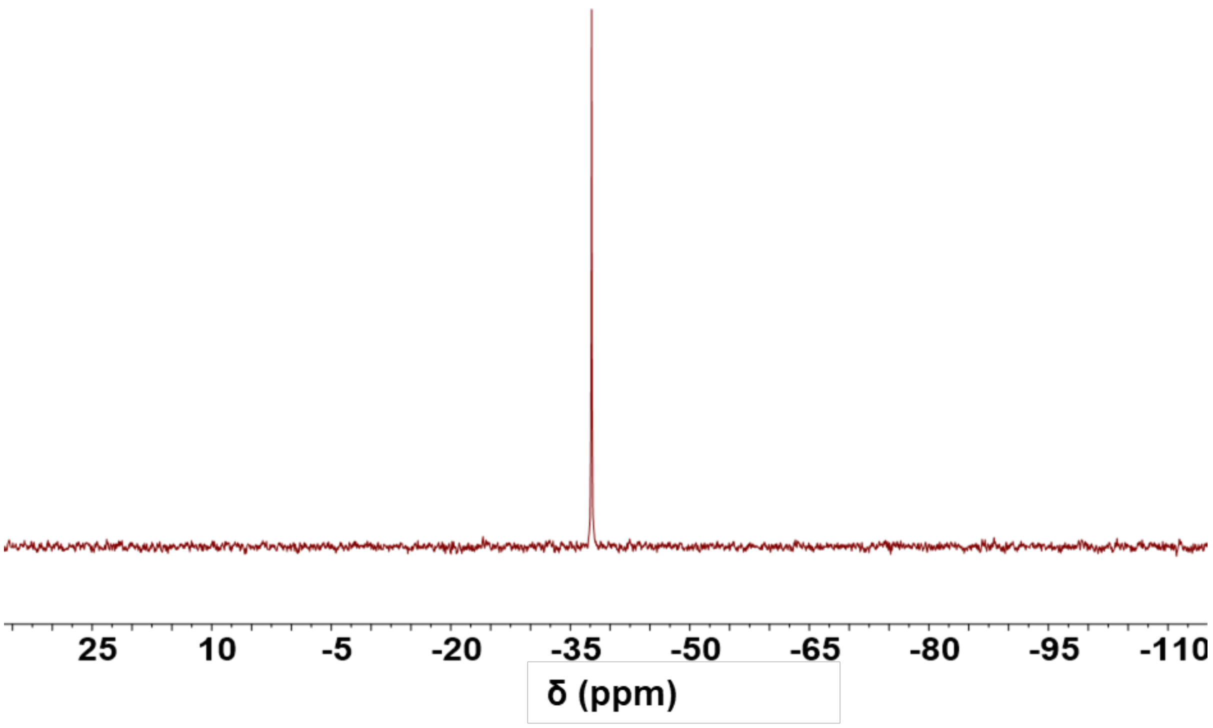

Figure S1. $^{31}P\{^{1}H\}$ NMR spectrum of **1-^{i}Pr** ($CDCl_3$, 162 MHz, 298 K).

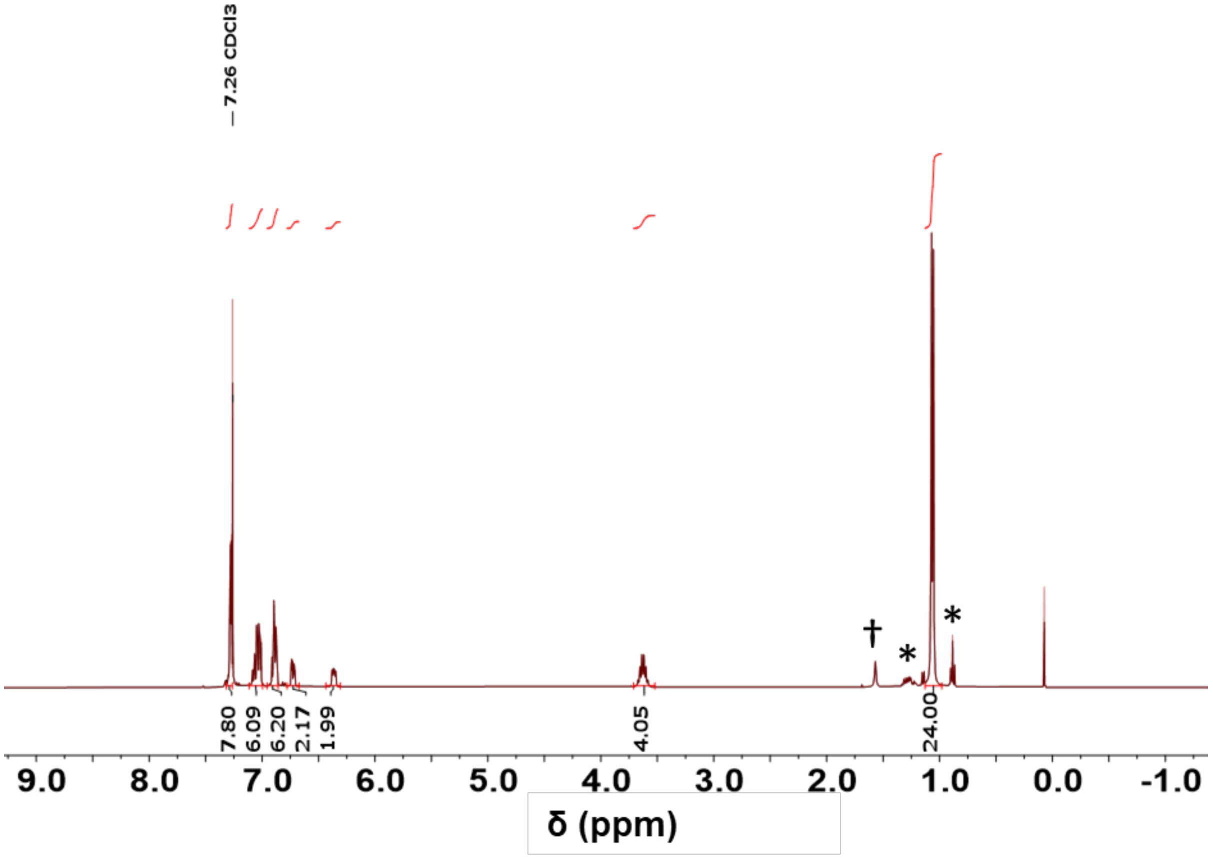

Figure S2. ^{1}H NMR spectrum of **1-^{i}Pr** ($CDCl_3$, 400 MHz, 298 K). Integrals relative to $[BAr^{F}{}_{4}]^{-}$ signals. * Denotes persistent pentane from recrystallisation and † indicates water impurity.

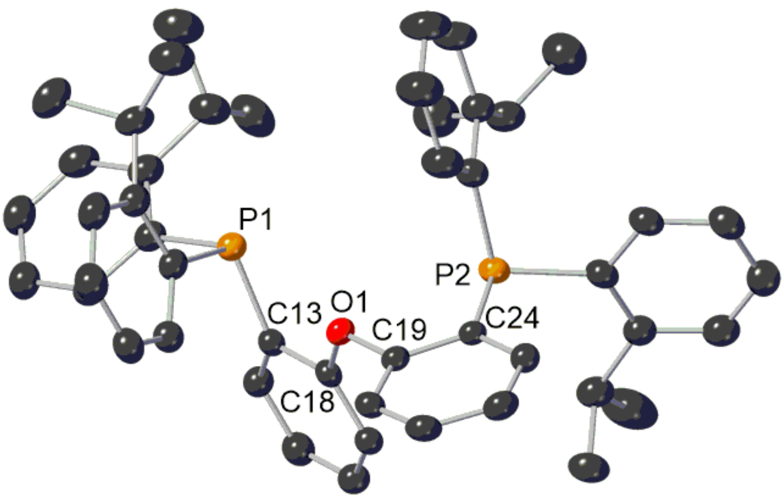

Figure S3. Molecular structure of **1-^{i}Pr** determined by single-crystal x-ray diffraction. Ellipsoids presented at 50% probability level. Key bond lengths and angles given in Table S1.

Table S1. Selected bond lengths and angles from the crystallographically determined structure of **1-^{i}Pr**.

**Bond/Angle**	**1-^{i}Pr**
**P1-C13 (Å)**	1.828(3)
**C13-C18 (Å)**	1.403(4)
**C18-O1 (Å)**	1.386(4)
**O1-C19 (Å)**	1.390(4)
**C19-C24 (Å)**	1.396(5)
**C24-P2 (Å)**	1.839(3)
**C18-O1-C19 (°)**	1.218(4)

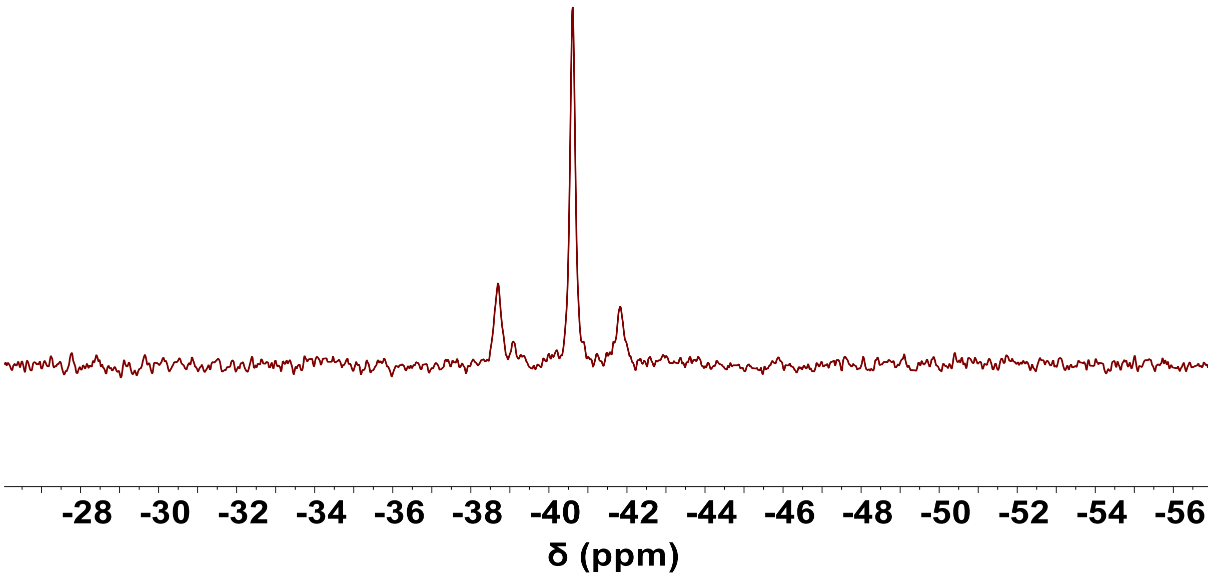

Figure S4. $^{31}P\{^1H\}$ NMR spectrum of **1-^{i}Pr** at 203 K (CD_2Cl_2, 202 MHz, 203 K).

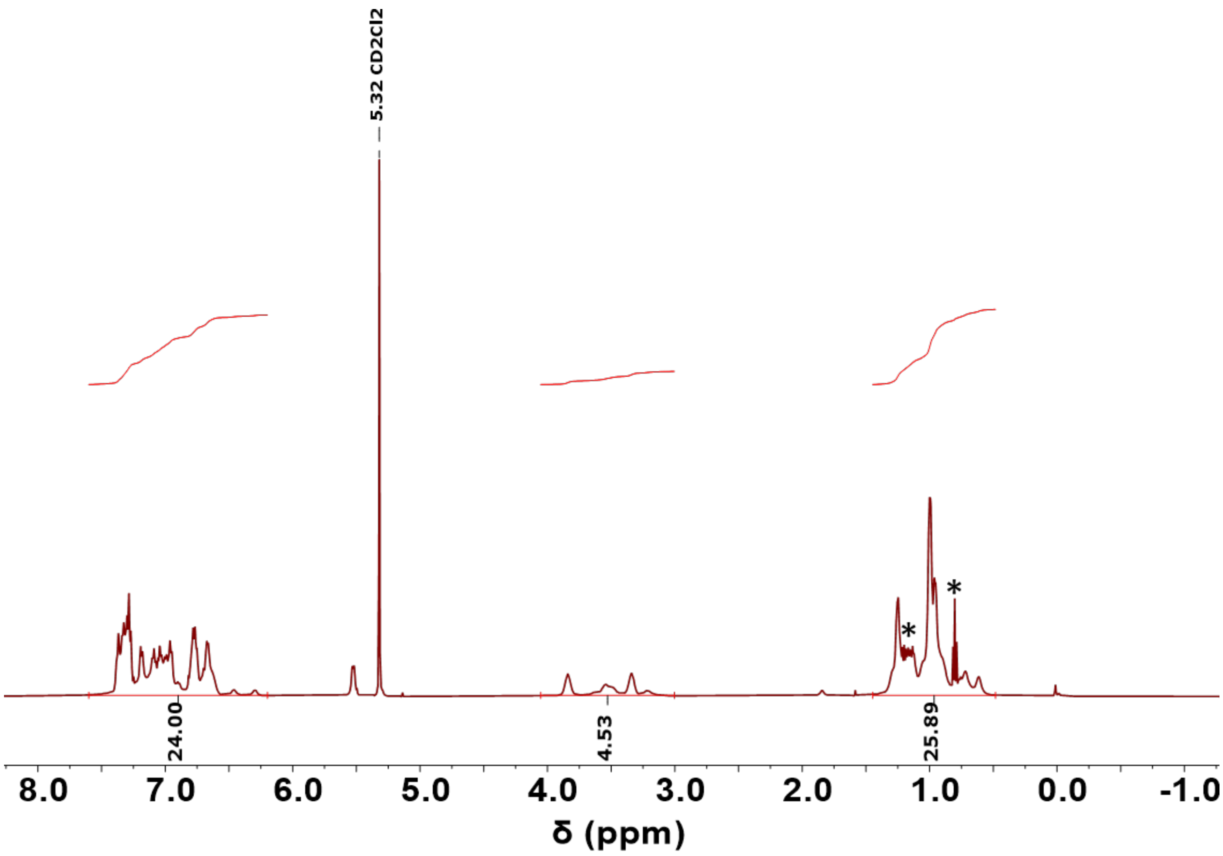

Figure S5. ^{1}H NMR spectrum of **1-[i]Pr** at 203 K (CD_2Cl_2, 500 MHz, 203 K). Integrals relative to the total of the aromatic signals and * denotes pentane impurity.

2-H, 2-Me and 2-OMe

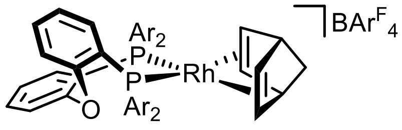

Ar = Ph **2-H**, *o*-Me-C_6H_4 **2-Me**
or *o*-OMe-C_6H_4 **2-OMe**

Ligand **1-H**, **1-Me** or **1-OMe** (200 mg) in 1,2-difluorobenzene (2 ml) was added dropwise to $[RhCl(NBD)]_2$ (0.5 equiv.) and left to stir at room temperature for one hour. This solution was then added dropwise to Na[BAr^F_4] (1 equiv.) in 1,2-difluorobenzene (2 ml) and stirred at room temperature for one hour which resulted in formation of a precipitate (NaCl). The solution was filtered, and the precipitate was washed with 1,2-difluorobenzene then the solvent removed *in vacuo* leaving an oily residue. The residue was washed with pentane (3 x 5 ml) and the resulting orange solid was dried under Schlenk line vacuum (< 1 x 10^{-1} mbar) overnight to leave a dry solid (all orange crystalline solids) that were stored in an argon glovebox.

2-H (85% yield): NMR data at 298 K as previously reported.[5]

$^{31}P\{^1H\}$ NMR (202 MHz, $CDCl_2F$, 140 K): δ 21.5 (br d, J_{RhP} = 154 Hz) and 13.8 (br d, J_{RhP} = 154 Hz).

^1H NMR (500 MHz, $CDCl_2F$, 140 K) selected data: δ 8.32 (br s, *ortho*-CH).

2-Me (75% yield):

$^{31}P\{^1H\}$ NMR (162 MHz, acetone-D_6, 298 K): δ 17.6 (br s), 8.5 (d, 154 Hz), 2.8 ppm (br s).

$^{31}P\{^1H\}$ NMR (162 MHz, acetone-D_6, 183 K): δ 18.0 (dd, J_{RhP} = 161 Hz, J_{PP} = 28 Hz) and 1.82 (dd, J_{RhP} = 152 Hz, J_{PP} = 28 Hz).

^1H NMR (400 MHz, acetone-D_6, 298 K): δ 7.78 (s, 8H, *o*-C*H* BAr^F_4), 7.66 (s, 4H, *p*C*H* BAr^F_4), 7.62-7.26 ppm (br m, 14H Ar), 7.25-7.02 (br s, 5H, Ar), 6.91 (m, 4H, Ar), 6.75 (br s, 1H, Ar), 4.11-3.31 (br s, 6H, sp^3-C*H* NBD (2H), sp^2-C*H* NBD (4H)), 2.23-1.51 (br s, 12H, CH_3), 1.38 (br s, sp^3-CH_2 NBD).

^1H NMR (400 MHz, acetone-D_6, 183 K): δ 10.27 (dd, J_{PH} = 17 Hz, J_{HH} = 7 Hz, 1H, *ortho*-H on substituted phenyl), 7.89 (s, 8H, *o*-CH BAr^F_4), 7.77 (s, 4H, *p*-C*H* BAr^F_4), 7.72-7.20 (m, 14H, Ar), 7.06 (m, 3H, Ar), 6.88 (m, 3H, Ar), 6.79 (dd, J_{HH} = 8 Hz, J_{PH} = 8 Hz, 1H, Ar), 6.68 (t, J_{HH} = 7 Hz, 1H, Ar), 6.16 (dd, J_{HH} = 8 Hz, J_{PH} = 12 Hz, 1H, Ar), 5.34 (s, 1H, sp^2-C*H* NBD), 4.05 (s, 1H, sp^3-C*H* NBD), 3.85 (s, 1H, sp^2-C*H* NBD), 3.71 (s, 1H, sp^3-C*H* NBD), 3.68 (s, 3H, CH_3), 3.43 (m, 2H, sp^2-C*H* NBD), 1.92 (br s, CH_3), 1.58 (s, CH_3) and 1.27 (m, CH_2 NBD).

^1H NMR (500 MHz, CD_2Cl_2, 203 K) selected data: δ 9.97 (dd, 1H, J_{PH} = 17 Hz, J_{HH} = 8 Hz, *ortho*-H on substituted phenyl), 3.56 (s, 3H, CH_3).

ESI-MS (1,2-difluorobenzene): *m/z* $[M]^+$ 853.18 (Calc. 853.17) with the correct isotope pattern.

Elemental analysis found (calc. for $C_{79}H_{56}BF_{24}OP_2Rh$): C 57.52 (57.40) H 3.53 (3.42).

2-OMe (78 % yield). At low temperature, a minor isomer is observed which we have assigned as an alternative anagostic motif**:**

$^{31}P\{^{1}H\}$ NMR (202 MHz, acetone-D$_6$, 298 K): δ 7.3 ppm (br s).

$^{31}P\{^{1}H\}$ NMR (202 MHz, acetone-D$_6$, 183 K) major isomer: δ 13.9 (dd, J_{PP} = 27 Hz, J_{RhP} = 164 Hz) and -0.6 (dd, J_{PP} = 27 Hz, J_{RhP} = 156 Hz), **minor isomer:** 11.2 (dd, J_{PP} = 28 Hz, J_{RhP} = 162 Hz) and -2.7 (dd, J_{PP} = 28z Hz, J_{RhP} = 158 Hz).

^{1}H NMR (500 MHz, acetone-D$_6$, 298 K: δ 7.79 (s, 8H, *o*-C*H* BAr$^F{}_4$), 7.67 (s, 4H, *p*-C*H* BAr$^F{}_4$), 7.53 (br s, 4H, Ar), 7.38-7.12 (m, 6H, Ar), 7.10-6.55 (m, 14H, Ar), 4.46-3.10 [broad overlapping signals, sp^2-C*H* NBD (4H), sp^3-C*H* NBD (2H) and OCH_3 (12H)] and 1.31 (s, 2H, CH_2 NBD).

^{1}H NMR (500 MHz, acetone-D$_6$, 183 K) major isomer: δ 9.53 (dd, J_{PH} = 16 Hz, J_{HH} = 7 Hz, 1H, *ortho*-H on substituted phenyl), 7.88 (s, 8H, *o*-C*H* BAr$^F{}_4$), 7.77 (s, 4H, *p*-C*H* BAr$^F{}_4$), 7.69-6.65 (complex multiplet, 21H, Ar), 6.57 (vt, J_{HH} = 7 Hz, 1H, Ar), 6.17 (vt, J_{HH} = 9 Hz, 1H, Ar), 4.24 (s, 3H, OCH_3), 4.20 (s, 1H, sp^2-C*H* NBD), 4.05 (s, 1H, sp^2-C*H* NBD), 3.82 (s, 3H, OCH_3), 3.81 (s, 1H, sp^2-C*H* NBD), 3.64 (s, 3H, OCH_3), 3.63 (br s, 2H, sp^2-C*H* and sp^3-C*H* NBD), 3.37 (s, 1H, sp^3-C*H* NBD), 3.26 (s, 3H, OCH_3) and 1.24 (s, 2H, CH_2), **selected data minor isomer**: δ 9.37 (dd, J_{PH} = 16 Hz, J_{HH} = 7 Hz, 1H, *ortho*-H).

^{1}H NMR (500 MHz, CD_2Cl_2, 203 K) selected data major isomer: δ 9.19 (dd, 1H, J_{PH} = 17 Hz, J_{HH} = 7 Hz, *ortho*-H on substituted phenyl), **selected data minor isomer**: δ 9.32 (dd, J_{PH} = 17 Hz, J_{HH} = 7 Hz, 1H, *ortho*-H).

ESI-MS (1,2-difluorobenzene): *m/z* $[M]^+$ 853.18 (Calc. 853.17) with the correct isotope pattern.

Multiple samples were submitted for elemental analysis, but no results were within 0.4% of the theoretical percentage mass for carbon or hydrogen. Persistent pentane after recrystallisation may be the cause of the inconsistent elemental analysis (see ^{1}H NMR spectrum below).

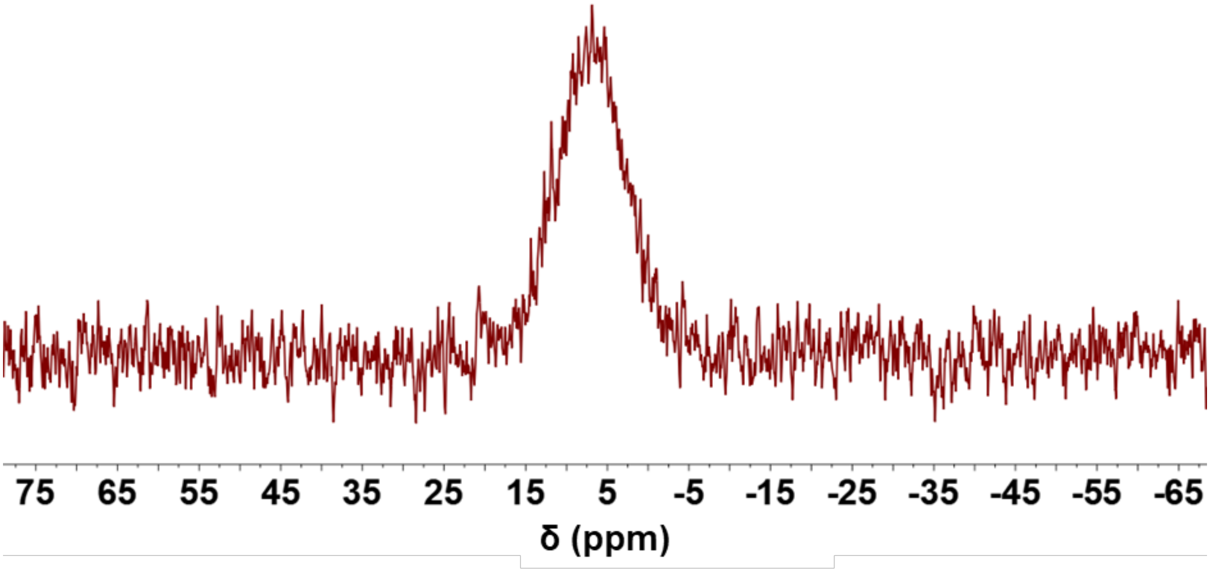

Figure S6. $^{31}P\{^{1}H\}$ NMR spectrum of **2-OMe** (acetone-D$_6$, 202 MHz, 298 K).

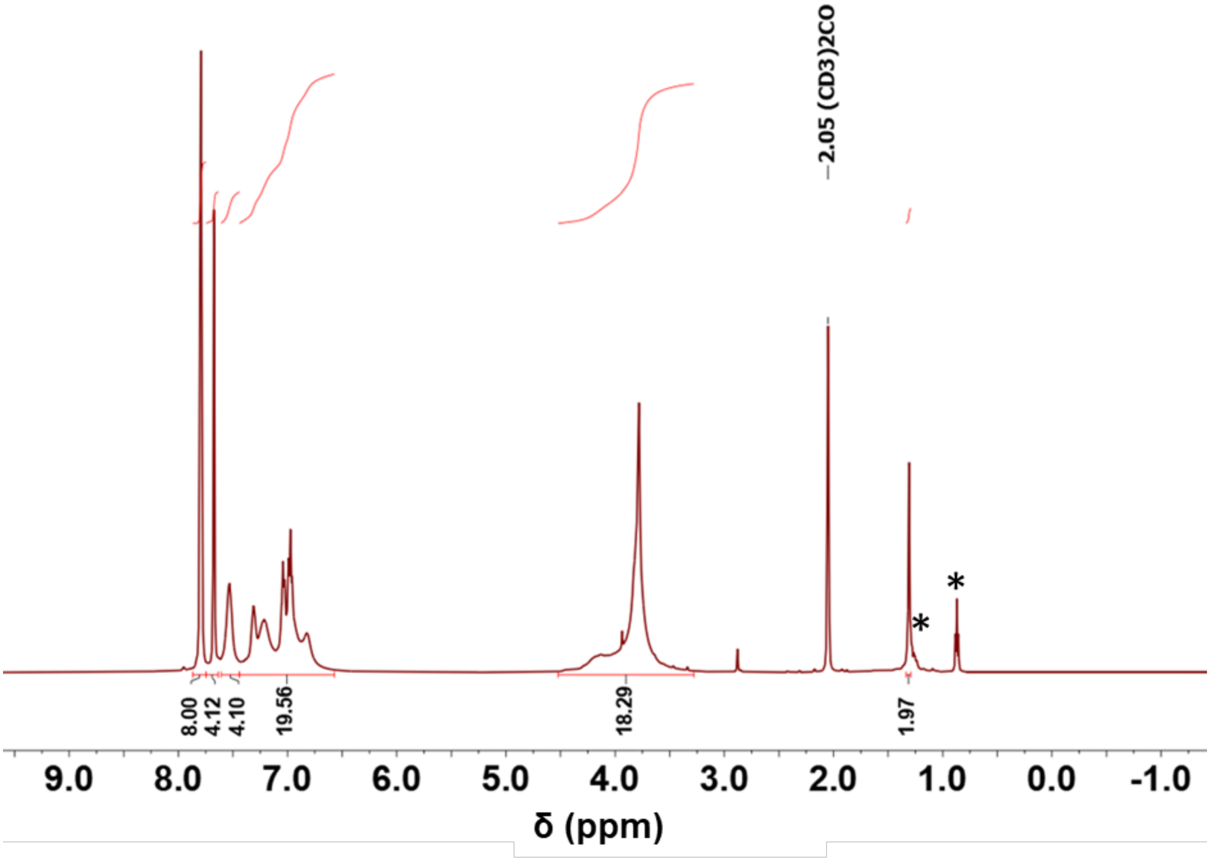

Figure S7. ^{1}H NMR spectrum of **2-OMe**. Integrals relative to $[BAr^{F}_{4}]^{-}$ signals (acetone-D_6, 500 MHz, 298 K). * Denotes persistent pentane impurity after recrystallisation.

2-^{i}Pr

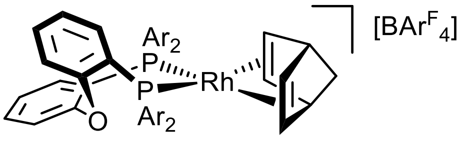

Ar = *o*-^{i}Pr-C_6H_4 **2-^{i}Pr**

Ligand **1-^{i}Pr** (151 mg, 0.21 mmol) in 1,2-difluorobenzene (2 ml) was added dropwise to [Rh(NBD)$_2$][Bar$^F_4$] (246 mg, 0.21 mmol) to form a dark red solution. The solvent was removed *in vacuo* to leave a purple residue that was dissolved in DCM (2 ml) and added dropwise to a stirring flash of pentane (20 ml) that produced an orange precipitate. The solid was filtered and washed with pentane (3 x 5 ml) and then dried under Schlenk line vacuum (< 1 x 10^{-1} mbar) overnight to leave an orange solid (241 mg, 0.14 mmol, 65% yield).

^{31}P{^{1}H} NMR (202 MHz, acetone-D$_6$, 298 K): δ 20.8 (d, J_{RhP} = 153 Hz).

^{1}H NMR (500 MHz, acetone-D$_6$, 298 K): δ 9.34 (dd, 2H, J_{PH} = 17 Hz, J_{HH} = 7 Hz, *ortho*-H on substituted phenyl), 7.96 (m, 2H, Ar), 7.79 (s, 8H, *o*-C*H* BAr$^F_4$), 7.71 (m, 4H, Ar), 7.67 (s, 4H, *p*-C*H* BAr$^F_4$), 7.56-7.30 (m, 10H, Ar), 7.17 (m, 4H, Ar), 6.80 (br s, 2H, Ar) 4.44 (s, 2H, sp^2 C*H*-NBD), 3.74 (s, 2H, sp^2 C*H*-NBD), 3.33 (m, 4H, sp^3 C*H*-NBD and methine C*H*), 2.91 (sept, 2H, J_{HH} = 6 Hz, methine C*H*), 2.00 (d, 6H J_{HH} = 6 Hz, CH_3), 1.51 (d, 6H, J_{HH} = 6 Hz, CH_3), 1.27 (s, 2H, CH_2 NBD) 1.05 (d, 6H, J_{HH} = 6 Hz, CH_3), 0.21 (d, 6H, J_{HH} = 6 Hz, CH_3).

^{1}H NMR (500 MHz, CD_2Cl_2, 203 K) selected data: δ 9.14 (dd, 2H, J_{PH} = 18 Hz, J_{HH} = 8 Hz, *ortho*-H on substituted phenyl).

ESI-MS (1,2-difluorobenzene): *m/z* [M]$^+$ 901.32 (Calc. 901.3169) with the correct isotope pattern.

Elemental analysis found (calc. for $C_{87}H_{72}BF_{24}OP_2Rh$): C 59.06 (59.20) H 3.98 (4.11).

Structural Data of Complexes 2-R

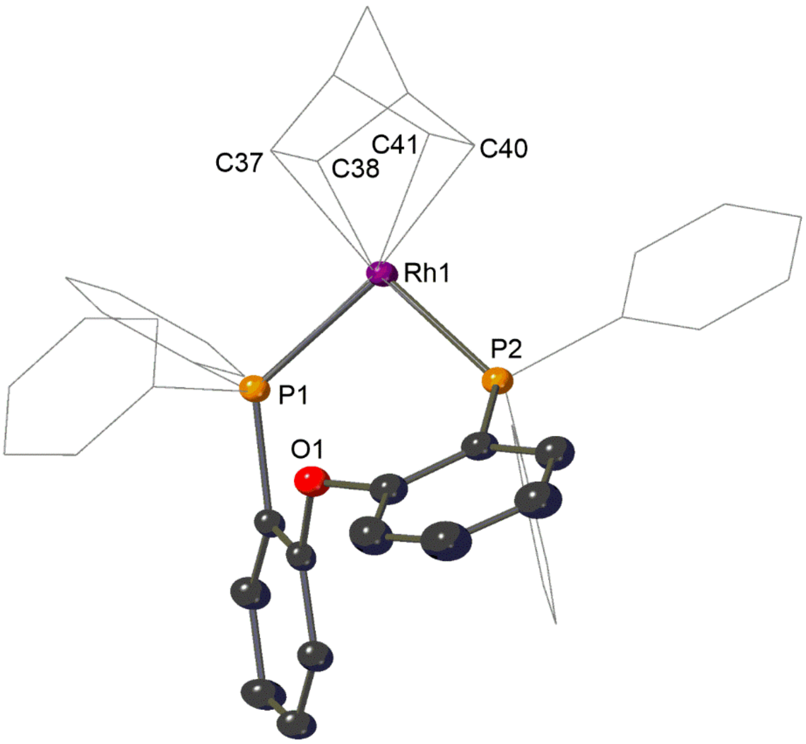

Figure S8. Molecular structure of the cationic portion of **2-H**. Ellipsoids at the 50% probability level. Anion and hydrogen atoms omitted for clarity. The labelling system for the NBD carbons and phosphorus atoms is the same for all four NBD complexes **2-H**, **2-Me**, **2-OMe** and **2-^{i}Pr**.

Table S2. Structural data for NBD complexes **2-H**, **2-Me**, **2-OMe** and **2-^{i}Pr** determined from single crystal x-ray diffraction.

Bond/Angle	2-H	2-Me	2-OMe	2-^{i}Pr
**Rh-P1 (Å)**	2.3392(9)	2.3514(6)	2.3813(7)	2.4482(7)
**Rh-P2 (Å)**	2.3438(7)	2.3949(7)	2.3368(6)	2.4256(5)
**Rh-O (Å)**	3.547(2)	3.5529(16)	3.5545(18)	3.498(8)
**Rh-C37 (Å)**	2.213(3)	2.219(3)	2.200(2)	2.1777(17)
**Rh-C38 (Å)**	2.179(3)	2.236(3)	2.210(3)	2.1825(16)
**Rh-C40 (Å)**	2.184(3)	2.164(3)	2.160(3)	2.184(2)
**Rh-C41 (Å)**	2.215(3)	2.164(3)	2.160(3)	2.178(3)
**P1-Rh-P2 (°)**	98.58(3)	100.79(2)	100.67(2)	103.91(2)

Variable temperature solution NMR spectroscopy of 2-R

2-H

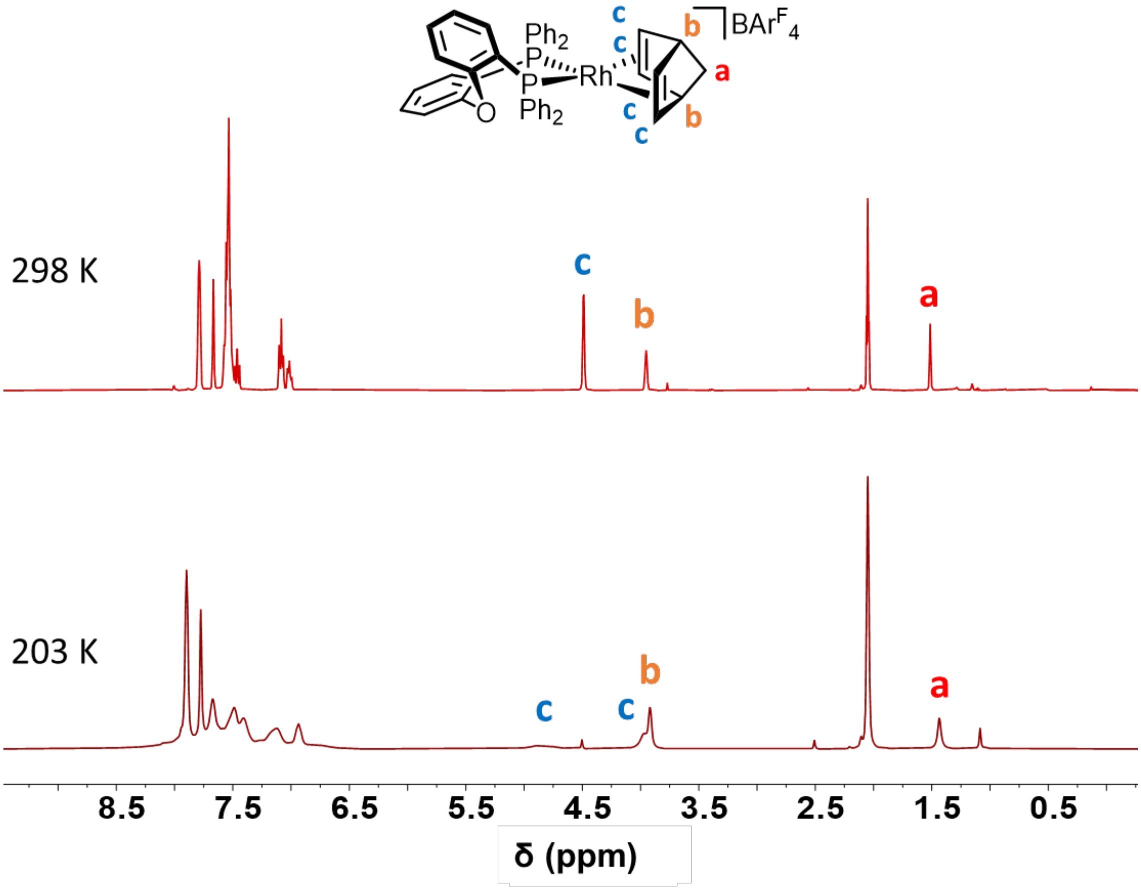

Figure S9. ^{1}H NMR of **2-H** at 298 K and 203 K (acetone-D_6, 400 MHz).

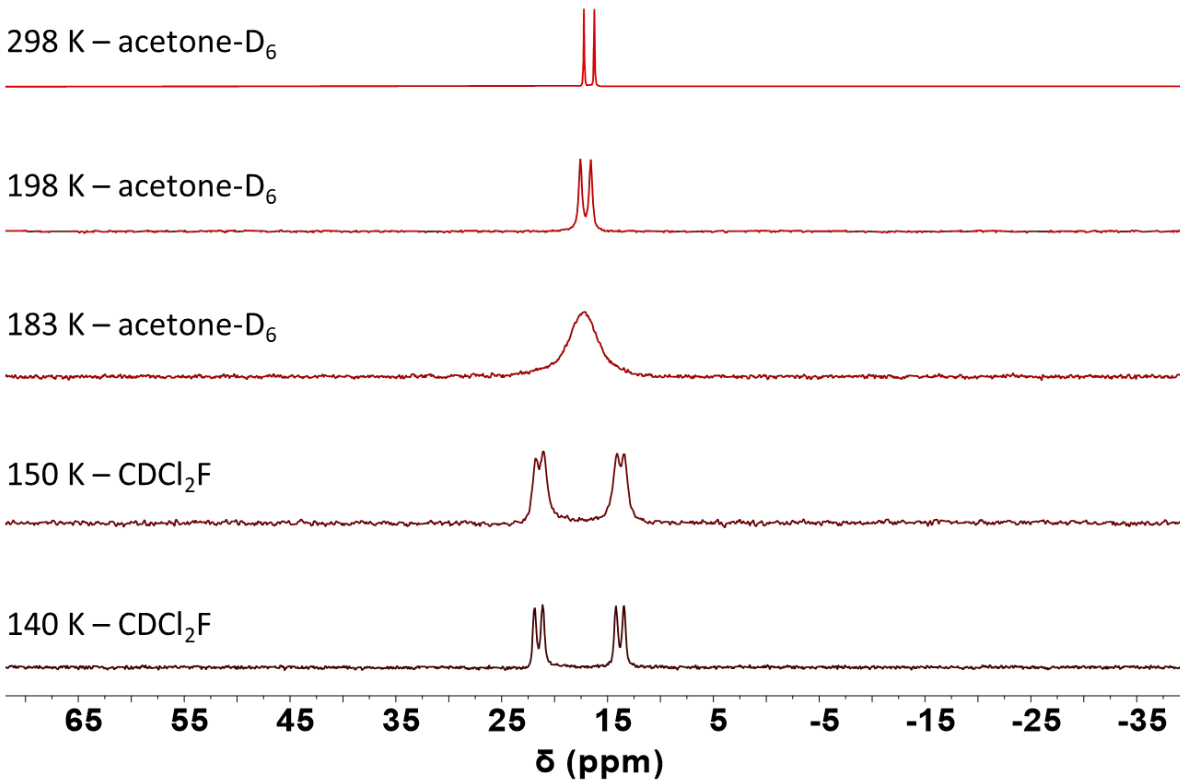

Figure S10. Variable temperature $^{31}P\{^1H\}$ NMR of **2-H** (202 MHz).

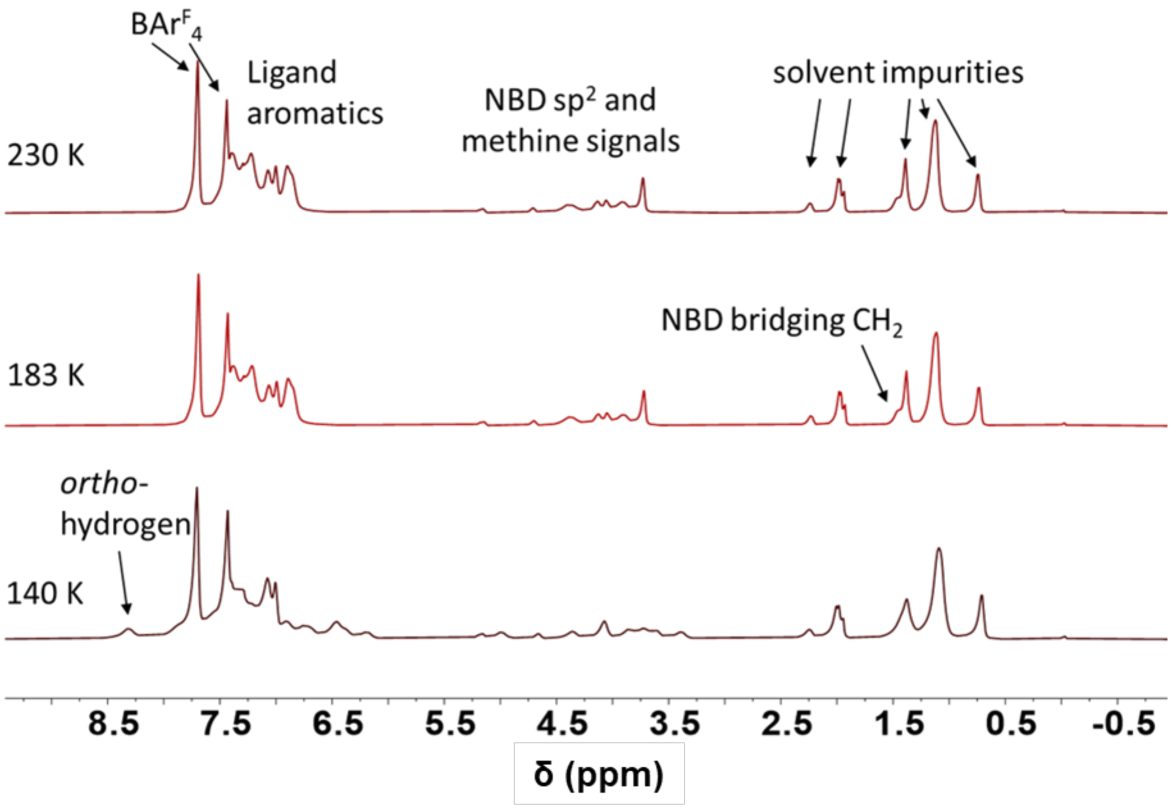

Figure S11. Variable temperature ^1H NMR spectra of **2-H** ($CDCl_2F$, 500 MHz). The solvent impurities arise from the synthesis of the Freon solvent, which proved difficult to purify.

2-Me

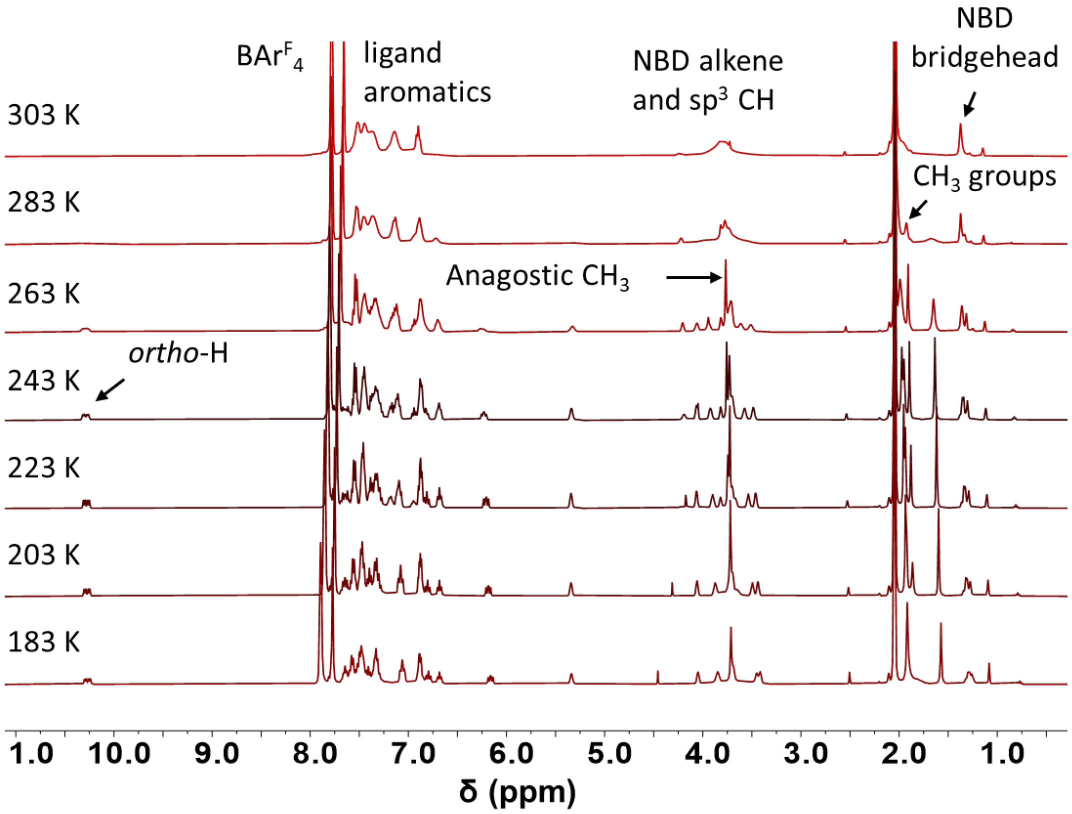

Figure S12. Variable temperature ^{1}H NMR spectra of **2-Me** (acetone-D$_6$, 400 MHz).

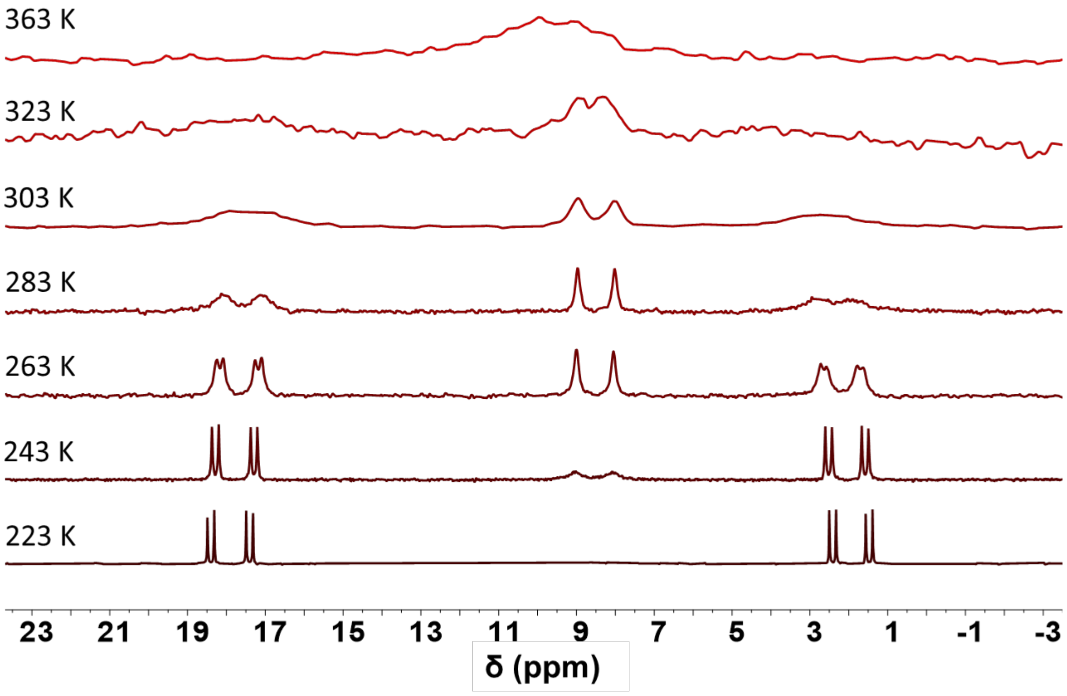

Figure S13. Variable temperature ^{31}P{^{1}H} NMR spectra of **2-Me** (acetone-D$_6$ except 363 K which is in 1,2-dichloroethane, 162 MHz).

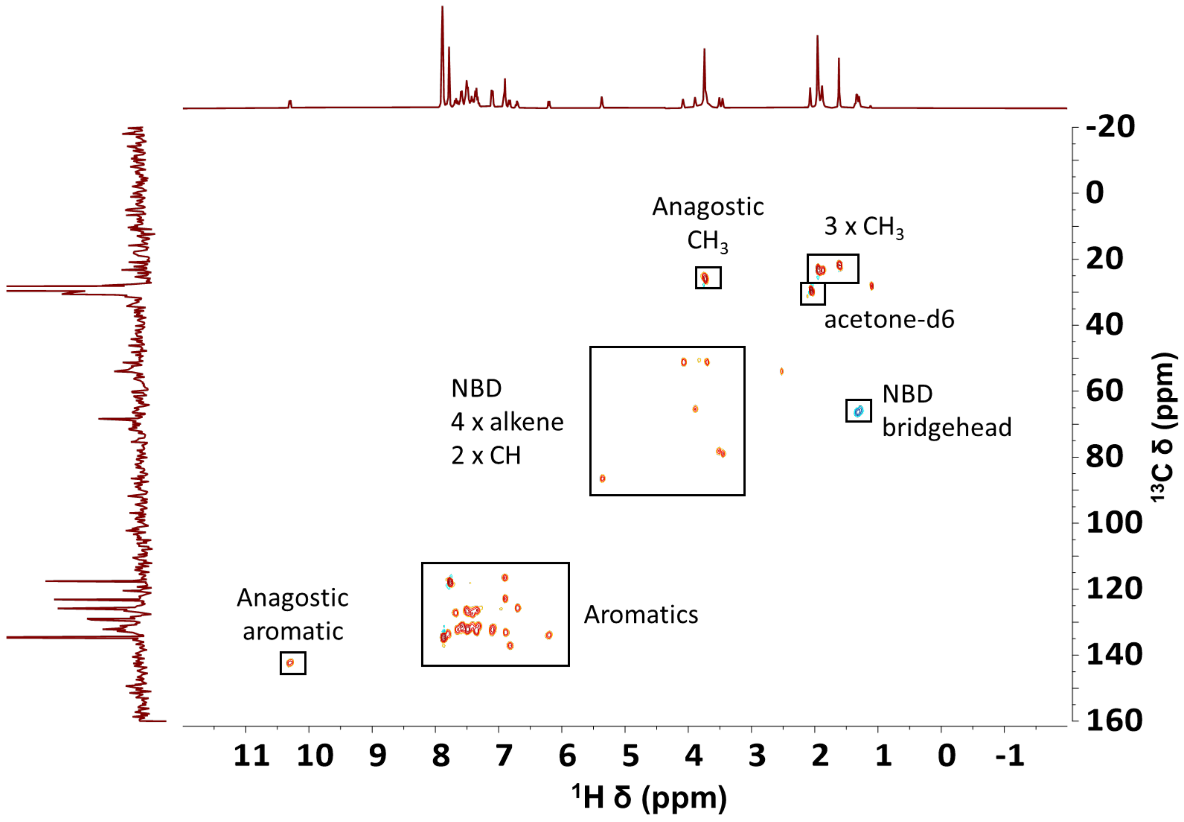

Figure S14. HSQC spectrum of **2-Me** (acetone-D_6, 400/101 MHz, 203 K).

2-OMe

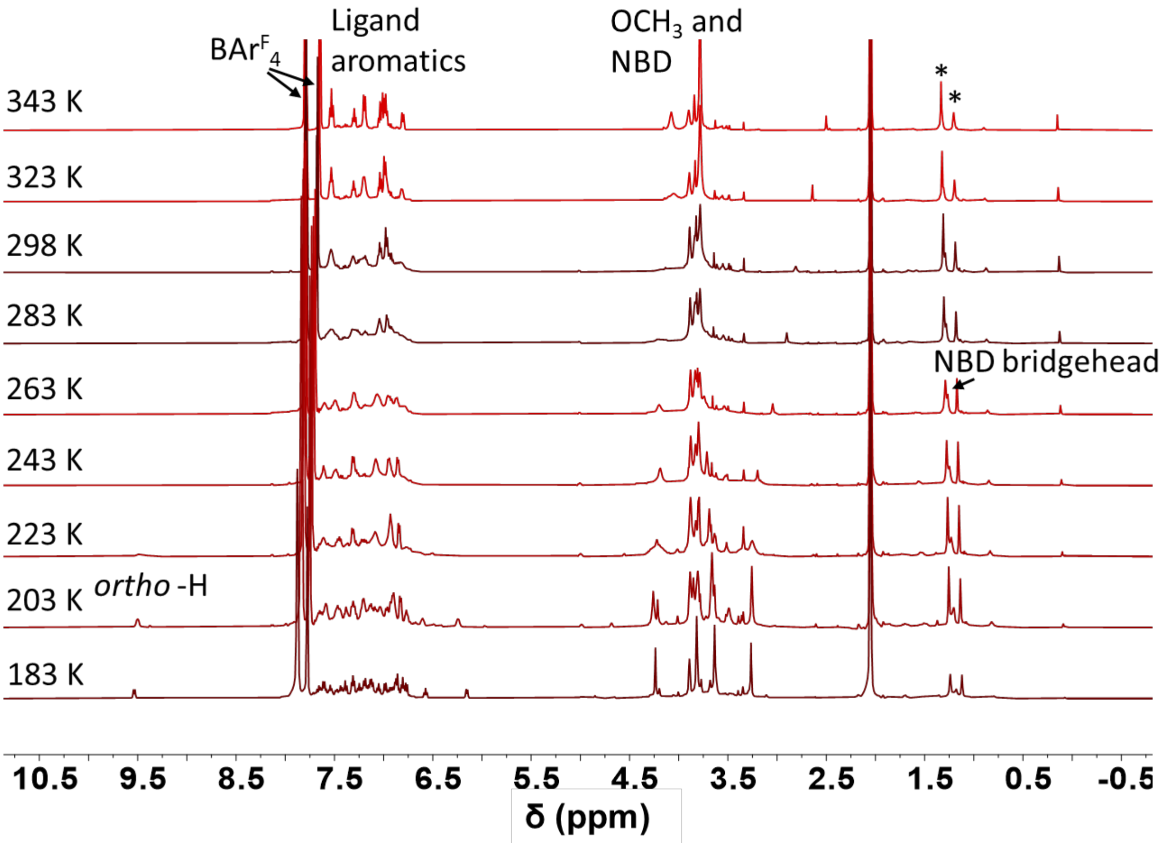

Figure S15. Variable temperature $^{1}H\{^{31}P\}$ NMR spectrum of **2-OMe** (acetone-D_6, 500 MHz). * denotes an unknown minor impurity persistent after recrystallisation.

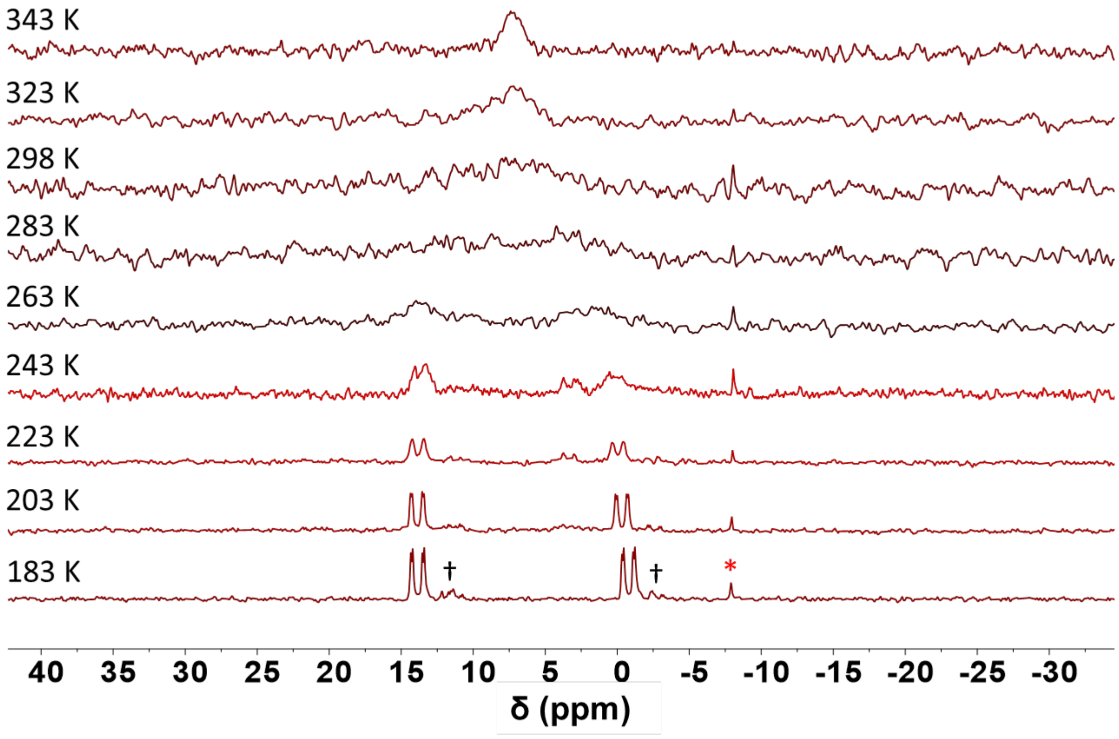

Figure S16. Variable temperature $^{31}P\{^1H\}$ NMR spectrum of **2-OMe** (acetone-D_6, 202 MHz). † Highlights minor isomer of **2-OMe** from alternative anagostic arrangement. * Denotes an unknown impurity of less than 3% persistent after recrystallisation.

2-^{i}Pr

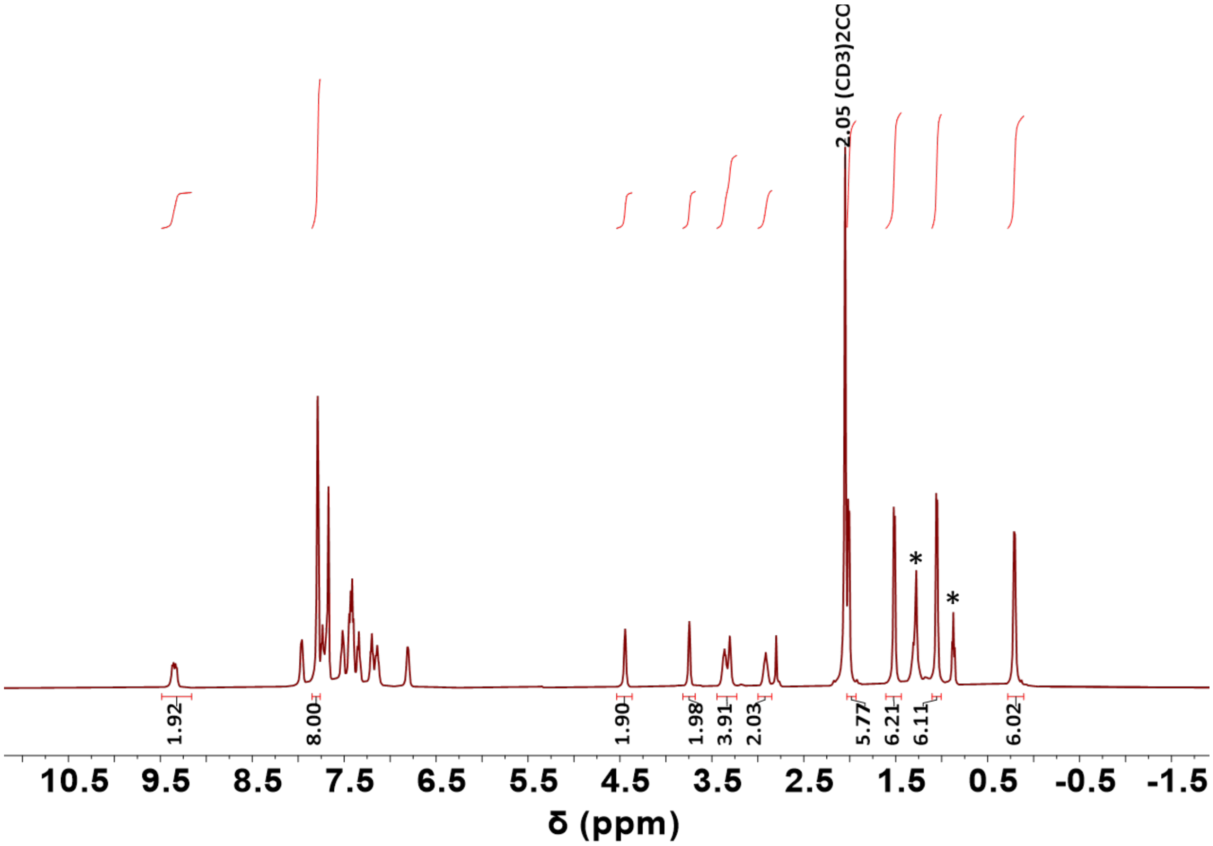

Figure S17. ^{1}H NMR spectrum of **2-^{i}Pr** that shows the C_2 symmetry in the complex (acetone-D_6, 500 MHz, 298 K). Peaks marked with * are residual pentane persistent after recrystallisation and the bridging NBD CH_2 (1.27 ppm).

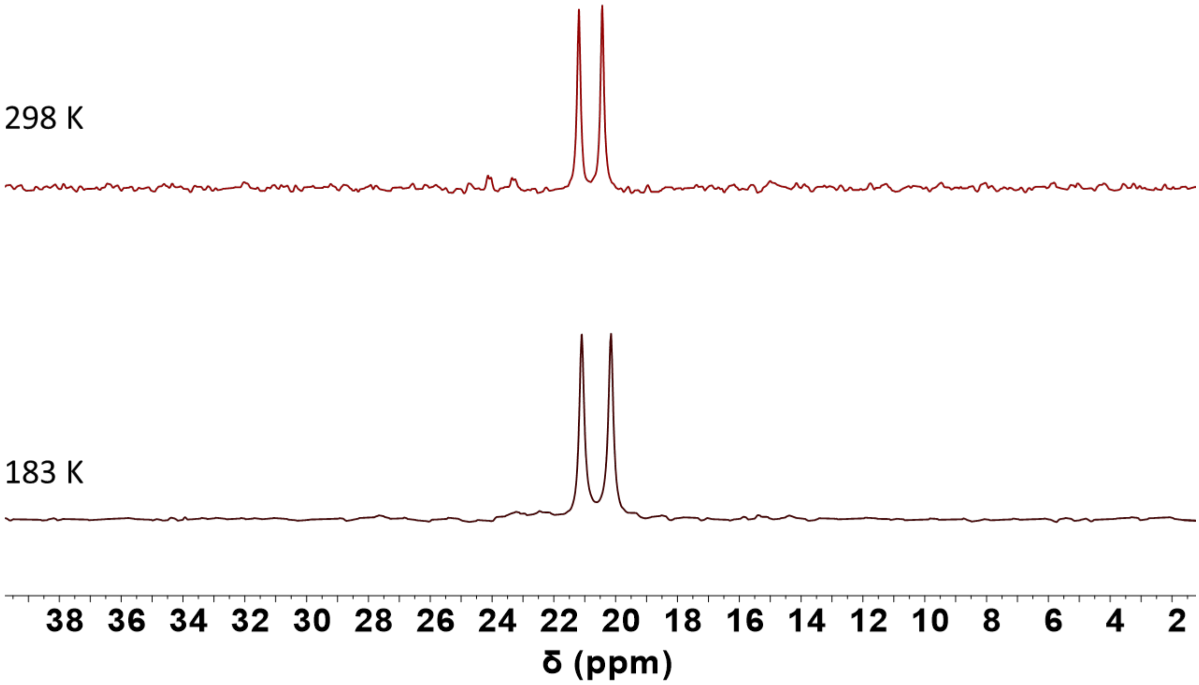

Figure S18. $^{31}P\{^1H\}$ NMR spectra of **2-^{i}Pr** at 298 K and 183 K to show there is no observable fluxional process occurring on the NMR timescale (acetone-D_6, 202 MHz).

Modelling of fluxional behaviour in 2-Me and 2-OMe

All modelling was completed by Alex Heyam at the University of York.

Methods

Spectra were first processed in Topspin, and then fitted using Spinach[6] together with Matlab's internal optimisation routines. Initially a model without exchange was fitted, to estimate chemical shifts, peak widths and populations. The data was then refitted using models which included chemical exchange. Chemical shifts and R2 were held constant, while exchange rates could vary. Initial values for the fitting were chosen partly based on the non-exchange fitting, partly by trial and error. For **2-Me**, models with and without direct exchange between the asymmetric states were tested. For **2-OMe**, only a simple two state exchange model was tested, but the chemical shift of A2 was also allowed to vary.

There are several limitations to the fitted values. Firstly, power gated decoupling was used in the 1D experiments, which could affect peak intensity. We believe that all peaks should be affected similarly, and so this should not have a large effect on the results. Secondly, there were no non-exchanging peaks to use as internal references for linewidth. Instead, modelled R2 values were chosen based on the narrowest linewidth observed at any temperature (approximately 8 Hz) and assumed to remain roughly constant at all temperatures. This introduces an error on the order of 10 Hz into all modelled exchange rates. Finally, there was no internal temperature standard, so there may be an unknown systematic error in the reported temperatures.

When modelling the chemical shifts of **3-Me**, peak widths and populations were fitted for each temperature firstly without exchange modelling and then with two exchange processes; direct exchange of two equivalent asymmetric species $\mathbf{A_1A_2}$ and $\mathbf{A_2A_1}$, observed as the two ^{31}P NMR signals **A**, and exchange between **A** and **B**. Although the conversion between the two **A** species is expected to go via a ring flip, which a route via **B** would provide, this would be 2-10 times slower than via a different route with unknown transition states. The model was initially fitted without direct exchange between $\mathbf{A_1A_2}$ and $\mathbf{A_2A_1}$ (only via **B**) but this did not reproduce the data well. At lower temperatures, the **A**↔**B** equilibrium favours the **A** state ($k_{AB} \approx 6k_{BA}$ at 243 K) and increasing the temperature pushed the equilibrium further towards the **B** state ($k_{AB} \approx 2k_{BA}$ at 303 K). Increasing the temperature further, they all coalesce toward one specie.

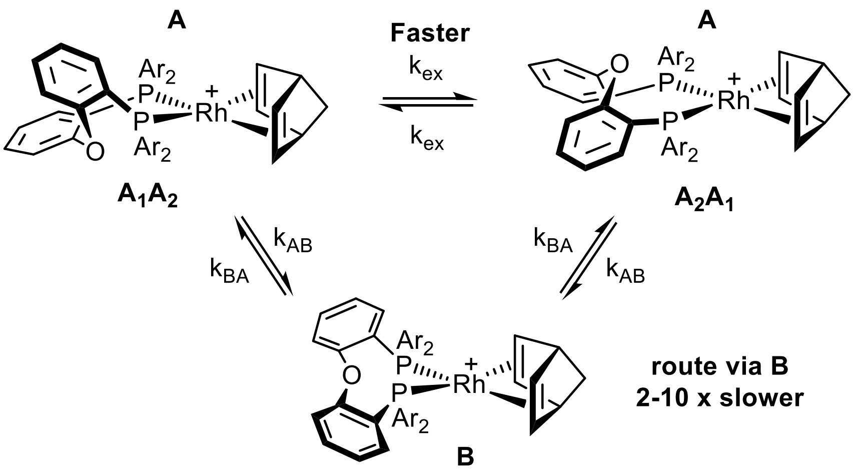

Scheme S2. Proposed fluxional process occurring in complexes **2-Me** and **2-OMe**.

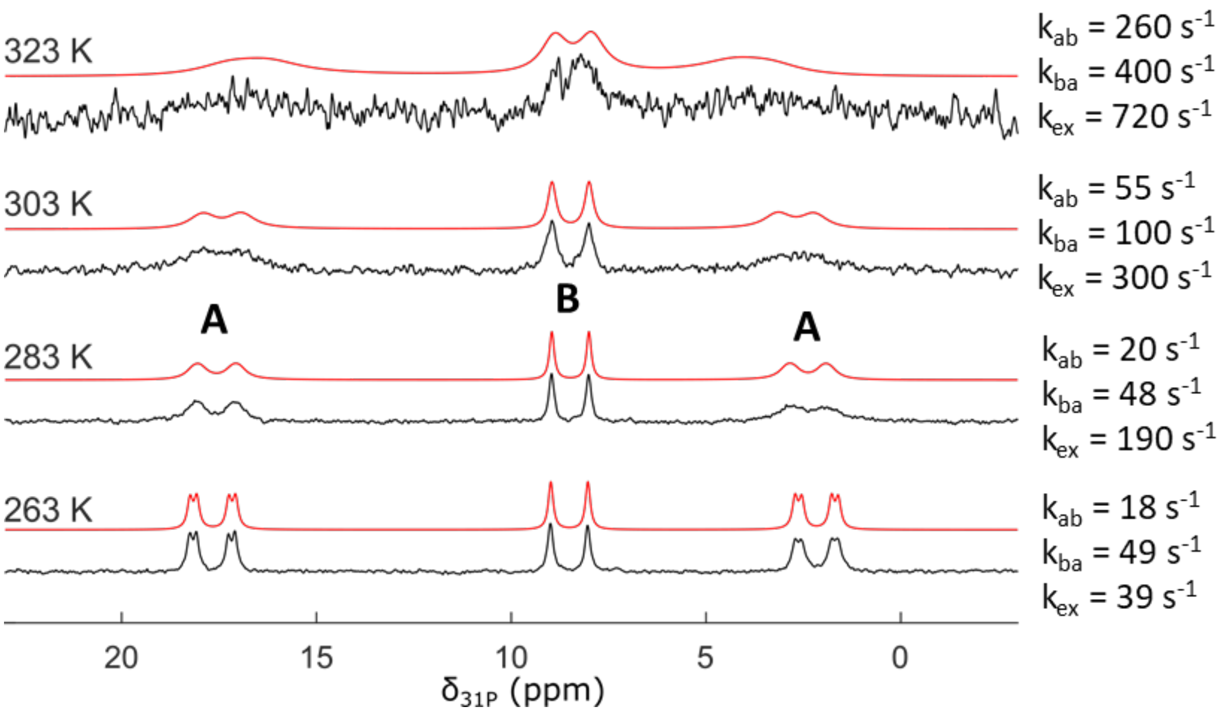

Figure S19. Modelled (red) and real (black) $^{31}P\{^1H\}$ NMR data of **2-Me** at different temperatures (acetone-D_6, 162 MHz). The rates are calculated estimates of the exchange between two asymmetric **A** states (kex), and the rate of exchange between the asymmetric states and **B** (k_{ab} and k_{ba}).

The data for **2-OMe** was modelled but the population of the **B** state was too low to include, therefore, a simple line-fitting was conducted which showed the asymmetric exchange to vary in rate from k_{ex} = 44 s^{-1} at 183 K to 26,000 s^{-1} at 323 K (Figure S20), much faster than the more bulky **2-Me** (k_{ex} = 720 s^{-1} at 323 K).

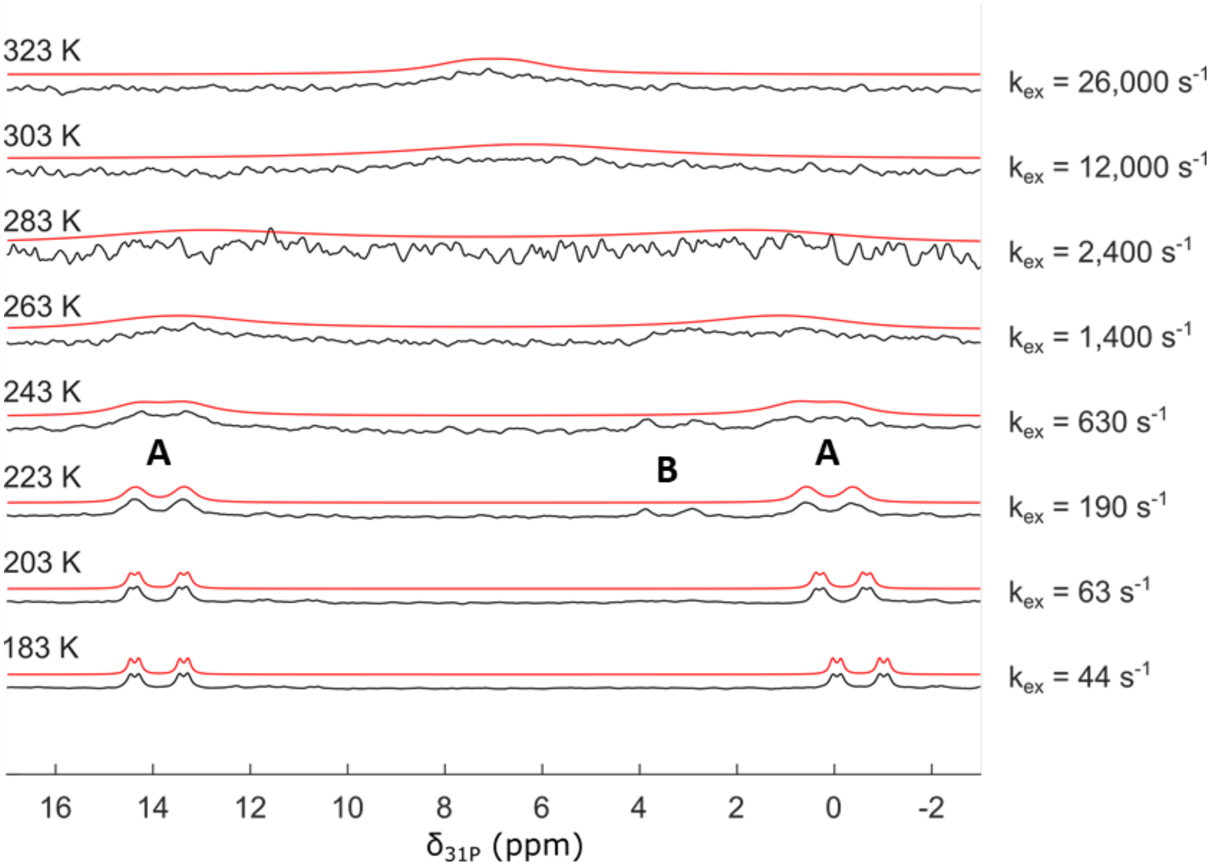

Figure S20. Modelled (red) and real (black) $^{31}P\{^{1}H\}$ NMR data of **2-OMe** at different temperatures (acetone-D_6, 162 MHz). The rates are calculated estimates of the exchange between two asymmetric **A** states. The symmetric **B** state is not in high enough concentration to include in equilibria modelling.

Identification of Anagostic Motifs

Aryl anagostic coupling constants

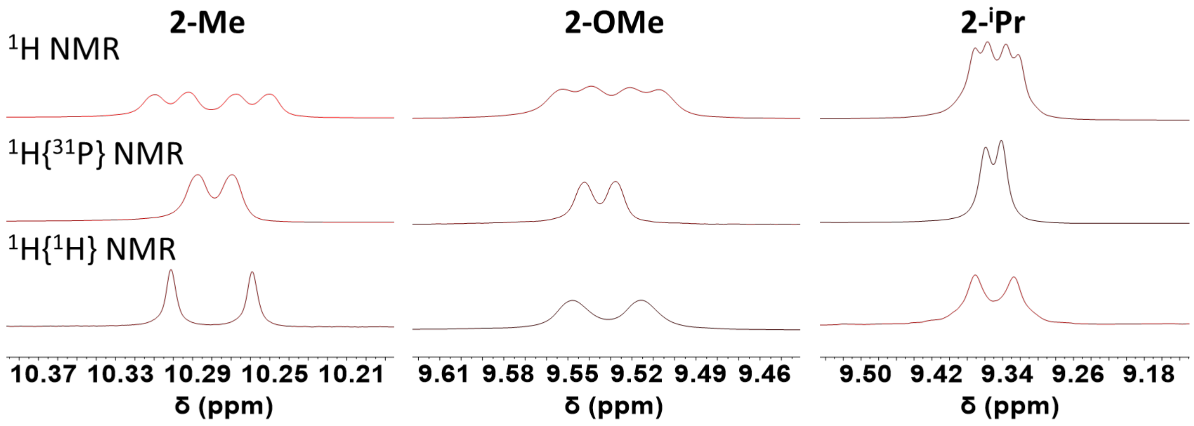

Figure S21. A selection of the ^{1}H (top), ^{1}H{^{31}P} (middle), and ^{1}H{^{1}H} (bottom) NMR spectra of **2-Me**, **2-OMe** and **2-^{i}Pr** (acetone-D$_6$, 400 MHz, 203 K) that shows that the downfield shifted signal comprises J_{PH} (17, 16 and 17 Hz, respectively) and J_{HH} (7, 6 and 7 Hz, respectively). ^{1}H{^{1}H} experiments have the decoupler centred at 7.67, 7.23 and 7.21 ppm, respectively.

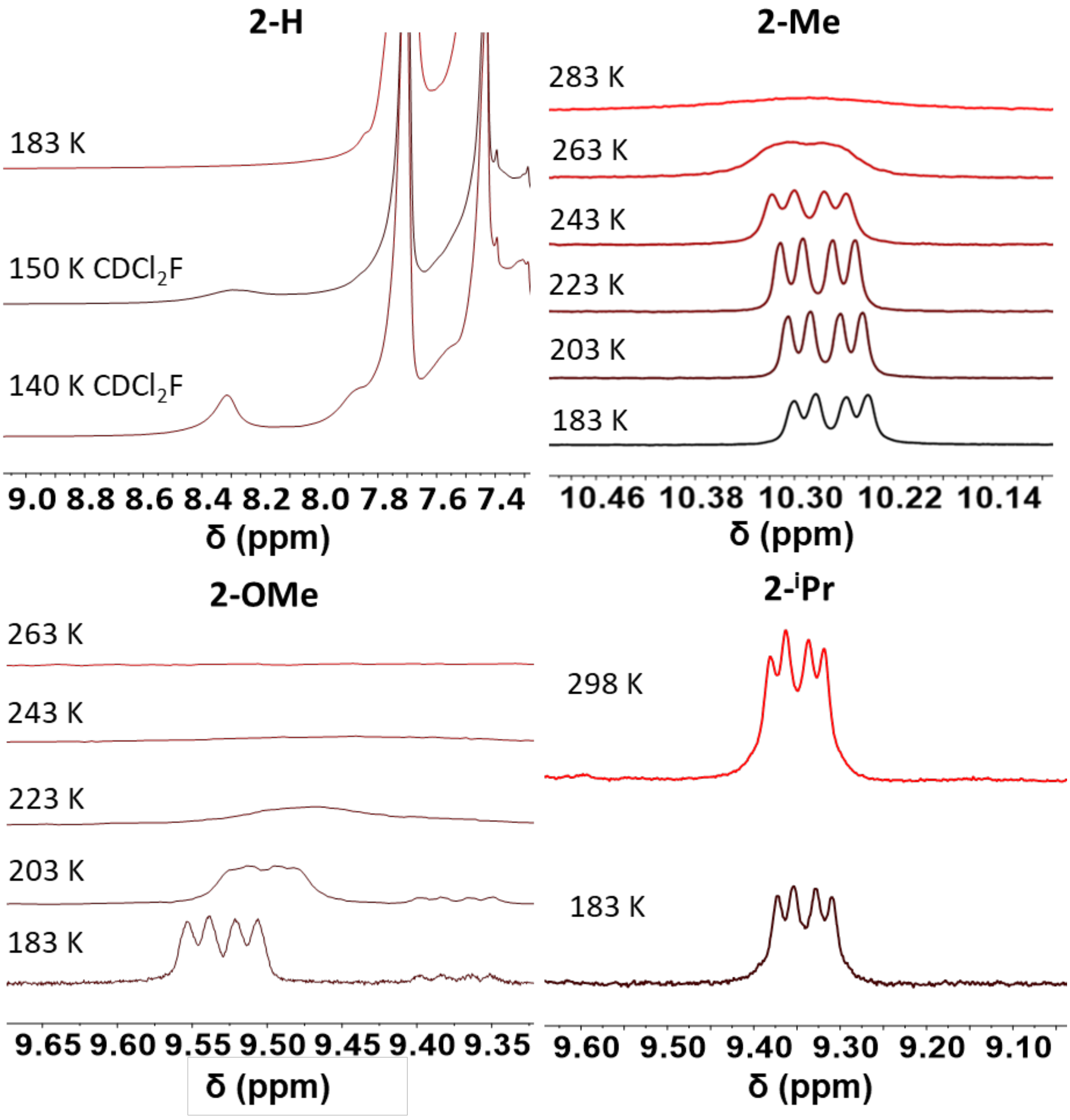

Figure S22. Variable temperature ^{1}H NMR spectrum of the downfield shifted *ortho*-H in **2-R** (acetone-D_6 unless specified, 400 MHz).

Assignment of *ortho*-hydrogen atoms in free ligands 1-R

The numbering system in the following assignments is different from the main paper. This is to make it easier to follow the logical and sequential assignments below. In all four cases the *ortho*-proton on the non-backbone aromatics is the focus of the assignment.

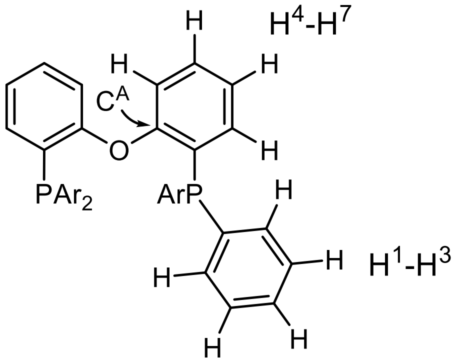

DPEphos-H **1-H**

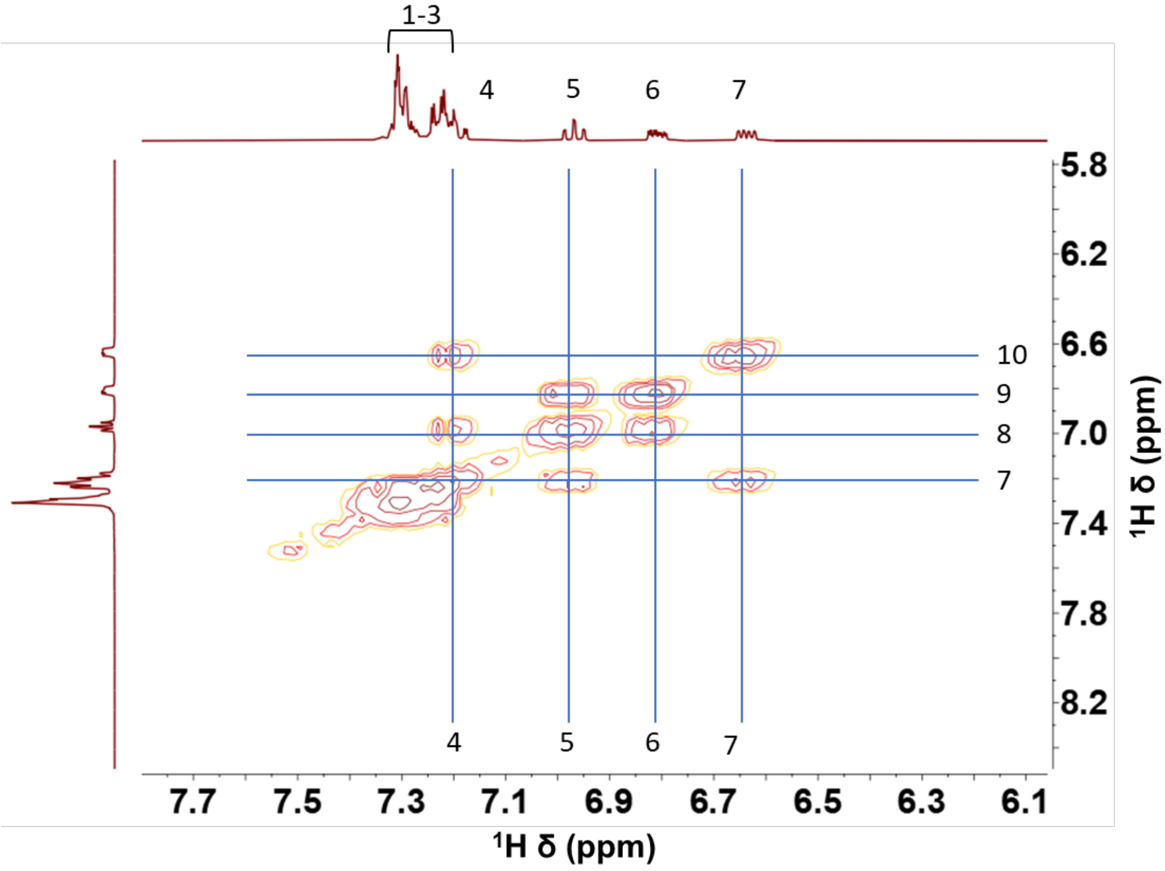

Figure S23. Aromatic section of the COSY NMR spectrum of **1-H** showing that protons 7-10 interact with one another and therefore most likely are bonded to the same aromatic ring (CD_2Cl_2, 298 K, 400/400 MHz).

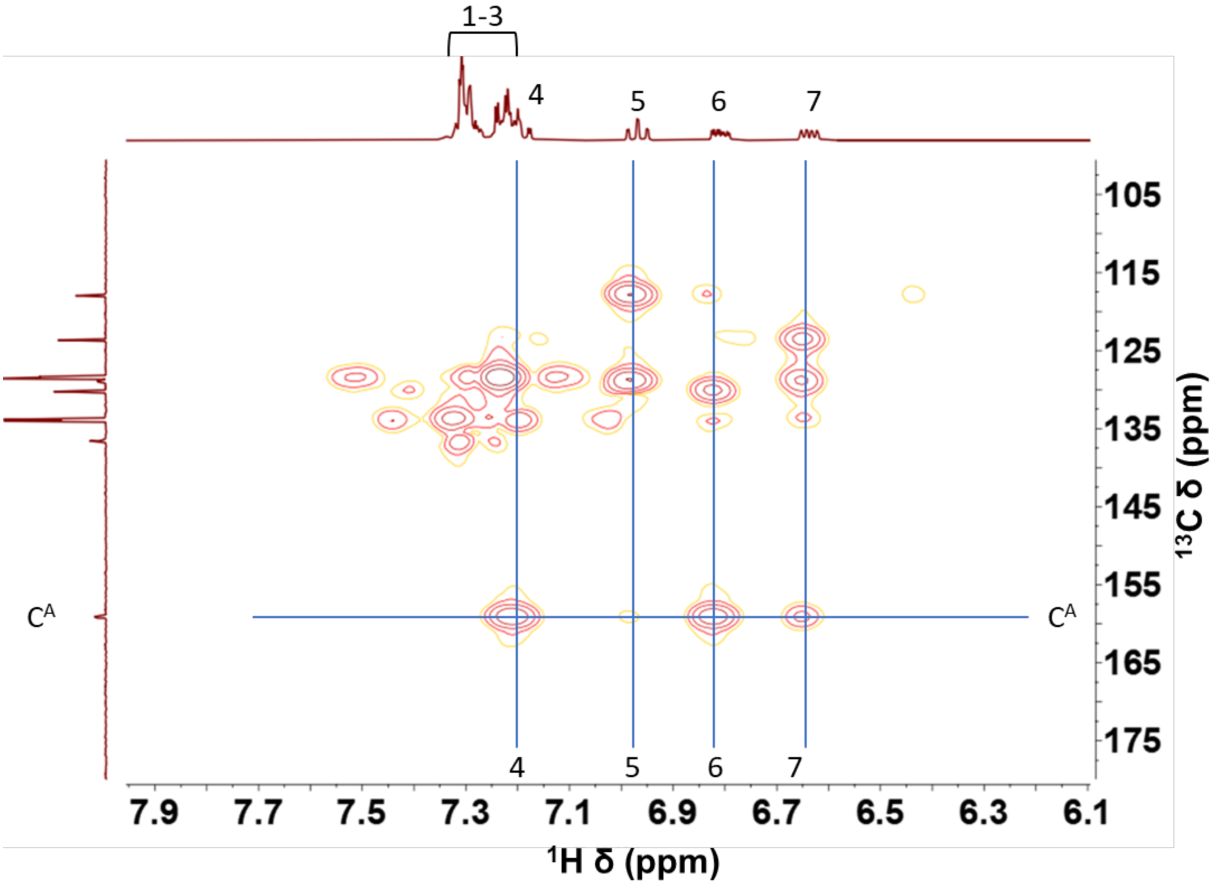

Figure S24. Aromatic section of the HMBC NMR spectrum of **1-H** showing the interactions of C^A with protons 4-7 (CD_2Cl_2, 400/101 MHz, 298 K).

As protons H^4-H^7 appear to interact via COSY NMR they are very likely to be bonded to the same aromatic ring. The most downfield carbon signal is C1 at 159.5 ppm which is likely to be the carbon bound to the oxygen. Considering C^A interacts with protons H^4, H^6 and H^7 via HMBC then protons H^4-H^7 are very probably on the aromatic ring coordinated to the oxygen and the other ring contains protons H^1-H^3 that produce the signal from 7.33-7.21 ppm. H^1-H^3 all appear to couple via COSY as well. Therefore, the *ortho*-protons that show anagostic characteristics in **2-H** most likely give rise to signals from 7.33-7.21 ppm but they cannot be unambiguously assigned due to signal overlap.

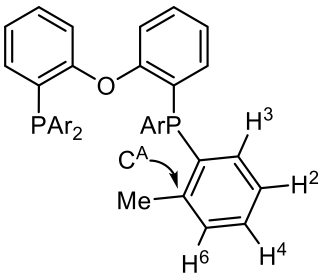

DPEphos-Me **1-Me**

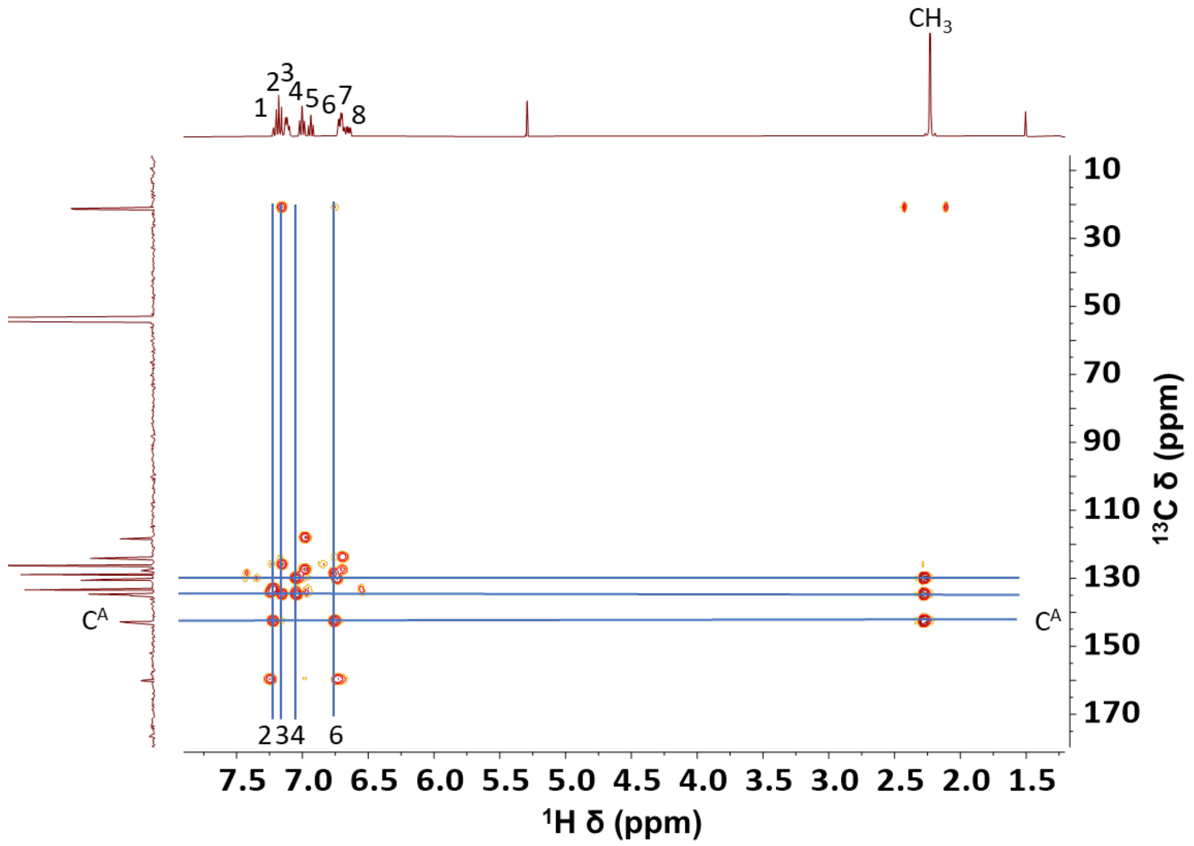

Figure S25. HMBC NMR spectrum of ligand **1-Me** showing that the carbon atoms that interact with the methyl CH_3 also interact with aromatic protons 2, 3, 4 and 6 (CD_2Cl_2, 400/101 MHz, 295 K).

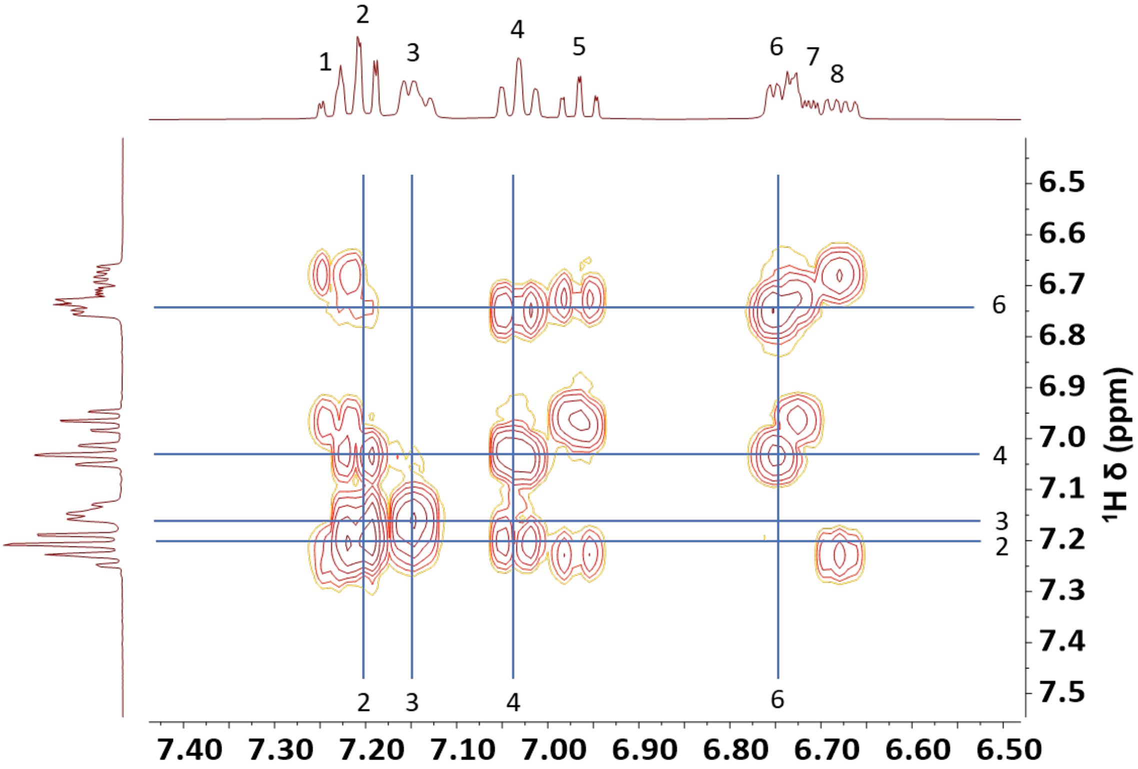

Figure S26. COSY NMR spectrum of **1-Me** showing that protons 2, 3, 4 and 6 all interact through COSY interactions (CD_2Cl_2, 500/500 MHz, 295 K).

In the HMBC the methyl group couples with three carbons and the four protons that couple with those three carbons in the HMBC are H^2, H^3, H^4 and H^6. H^2 and H^4 both couple to two different protons in the COSY NMR spectrum therefore must be in the central positions. H^6 and H^3 have only one. C^A has HMBC interactions with H^2 and H^6, as H^6 has only one COSY signal then the proton adjacent to the methyl must be H^6 and then sequentially we can assign the other protons with the COSY NMR.

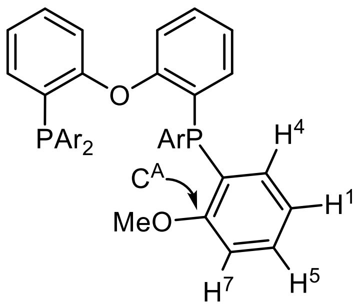

DPEphos-OMe **1-OMe**

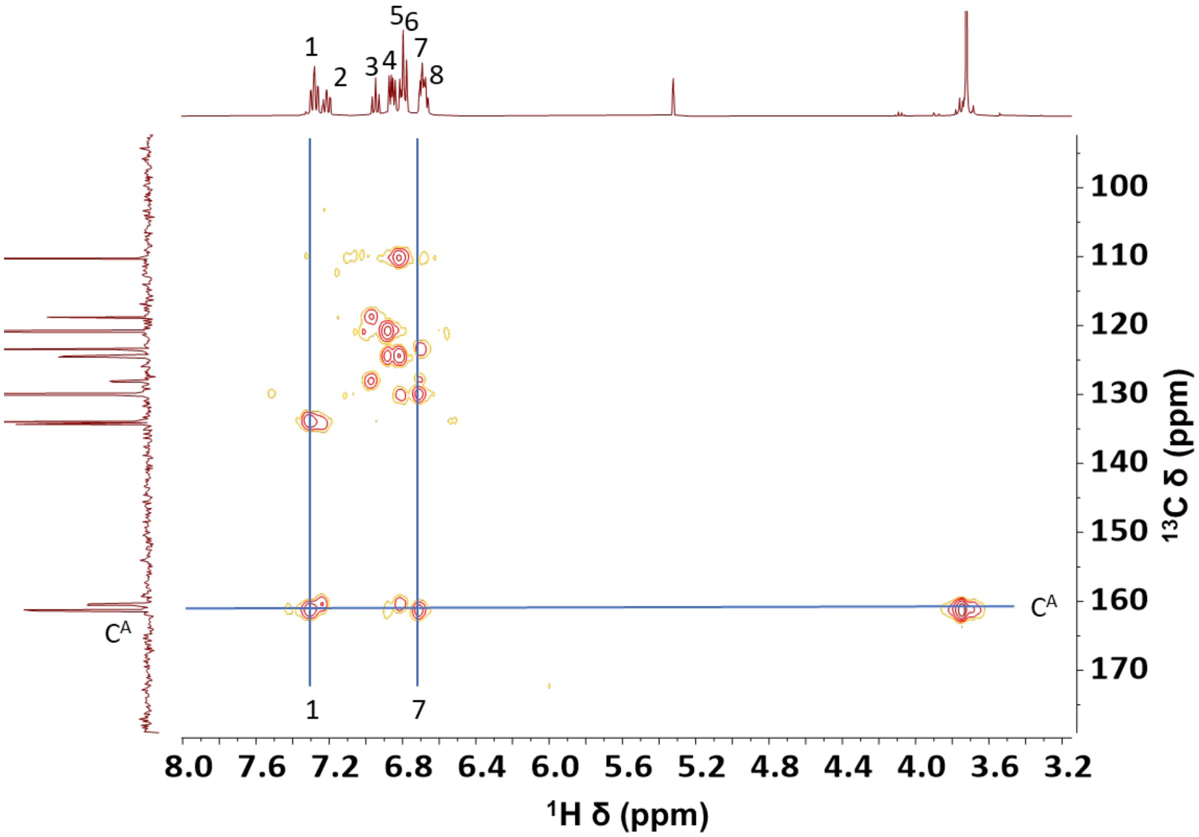

Figure S27. HMBC NMR spectrum of ligand **1-OMe** showing that the same carbon has interactions with the OCH_3 and protons 1 and 7 (CD_2Cl_2, 400/101 MHz, 295 K).

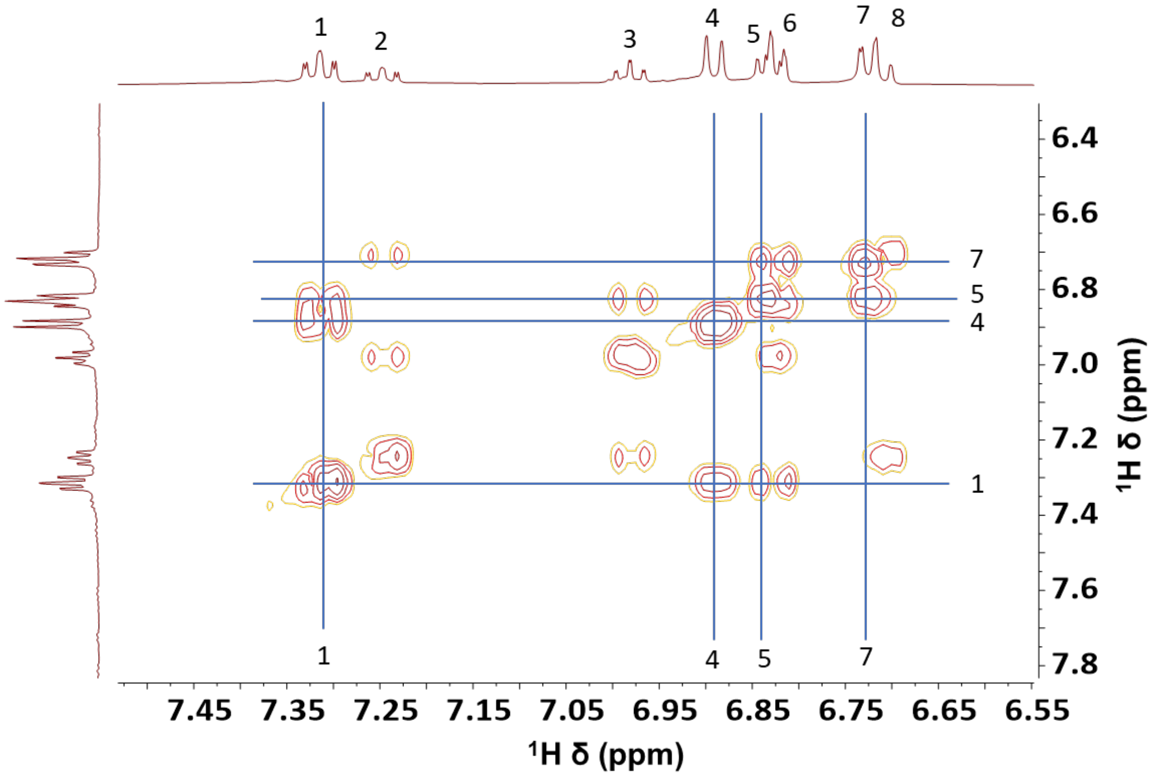

Figure S28. COSY NMR spectrum of **1-OMe** showing that protons 1, 4, 5 and 7 all interact through COSY interactions and therefore very likely to be the four protons in the substituted aromatic ring (CD_2Cl_2, 500/500 MHz, 295 K).

The methoxy proton interacts only with one carbon (C^A) via HMBC and C^A has two other interactions with H^1 and H^7. H^1 interacts via COSY with H^4 and H^5 and H^7 interacts via COSY with only H^5 then H^7 must be the proton adjacent to the methoxy group and the COSY NMR sequentially assigns the *ortho*-proton in the free ligand as H^4.

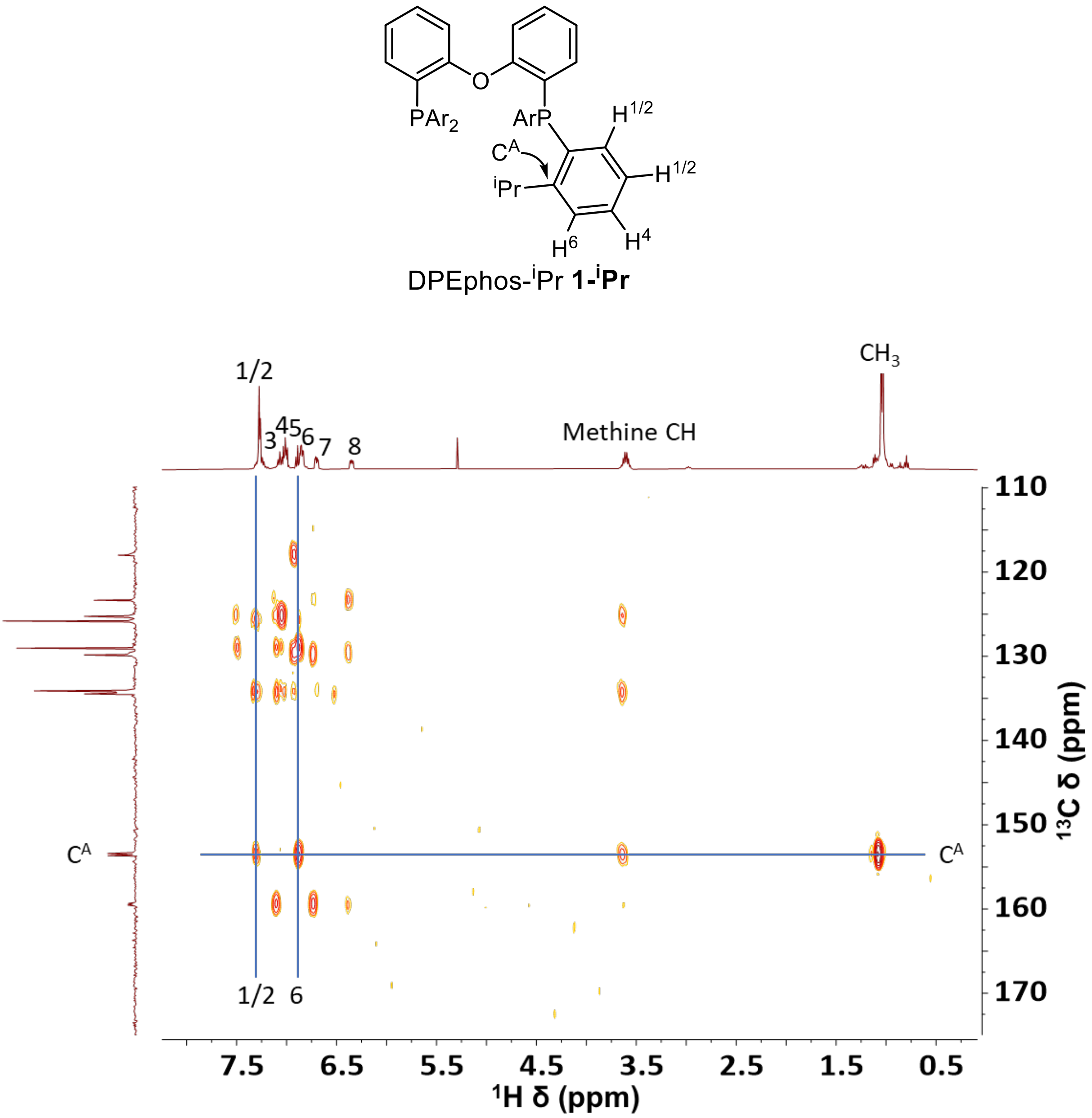

Figure S29. HMBC NMR spectrum of ligand **1-ⁱPr** showing that the carbon atoms that interact with the isopropyl CH_3 also interact with aromatic protons 6 and either 1 or 2 (these two cannot be differentiated) (CD_2Cl_2, 400/101 MHz, 295 K).

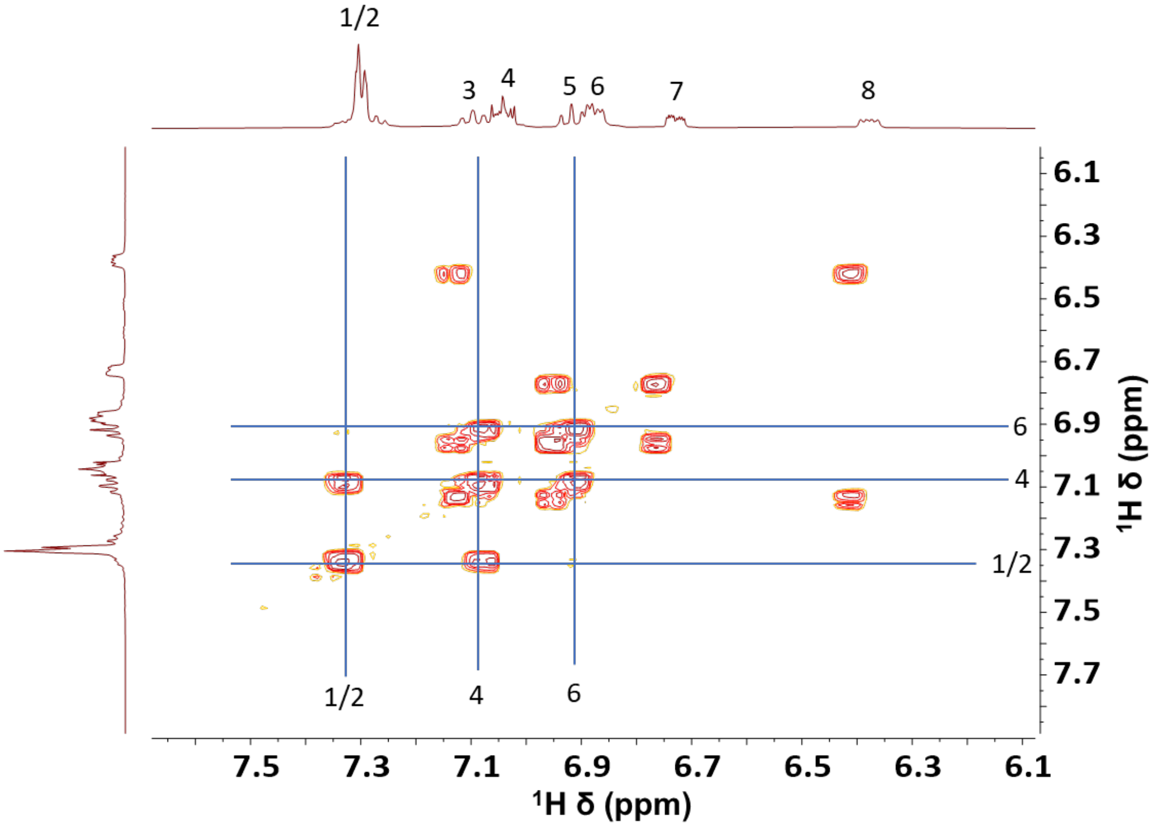

Figure S30. COSY NMR spectrum of **1-^{i}Pr** showing that protons 1, 2, 4 and 6 interact with one another and therefore most likely are bonded to the same aromatic ring (CD_2Cl_2, 298 K, 400/400 MHz).

It is clear from the HMBC that the isopropyl CH_3 protons interact only with C^A, which also interacts with the methine C*H* and protons H^6 and either H^1 or H^2 (H^1 and H^2 cannot be differentiated). The COSY NMR spectrum indicates that protons H^1, H^2, H^4 and H^6 are very likely all bound to the same aromatic ring with C^A and the *iso*-propyl group. The strong HMBC signal between C^A and H^6 likely suggests they are adjacent. H^4 is therefore then adjacent to H^6 and either H^1 or H^2. Although H^1 and H^2 cannot be differentiated, the respective chemical shifts are 7.29 and 7.30 ppm and therefore it is not essential to individually assign them to determine a rough chemical shift change in the proton signal in **1-^{i}Pr** and **2-^{i}Pr**.

Difference in chemical shift in free ligand and in anagostic motifs

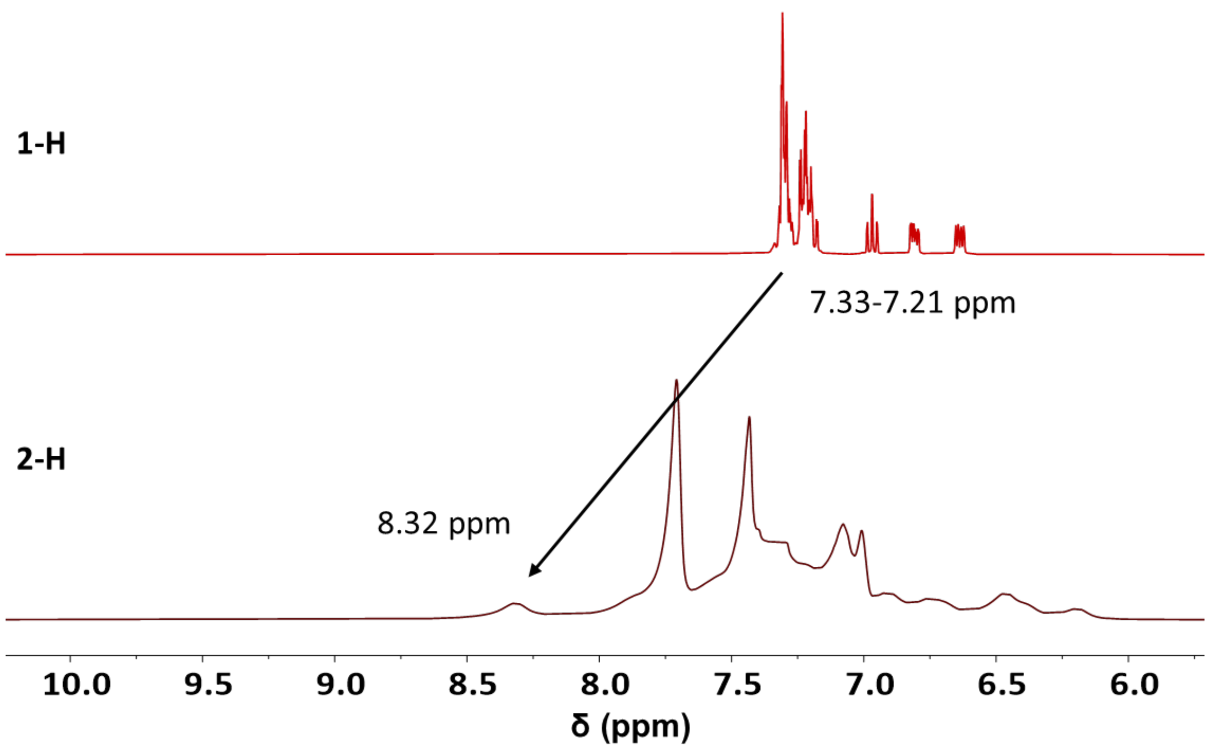

Figure S31. ^{1}H NMR spectrum of free ligand **1-H** (CD_2Cl_2, 500 MHz, 295 K) and coordinated **2-H** ($CDCl_2F$, 500 MHz, 140 K). The difference in chemical shift of the proton that experiences the anagostic motif in the free ligand and in the complex is 0.99-1.11 ppm.

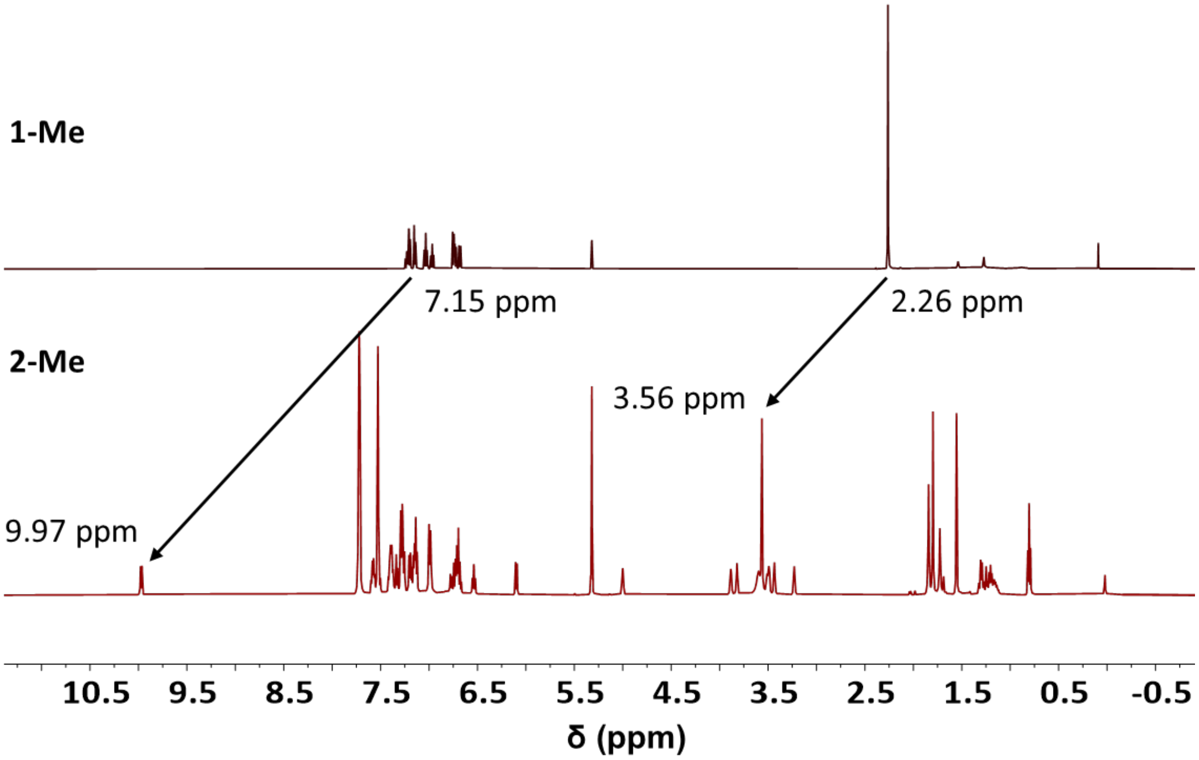

Figure S32. $^{1}H\{^{31}P\}$ NMR spectra of free ligand **1-Me** (295 K) and complex **2-Me** (203 K) (CD_2Cl_2, 500 MHz). The difference in chemical shift of the protons the are suspected to experience anagostic motifs are shown as 2.82 (aryl) and 1.30 (alkyl) ppm.

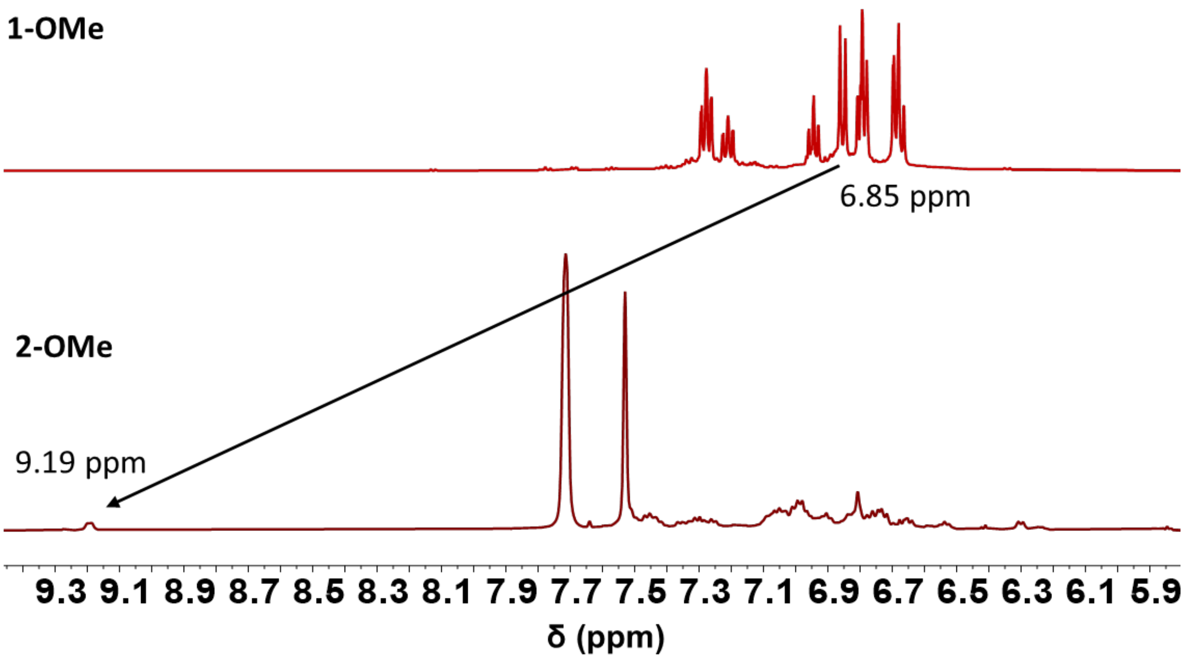

Figure S33. $^1H\{^{31}P\}$ NMR spectra of free ligand **1-OMe** (295 K) and coordinated **2-OMe** (203 K) (CD_2Cl_2, 400 MHz). The difference in chemical shift of the proton that experiences the anagostic motif in the free ligand and in the complex is 2.34 ppm.

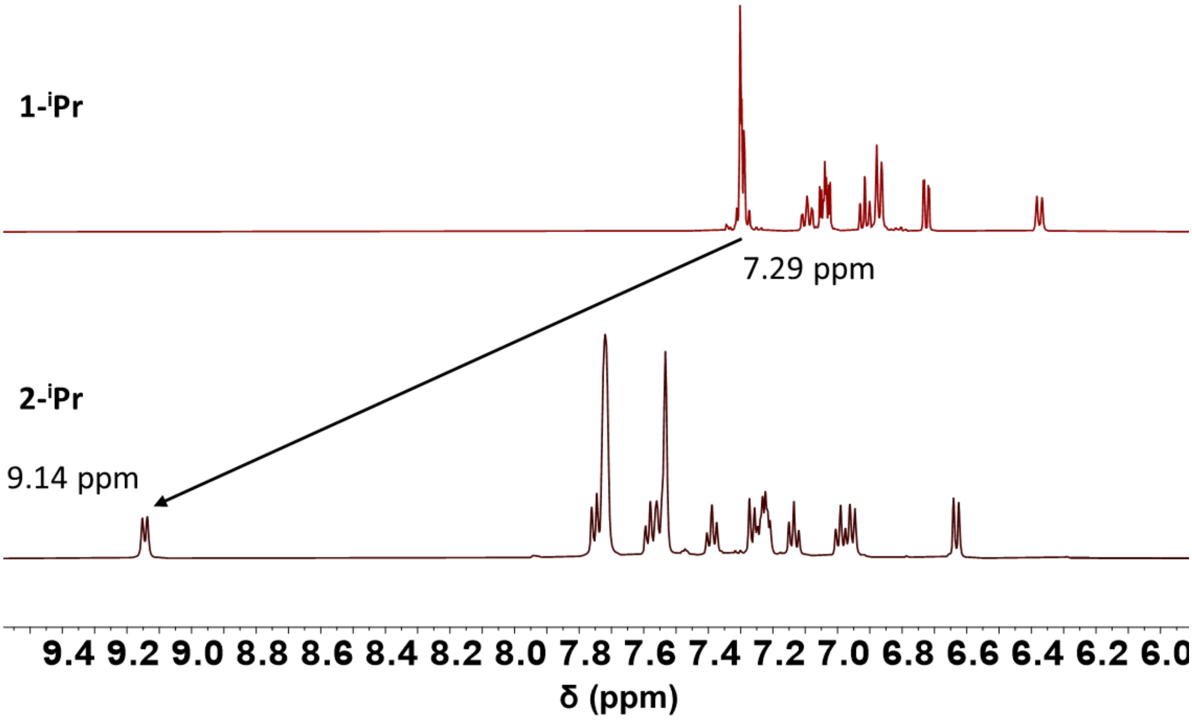

Figure S34. $^1H\{^{31}P\}$ NMR spectra of free ligand **1-^{i}Pr** (295 K) and coordinated **2-^{i}Pr** (203 K) (CD_2Cl_2, 400 MHz). The difference in chemical shift of the proton that experiences the anagostic motif in the free ligand and in the complex is 1.85 ppm.

Computational Studies: Structures, Chemical Shifts and Bonding

Computational Details

Molecular calculations employed the GAUSSIAN 16 program package[1] and geometry optimisations were performed with the BP86 GGA functional.[2-3] Stuttgart-Dresden (SDD)[4] relativistic effective core potentials (ECP) in combination with the associated basis sets were utilized to describe Rh and P, with polarization functions added for P (ζ = 0.387).[5] The 6-31G(d,p) basis sets[6-7] were used on remaining atoms. Stationary points were characterized with analytical frequency calculations. Free energies for structures used in the NMR studies included corrections for dispersion, using the D3BJ[8] method, and solvation in acetone using PCM.[9] These were then combined with the thermochemical corrections from the BP86 frequency calculation.

Electronic structure analyses were performed on the geometries with the heavy atoms fixed at the experimental positions and the H atoms optimised. An electron density file suitable for further analysis was generated from a single-point calculation.

The topology of the electron density was analysed by means of QTAIM (Quantum Theory of Atoms in Molecules),[10] as implemented in the AIMALL package.[11] Inner shell electrons on Rh and P modelled by ECPs were represented by core density functions (extended wavefunction format).

NBO calculations were performed using the NBO 6.0 program,[12] using the same geometries as for the QTAIM calculations above.

NCI calculations were performed using the NCIPLOT program,[13-14] The promolecular electron density was employed.

Orbital plots were created with Chemcraft[15] with an outer contour value of 0.07465.

Conformational searching was carried out using Tinker[16-18] as per the protocol set out in previous work.[19] In all cases the lowest energy conformer corresponded to the crystal structure.

NMR calculations were also performed within the GIAO framework using ADF 2019[20-21] with the B3LYP functional[22] and Slater-type basis sets of double-ζ (DZP) quality.[23] Relativistic effects were treated by the 2-component zeroth-order regular approximation (ZORA).[24]

Electronic Structure Analysis

$[2\text{-Me}]^+$

Table S3. NBO donor acceptor pair 2nd order perturbation theory energies with associated orbital occupations for **[2-Me]**$^+$.

Donating Orbital	Occupation	Accepting Orbital	Occupation	Energy (kcal/mol)
		C1–H1 (Ar)		
σ (C–H)	1.97	σ* (Rh–P)	0.44	0.22
σ (C–H)	1.97	σ* (Rh–P)	0.42	0.47
LP (Rh)	1.97	σ* (C–H)	0.02	2.36
σ (Rh–P)	1.85	σ* (C–H)	0.02	1.29
σ (Rh–P)	1.85	σ* (C–H)	0.02	0.73
		C47–H47a (Me)		
σ (C–H)	1.97	σ* (Rh–P)	0.44	0.31
σ (C–H)	1.97	σ* (Rh–P)	0.22	0.18
LP (Rh)	1.97	σ* (C–H)	0.02	2.53
σ (Rh–P)	1.85	σ* (C–H)	0.02	0.68
σ (Rh–P)	1.85	σ* (C–H)	0.02	1.08

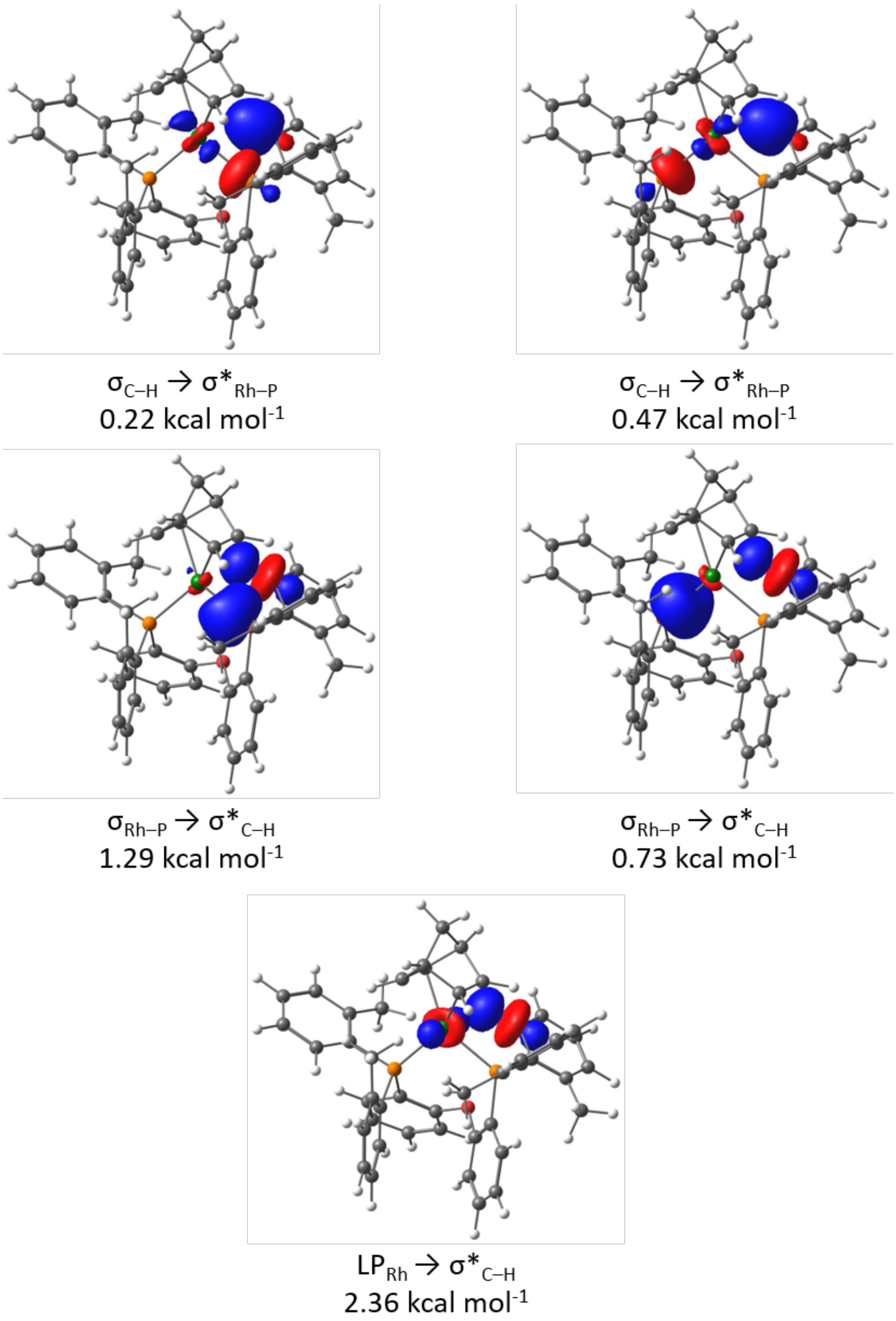

Figure S35. Key NBO donor-acceptor orbital pairs for the C1–H1 bond of **[2-Me]$^+$** with their associated 2nd order perturbation theory energies.

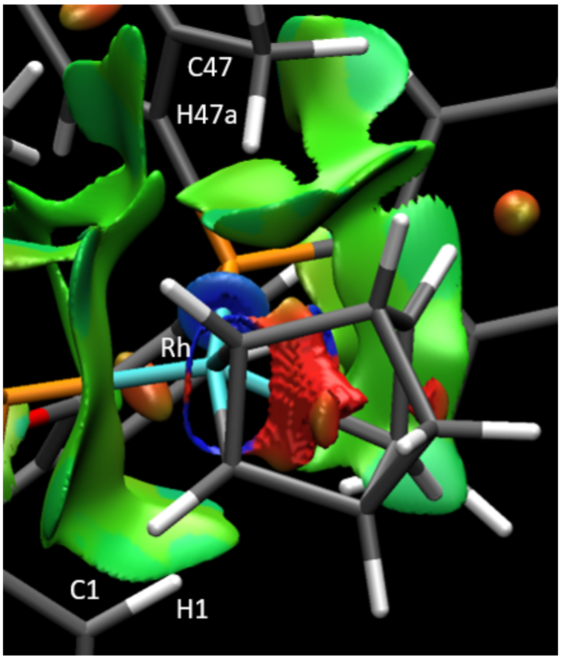

Figure S36. NCI plot of **[2-Me]$^+$** with isosurfaces generated for s = 0.3 au and -0.07 < ρ < 0.07 au. An alternative view highlighting the anagostic motifs with NBD removed can be seen in the main text.

Table S4: Selected QTAIM BCP data and computed interatomic distances for **[2-Me]$^+$**.

**BCP**	**Distance (Å)**	**r(r) (e/bohr^3)**	**∇^2 r(r) (e/bohr^5)**	**H (r)**	**Delocalisation Index**
C1–H1	1.100	0.2738	-0.9426	-0.2763	0.8862
H1–Rh	2.453	0.0219	0.0528	-0.0016	0.0944
C47–H47a	1.108	0.2658	-0.8715	-0.2618	0.8696
H47a–Rh	2.511	0.0203	0.0454	-0.0015	0.0871
Rh–P1	2.351	0.0918	0.1184	-0.0330	0.8156
Rh–P2	2.395	0.0834	0.1300	-0.0267	0.7851

[2-H]$^+$

Table S5. Key NBO donor acceptor pair 2nd order perturbation theory energies with associated orbital occupations for **[2-H]$^+$**.

Donating Orbital	Occupation	Accepting Orbital	Occupation	Energy (kcal/mol)
		C1–H1		
σ (C–H)	1.98	σ* (Rh–P)	0.42	0.28
σ (C–H)	1.98	σ* (Rh–P)	0.43	0.29
LP (Rh)	1.98	σ* (C–H)	0.02	0.65
σ (Rh–P)	1.86	σ* (C–H)	0.02	0.48
σ (Rh–P)	1.86	σ* (C–H)	0.02	0.20
		C36–H36		
σ (C–H)	1.98	σ* (Rh–P)	0.42	0.27
σ (C–H)	1.98	σ* (Rh–P)	0.43	0.25
LP (Rh)	1.98	σ* (C–H)	0.02	0.60
σ (Rh–P)	1.86	σ* (C–H)	0.02	0.16
σ (Rh–P)	1.86	σ* (C–H)	0.02	0.46

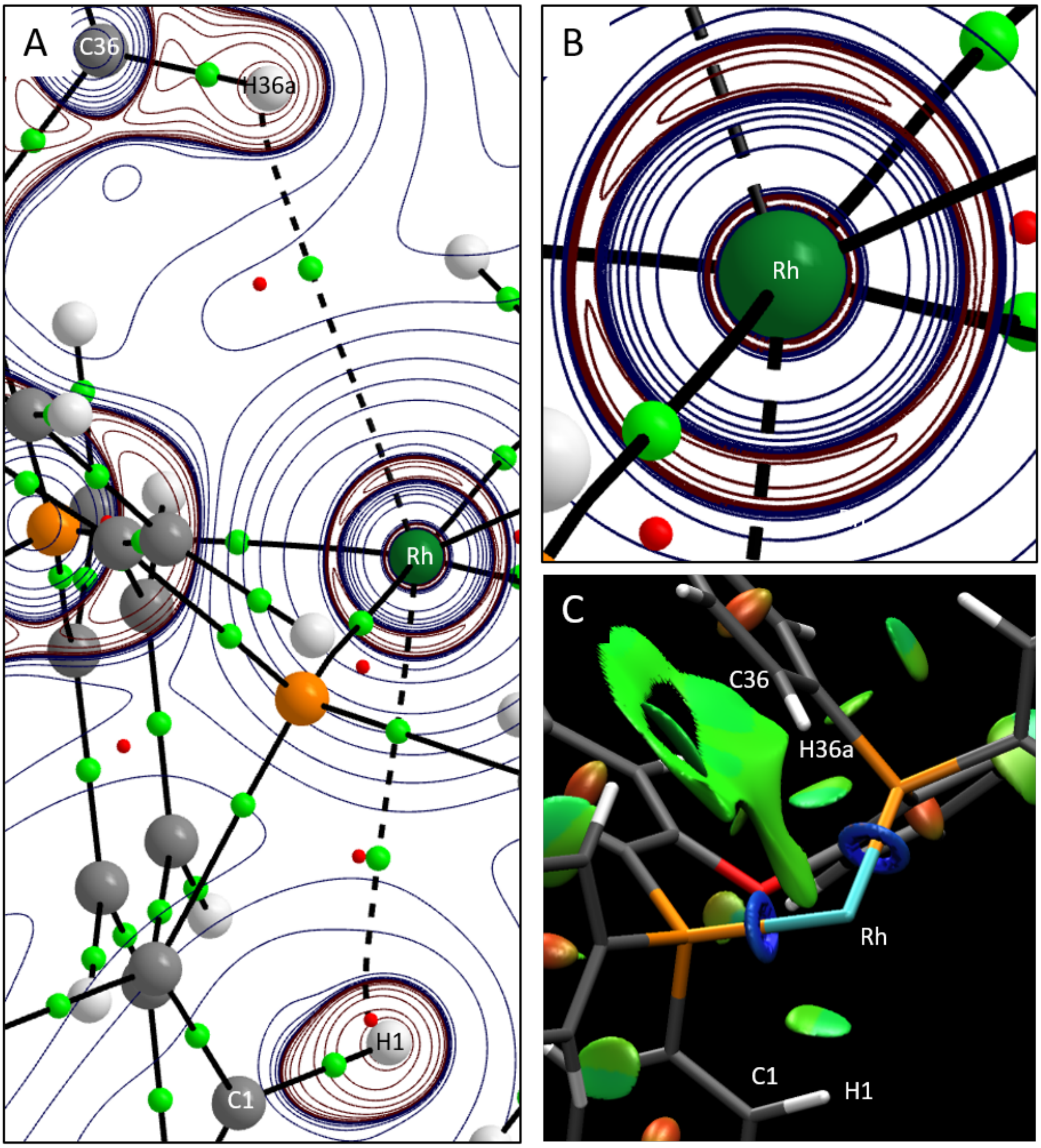

Figure S37. A) QTAIM molecular graph of **[2-H]**$^+$ with a contour plot of the Laplacian in the H-Rh-H plane. B) Close up view of the Laplacian at the Rh centre. C) NCI plot with isosurfaces generated for s = 0.3 au and -0.07 < ρ < 0.07 au of **[2-H]**$^+$ with NBD removed for clarity.

Table S6. Selected QTAIM BCP data and interatomic distances for **[2-H]**$^+$.

BCP	Distance (Å)	ρ(r) (e/bohr^3)	∇^2 ρ(r) (e/bohr^5)	H(r)	Delocalisation Index
C1–H1	1.098	0.2750	-0.9490	-0.2774	0.9139
H1–Rh	2.834	0.0121	0.0357	0.0010	0.0526
C36–H36	1.097	0.2758	-0.9541	-0.2787	0.9150
H36–Rh	2.879	0.0106	0.0305	0.0008	0.0453
Rh–P1	2.344	0.0923	0.1321	-0.0332	0.8403
Rh–P2	2.339	0.0928	0.1214	-0.0337	0.8252

[2-OMe]⁺

Table S7. NBO donor acceptor pair 2nd order perturbation theory energies with associated orbital occupations for **[2-OMe]⁺**.

Donating Orbital	Occupation	Accepting Orbital	Occupation	Energy (kcal/mol)
		C1–H1		
σ (C–H)	1.97	σ* (Rh–P)	0.43	0.19
σ (C–H)	1.97	σ* (Rh–P)	0.43	0.14
LP (Rh)	1.97	σ* (C–H)	0.02	0.92
σ (Rh–P)	1.86	σ* (C–H)	0.02	0.30
σ (Rh–P)	1.86	σ* (C–H)	0.02	0.69

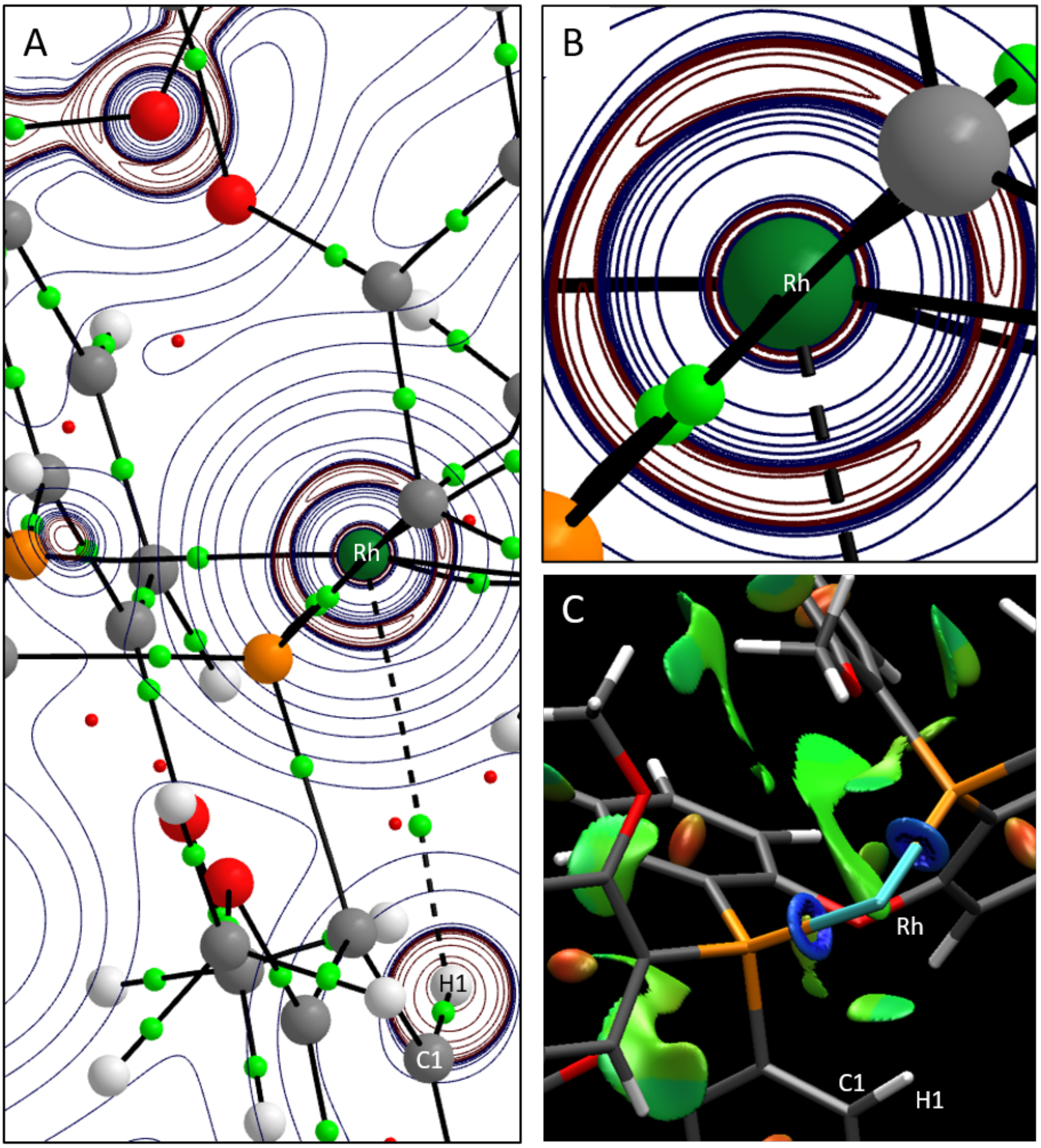

Figure S38: A) QTAIM molecular graph of **[2-OMe]$^+$** with a contour plot of the Laplacian in the H-Rh-H plane. B) Close up view of the Laplacian at the Rh centre. C) NCI plot with isosurfaces generated for s = 0.3 au and -0.07 < ρ < 0.07 au of **[2-OMe]$^+$** with NBD removed for clarity.

Table S8. Selected QTAIM BCP data and interatomic distances for **[2-OMe]$^+$**.

BCP	Distance (Å)	ρ(r) (e/bohr^3)	∇^2 ρ(r) (e/bohr^5)	H (r)	Delocalisation Index
C1–H1	1.096	0.2767	-0.9724	-0.2816	0.9071
H1–Rh	2.787	0.0126	0.0346	0.0006	0.0574
Rh–P1	2.381	0.0853	0.1324	-0.0279	0.7761
Rh–P2	2.337	0.0941	0.1236	-0.0346	0.8327

[2-^{i}Pr]$^{+}$

Table S9. NBO donor acceptor pair 2nd order perturbation theory energies with associated orbital occupations for **[2-^{i}Pr]$^{+}$**.

Donating Orbital	Occupation	Accepting Orbital	Occupation	Energy (kcal/mol)
		C1–H1		
σ (C–H)	1.96	σ* (Rh–P)	0.45	1.29
σ (C–H)	1.96	σ* (Rh–P)	0.45	0.79
LP (Rh)	1.97	σ* (C–H)	0.03	2.83
LP (Rh)	1.67	σ* (C–H)	0.03	0.90
σ (Rh–P)	1.87	σ* (C–H)	0.03	2.31
σ (Rh–P)	1.86	σ* (C–H)	0.03	2.94
		C32–H32		
σ (C–H)	1.97	σ* (Rh–P)	0.45	0.73
σ (C–H)	1.97	σ* (Rh–P)	0.45	0.98
LP (Rh)	1.97	σ* (C–H)	0.03	2.35
LP (Rh)	1.78	σ* (C–H)	0.03	0.12
LP (Rh)	1.67	σ* (C–H)	0.03	0.42
σ (Rh–P)	1.87	σ* (C–H)	0.03	1.96
σ (Rh–P)	1.86	σ* (C–H)	0.03	1.85

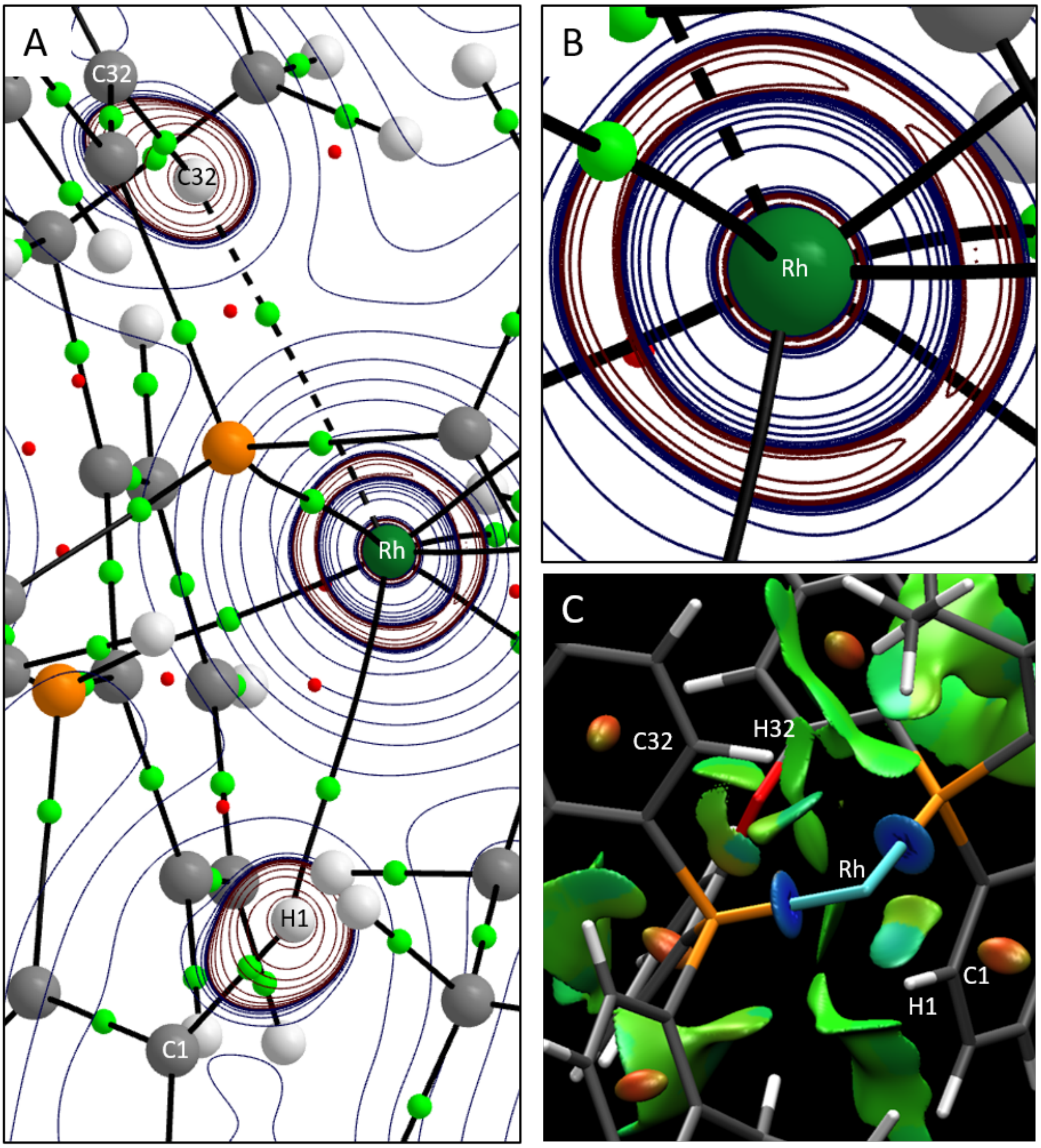

Figure S39. A) QTAIM molecular graph of **[2-^{i}Pr]$^{+}$** with a contour plot of the Laplacian in the H-Rh-H plane. B) Close up view of the Laplacian at the Rh centre. C) NCI plot with isosurfaces generated for s = 0.3 au and -0.07 < ρ < 0.07 au of **[2-^{i}Pr]$^{+}$** with NBD removed for clarity.

Table S10. Selected QTAIM BCP data and interatomic distances for **[2-^{i}Pr]$^{+}$**.

BCP	Distance (Å)	ρ(r) (e/bohr^3)	∇^2 ρ(r) (e/bohr^5)	H (r)	Delocalisation Index
C1–H1	1.105	0.2708	-0.9206	-0.2710	0.8632
H1–Rh	2.334	0.0255	0.0595	-0.0029	0.1156
C32–H32	1.103	0.2722	-0.9319	-0.2733	0.8775
H32–Rh	2.454	0.0208	0.0500	-0.0014	0.0955
Rh–P1	2.448	0.0770	0.1154	-0.0227	0.7218
Rh–P2	2.426	0.0801	0.1188	-0.0247	0.7387

[5-^{i}Pr]$^{+}$

Table S11. NBO donor acceptor pair 2nd order perturbation theory energies with associated orbital occupations for **[5-^{i}Pr]$^{+}$**.

Donating Orbital	Occupation	Accepting Orbital	Occupation	Energy (kcal/mol)
		C38-H38		
σ (C–H)	1.96	σ* (Rh–P)	0.52	0.63
σ (C–H)	1.96	σ* (Rh–O)	0.68	0.51
LP (Rh)	1.96	σ* (C–H)	0.03	1.83
LP (Rh)	1.93	σ* (C–H)	0.03	0.63
LP (P)	1.34	σ* (C–H)	0.03	0.25
σ (Rh–P)	1.86	σ* (C–H)	0.03	0.72
σ (Rh–O)	1.93	σ* (C–H)	0.03	0.16
		C47-H47		
σ (C–H)	1.95	σ* (Rh–P)	0.52	0.46
σ (C–H)	1.95	σ* (Rh–O)	0.68	0.90
LP (Rh)	1.96	σ* (C–H)	0.04	2.76
LP (Rh)	1.93	σ* (C–H)	0.04	0.80
LP (P)	1.34	σ* (C–H)	0.04	0.57
σ (Rh–P)	1.86	σ* (C–H)	0.04	3.74
σ (Rh–O)	1.93	σ* (C–H)	0.04	0.27

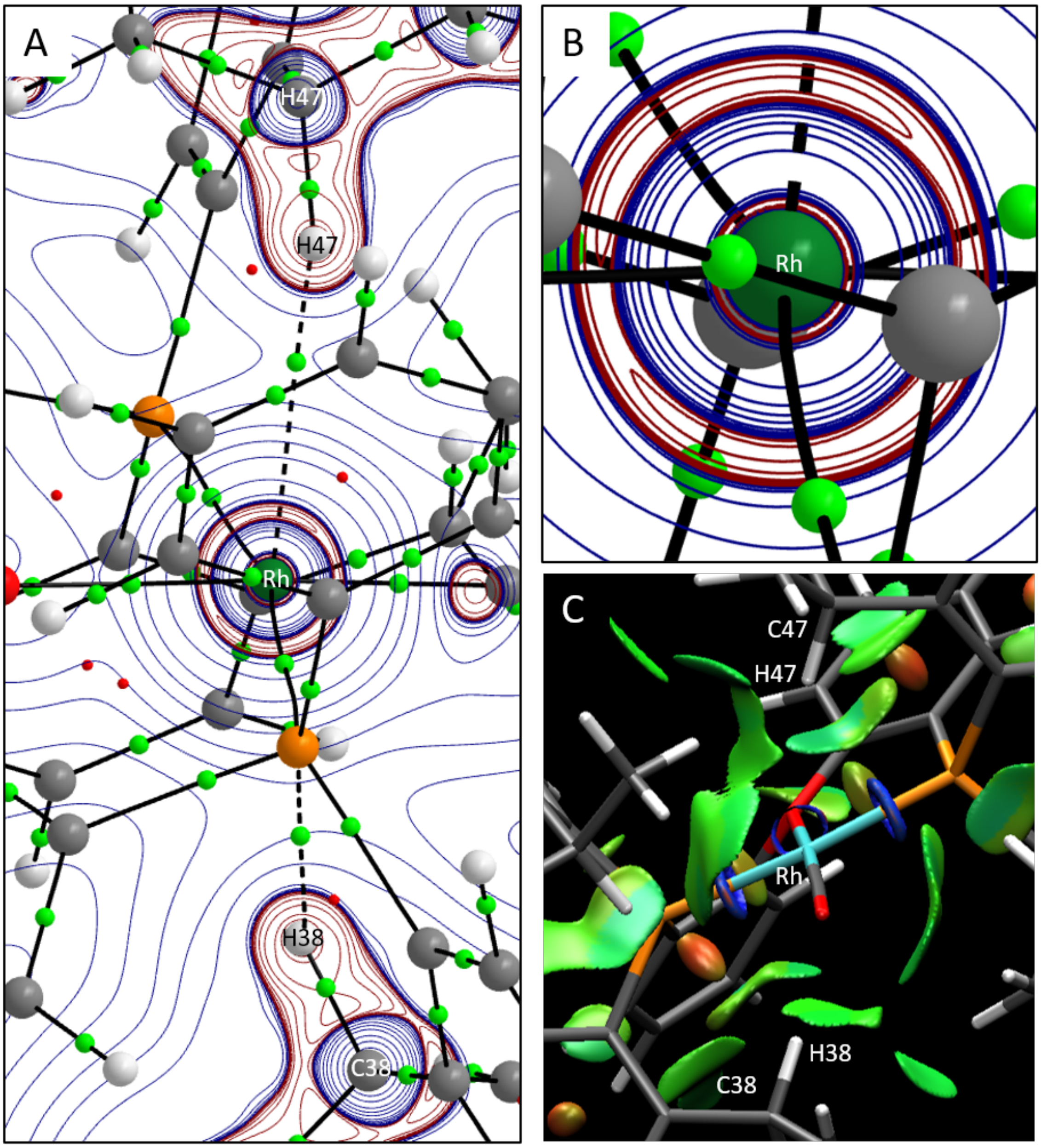

Figure S40. A) QTAIM molecular graph of **[5-^{i}Pr]$^{+}$** with a contour plot of the Laplacian in the H-Rh-H plane. B) Close up view of the Laplacian at the Rh centre. C) NCI plot with isosurfaces generated for s = 0.3 au and -0.07 < ρ < 0.07 au of **[5-^{i}Pr]$^{+}$**.

Table S12: Selected QTAIM BCP data and interatomic distances for **[5-^{i}Pr]$^{+}$**. *No bond path or BCP was found between H38 and Rh. Instead a H38-P3 bond path was identified.

BCP	Distance (Å)	ρ(r) (e/bohr^3)	∇^2 ρ(r) (e/bohr^5)	H (r)	Delocalisation Index
C38–H38	1.111	0.2688	-0.8768	-0.2636	0.8628
H38–Rh	2.703		No BCP*		
C47–H47	1.115	0.2659	-0.8599	-0.2587	0.8511
H47–Rh	2.544	0.0191	0.0422	-0.0010	0.1010
Rh–P1	2.314	0.0979	0.1167	-0.0380	0.8392
Rh–P2	2.303	0.1002	0.1107	-0.0402	0.8356

[6-^{i}Pr]$^{+}$

Table S13. NBO donor acceptor pair 2nd order perturbation theory energies with associated orbital occupations for **[6-^{i}Pr]$^{+}$**.

Donating Orbital	Occupation	Accepting Orbital	Occupation	Energy (kcal/mol)
		C46–H46		
σ (C–H)	1.95	σ* (Rh–P)	0.39	0.44
σ (C–H)	1.95	σ* (Rh–P)	0.42	0.95
LP (Rh)	1.96	σ* (C–H)	0.04	3.26
LP (Rh)	1.93	σ* (C–H)	0.04	1.19
LP (Rh)	1.91	σ* (C–H)	0.04	0.53
LP (Rh)	1.73	σ* (C–H)	0.04	0.16
σ (Rh–P)	1.85	σ* (C–H)	0.04	4.31
σ (Rh–P)	1.85	σ* (C–H)	0.04	1.66
		C49–H49		
σ (C–H)	1.96	σ* (Rh–P)	0.39	0.73
σ (C–H)	1.96	σ* (Rh–P)	0.42	0.39
LP (Rh)	1.96	σ* (C–H)	0.04	2.50
LP (Rh)	1.93	σ* (C–H)	0.04	1.09
σ (Rh–P)	1.85	σ* (C–H)	0.04	1.94
σ (Rh–P)	1.85	σ* (C–H)	0.04	4.06
		B1-H1B (Agostic)		
σ (B–H)	1.69	σ* (Rh–P)	0.39	4.29
σ (B–H)	1.69	σ* (Rh–P)	0.42	48.09
LP (Rh)	1.96	σ* (B–H)	0.05	0.18
LP (Rh)	1.91	σ* (B–H)	0.05	2.23
LP (Rh)	1.73	σ* (B–H)	0.05	1.73
σ (Rh–P)	1.85	σ* (B–H)	0.05	2.75
σ (Rh–P)	1.85	σ* (B–H)	0.05	0.44

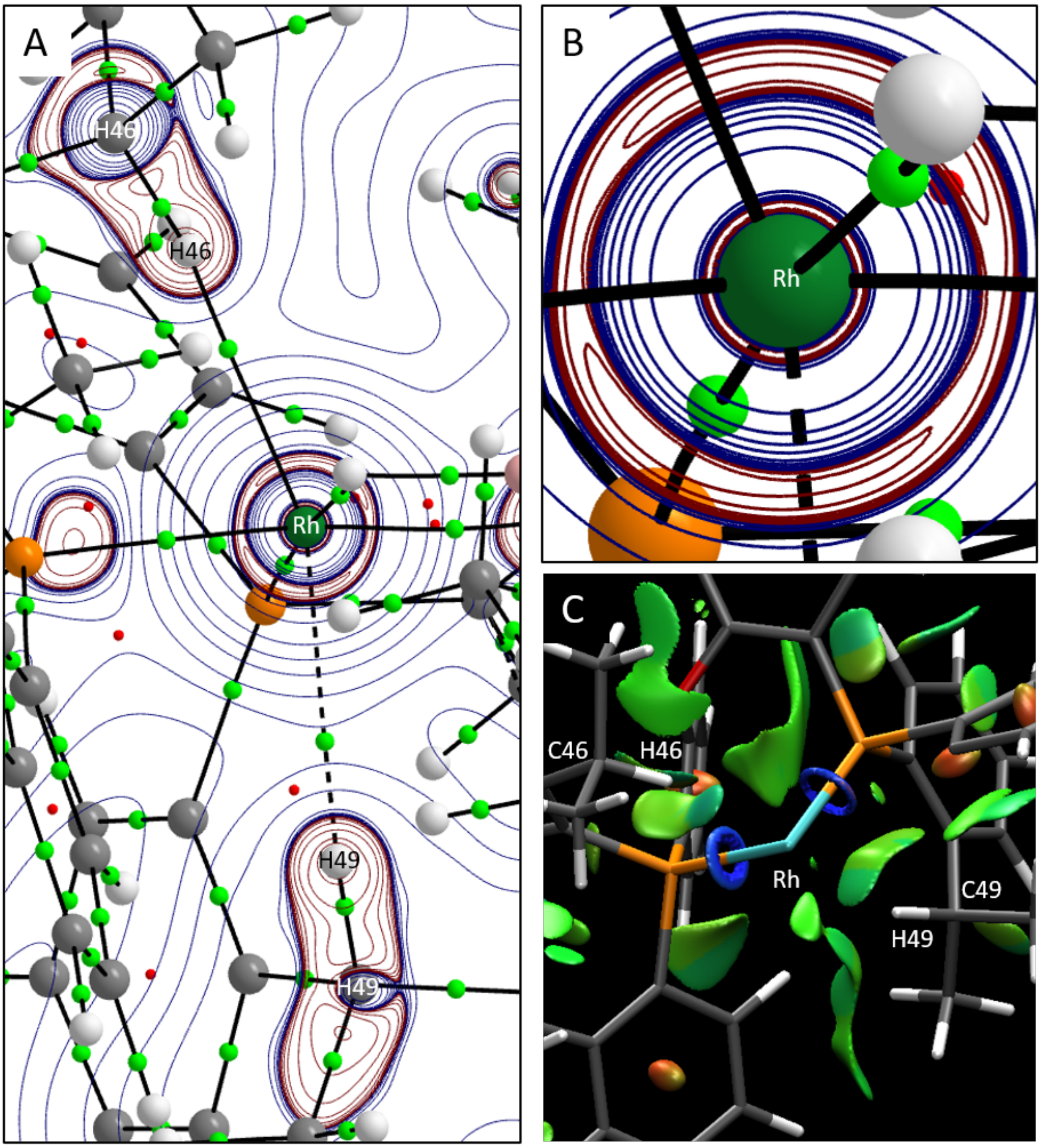

Figure S41. A) QTAIM molecular graph of **[6-^{i}Pr]$^{+}$** with a contour plot of the Laplacian in the H-Rh-H plane. B) Close up view of the Laplacian at the Rh centre. C) NCI plot with isosurfaces generated for s = 0.3 au and -0.07 < ρ < 0.07 au of **[6-^{i}Pr]$^{+}$**. The $C_6H_{15}NB$ fragment has been removed for clarity.

Table S14. Selected QTAIM BCP data and interatomic distances for **[6-^{i}Pr]$^{+}$**.

BCP	Distance (Å)	ρ(r) (e/bohr^3)	∇^2 ρ(r) (e/bohr^5)	H (r)	Delocalisation Index
C46–H46	1.118	0.2644	-0.8518	-0.2563	0.8307
H46–Rh	2.334	0.0266	0.0540	-0.0036	0.1338
C49–H49	1.118	0.2671	-0.8714	-0.2610	0.8506
H49–Rh	2.617	0.0177	0.0383	-0.0007	0.0925
Rh–P1	2.336	0.0932	0.1301	-0.0338	0.8585
Rh–P2	2.270	0.1035	0.1346	-0.0427	0.9694
B1–H	1.350	0.1188	-0.1238	-0.1102	0.3391
H–Rh	1.769	0.0843	0.2288	-0.0239	0.5204

Computed Atomic Charges

Table S15. Selected QTAIM and NBO charges for all species.

Anagostic / Spectator	Atom Number	QTAIM Charge	NBO Charge
	**$[2\text{-}Me]^+$**		
Anagostic	H1	0.0261	0.24058
Anagostic	C1	-0.0248	-0.20345
Spectator	H8	0.0365	0.26367
Spectator	C8	-0.0233	-0.23751
Anagostic	H47A	0.0273	0.24679
Anagostic	C47	-0.0070	-0.71613
Spectator	H47B	0.0091	0.26161
	H47C	0.0165	0.26243
Spectator	H46A	0.0079	0.25488
	H46B	0.0115	0.26501
	H46C	0.0340	0.28031
Spectator	C46	0.0025	-0.72845
Rhodium	Rh1	0.2384	-0.05316
	**$[2\text{-}H]^+$**		
Anagostic	H1	0.0309	0.26159
Anagostic	C1	-0.0245	-0.24391
Anagostic	H36	0.0281	0.25382
Anagostic	C36	-0.0083	-0.22490
Spectator	H26	0.0293	0.26101
Spectator	C26	-0.0271	-0.25087
Rhodium	Rh1	0.2239	-0.08747
	**$[2\text{-}OMe]^+$**		
Anagostic	H1	0.0473	0.26407
Anagostic	C1	0.0120	-0.20689
Spectator	H8	0.0322	0.25834
Spectator	C8	-0.0069	-0.21414
Rhodium	Rh1	0.2886	-0.03110

Table S15 (cont.).

	**[2-^{i}Pr]$^{+}$**		
Anagostic	H32	0.0273	0.24192
Anagostic	C32	-0.0179	-0.20912
Anagostic	H1	0.0244	0.23647
Anagostic	C1	-0.0288	-0.21186
Spectator	H26	0.0344	0.26217
Spectator	C26	-0.0675	-0.23602
Rhodium	Rh1	0.2661	-0.01343
	**[5-^{i}Pr]$^{+}$**		
Anagostic	H38	-0.0066	0.25712
Anagostic	C38	0.0698	-0.28352
Anagostic	H47	-0.0010	0.25443
Anagostic	C47	0.0615	-0.28187
Spectator	H44	-0.0221	0.25311
Spectator	C44	0.0776	-0.28107
Rhodium	Rh1	0.2621	-0.23191
	**[6-^{i}Pr]$^{+}$**		
Anagostic	H46	0.0058	0.24762
Anagostic	C46	0.0638	-0.26597
Anagostic	H49	0.0057	0.25897
Anagostic	C49	0.0543	-0.28438
Spectator	H43	0.0147	0.27558
Spectator	C43	0.0782	-0.29004
Agostic	H1B	-0.4183	0.03366
Agostic	B1	1.8492	0.11418
Agostic	H1A		
Spec.		-0.5865	0.02784
Rhodium	Rh1	0.1106	-0.24944

Comparison of BCP Metrics with Rh···H distances

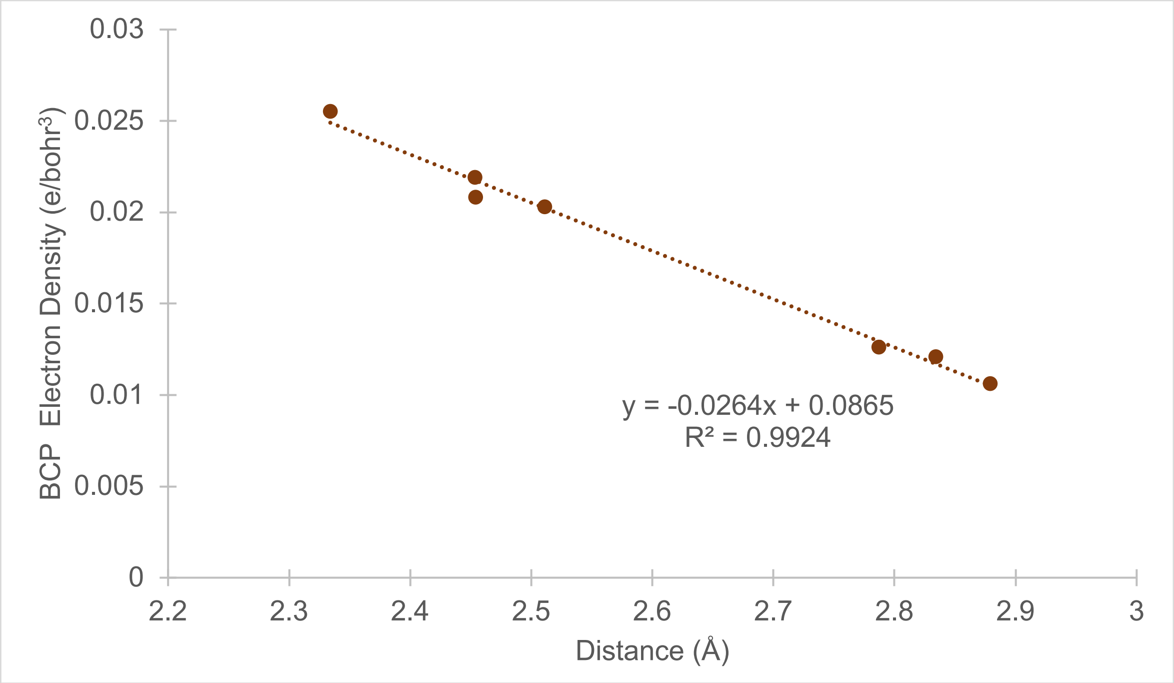

Figure S42. Plot of Rh···H BCP electron densities against Rh···H distance.

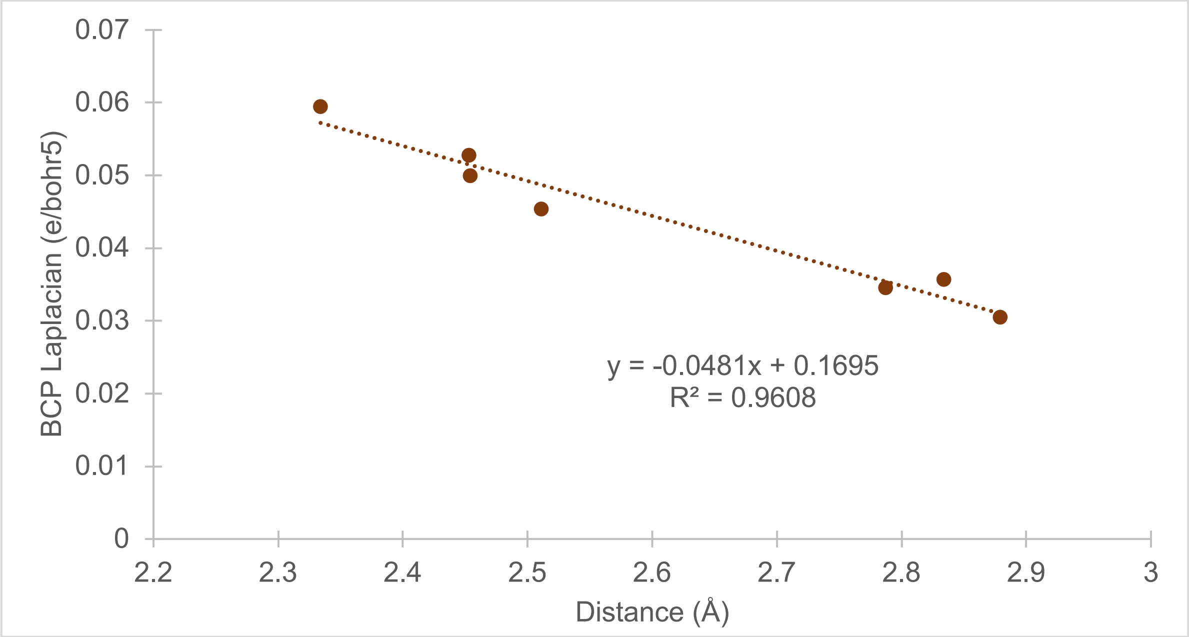

Figure S43. Plot of Rh···H BCP Laplacian against Rh···H distance.

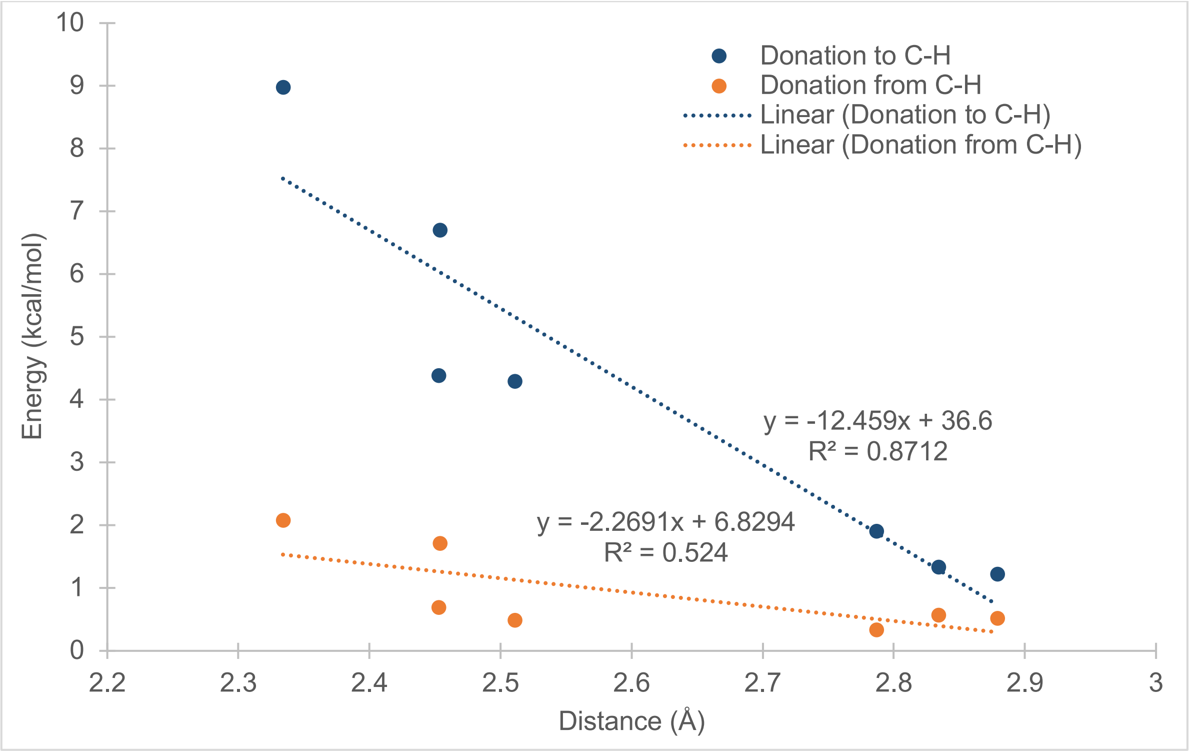

Figure S44. Plot of NBO $\sigma_{C\text{-}H}\rightarrow$Rh donation (orange) and Rh$\rightarrow\sigma^*_{C\text{-}H}$ donation (blue) against Rh···H distance.

Computed NMR Data

Data were computed for **[2-R]⁺** (R = Me, H. OMe, ^{i}Pr), **[5-^{i}Pr]⁺** and **[6-^{i}Pr]⁺** (see Table S16) and are quoted relative to TMS set to 0 ppm. Following a conformational search and computation of the corrected free energies, ^{1}H chemical shifts were computed for the lowest energy conformer and all conformers within 5.0 kcal mol^{-1} of that species. Boltzmann-averaged chemical shifts were then taken, and these are reported in the main paper. The heavy-atom fixed geometries derived from the crystal structures were also analysed and showed only minor differences to the Boltzmann-averaged values in solution.

Table S16. Selected computed NMR shifts (ppm) for anagostic hydrogens in **[2-R]$^{+}$** (R = Me, H. OMe, ^{i}Pr), **[5-^{i}Pr]$^{+}$** and the B–H hydrogens in **[6-^{i}Pr]$^{+}$**.

Conformation/ Optimisation Protocol	Anagostic H 1 Type	NMR Shift 1	Anagostic H 1 Type	NMR Shift 2
		**[2-Me]$^{+}$**		
H. Atom Fixed	Ar	10.466	Me	5.283 (3.643)[a]
Conf 1 (+0.00)	Ar	10.567	Me	5.980 (3.934)[a]
Conf 2 (+2.19)	Ar	10.118	Ar	10.357[b]
Conf 3 (+3.29)	Ar	10.613	Me	5.963 (4.121)[a]
**Boltzmann Avg.**	**Ar**	**10.556**	**Me**	**3.935**
[a]Data in parentheses are the average of the 3 methyl protons and these are used to calculate the Boltzmann average where appropriate; [b]not included in the Boltzmann averaging				
		**[2-H]$^{+}$**		
H. Atom Fixed	Ar	8.762	Ar	9.027
Conf 1 (+0.00)	Ar	8.993	Ar	9.483
Conf 2 (+0.62)	Ar	9.379	-	-
**Boltzmann Avg.**	**Ar**	**9.093**	**-**	**-**
		**[2-OMe]$^{+b}$**		
H. Atom Fixed	Ar	9.677	-	-
Conf 1 (+0.00)	Ar	10.060	-	-
Conf 2 (+0.13)	Ar	9.017	-	-
Conf 3 (+0.49)	-	-	-	-
Conf 4 (+0.69)	Ar	9.820	Ar	8.539
Conf 5 (+0.78)	-	-	Ar	9.542
Conf 6 (+1.68)	-	-	-	-
Conf 7 (+2.07)	Ar	9.910	Ar	9.698
Conf 8 (+2.74)	Ar	9.664	-	-
Conf 9 (+3.64)	Ar	9.450	Ar	9.554
Conf 10 (+3.76)	-	-	Ar	8.640
Conf 11 (+4.39)	Ar	9.698	Ar	8.120
**Boltzmann Avg.**		**9.633**		**9.037**
[b] In **[2-OMe]$^{+}$** a Rh···O contact is seen resulting in only one anagostic Rh···H–C motif. Conformers with this single anagostic Rh···H–C motif are shown in the left-hand column and the Boltzmann average is based on these data. Additional conformers featuring either two anagostic Rh···H–C motif or a Rh···H–C motif in the place of the Rh···O contact were also located and data are shown in the right-hand column. The two arrangements had relative populations of 3.509: 1				

Table S16 (cont.).

		**$[2\text{-}^{i}Pr]^{+}$**		
H. Atom Fixed	Ar	9.553	Ar	9.600
Conf 1 (+0.00)	Ar	9.829	Ar	9.932
		**$[5\text{-}^{i}Pr]^{+}$**		
H. Atom Fixed	^{i}Pr	3.979	^{i}Pr	5.020
Conf 1 (+0.00)	^{i}Pr	4.597	^{i}Pr	4.597
Conf 2 (+2.99)	-	-	^{i}Pr	4.328/5.555
**Boltzmann Avg.**	**-**	**-**	**^{i}Pr**	**4.601**
		**$[6\text{-}^{i}Pr]^{+}$ (Anagostic Hydrogens)**		
H. Atom Fixed	^{i}Pr	5.333	^{i}Pr	4.697
Conf 1 (+0.00)	^{i}Pr	5.154	^{i}Pr	4.354
Conf 2 (+1.81)	Ar	9.164	^{i}Pr	4.621
Conf 3 (+1.86)	-	-	^{i}Pr	5.113
**Boltzmann Avg.**	**-**	**-**	**^{i}Pr**	**4.396**
		**$[6\text{-}^{i}Pr]^{+}$ (B-H Hydrogens)**		
H. Atom Fixed	Agostic H	-5.594	Terminal B-H	2.309
Conf 1 (+0.00)	Agostic H	-6.423	Terminal B-H	2.223
Conf 2 (+1.81)	Agostic H	-6.568	Terminal B-H	2.221
Conf 3 (+1.86)	Agostic H	-6.554	Terminal B-H	2.344
**Boltzmann Avg.**	**Agostic H**	**-6.434**	**Terminal B-H**	**2.228**

Hydrogenation of 2-Me

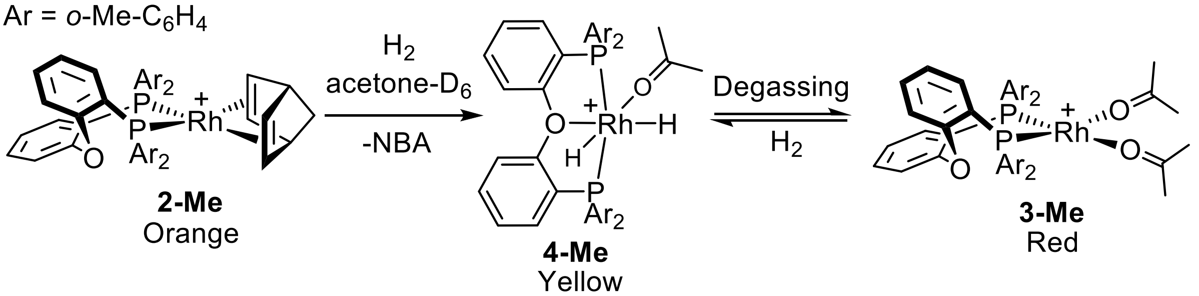

Scheme S3. Formation of **3-Me** and **4-Me** from hydrogenation of **2-Me**. $[BAr^F_4]^-$ anions omitted for clarity.

A sample of **2-Me** (~20 mg) was dissolved in 0.5 ml acetone-D_6 in a high-pressure J. Youngs NMR tube and degassed by three successive freeze-pump thaws. The NMR tube was then put under an atmosphere of 2 bar H_2, shaken, then left for 30 minutes. The solution turned from orange to yellow and the structure of the new complex formed was proposed to be a dihydride specie, **4-Me**, through in-situ NMR studies. The sample was then degassed again by five successive freeze-pump-thaws and put under an atmosphere of argon, resulting in a colour change to red, typical of a solvated complex. In-situ NMR experiments suggested conversion of the dihydride complex to an acetone bound specie, **3-Me**. Persistent vacuum on removal of solvent resulted in decomposition of **3-Me**.

3-Me

3-Me has been tentatively assigned as a bis-acetone complex similar to those previously reported with DPEphos-H, $[Rh(DPEphos\text{-}Me)(acetone)_2][BAr^F_4]$.[7] Observation of large $J_{Rh(I)P}$ (194 and 199 Hz) in the room temperature $^{31}P\{^1H\}$ NMR spectrum are indicative of weakly coordinating solvent ligands in a $[Rh(I)(diphosphine)(acetone)_2][BAr^F_4]$ complex.[7] In the ^1H NMR spectrum, free norbornane (NBA), broad signals in the CH_3 and aromatic regions and no hydride signals are observed. A broad signal at 10.73 ppm in the ^1H NMR suggests an anagostic motif could be present in the Rh(I) complex as previously described in **2-R** (*vide infra*).

$^{31}P\{^1H\}$ NMR (202 MHz, acetone-D_6, 298 K): δ 39.6 (br d, J_{RhP} = 194 Hz) and 29.0 (br d, J_{RhP} = 199 Hz).

^1H NMR (500 MHz, acetone-D_6, 298 K): δ 10.73 (br s, 1H, Ar), 7.96 (m, 1H, Ar), 7.79 (8H, *o*-C*H* BAr^F_4), 7.67 (s, 4H, *p*-C*H* BAr^F_4), 7.63-5.75 (complex m, 22H, Ar), 3.53 (br s, 3H, Ar-CH_3), 2.67 (br multiplet, 6H, Ar-CH_3), 2.35 (br s, 3H, Ar-CH_3).

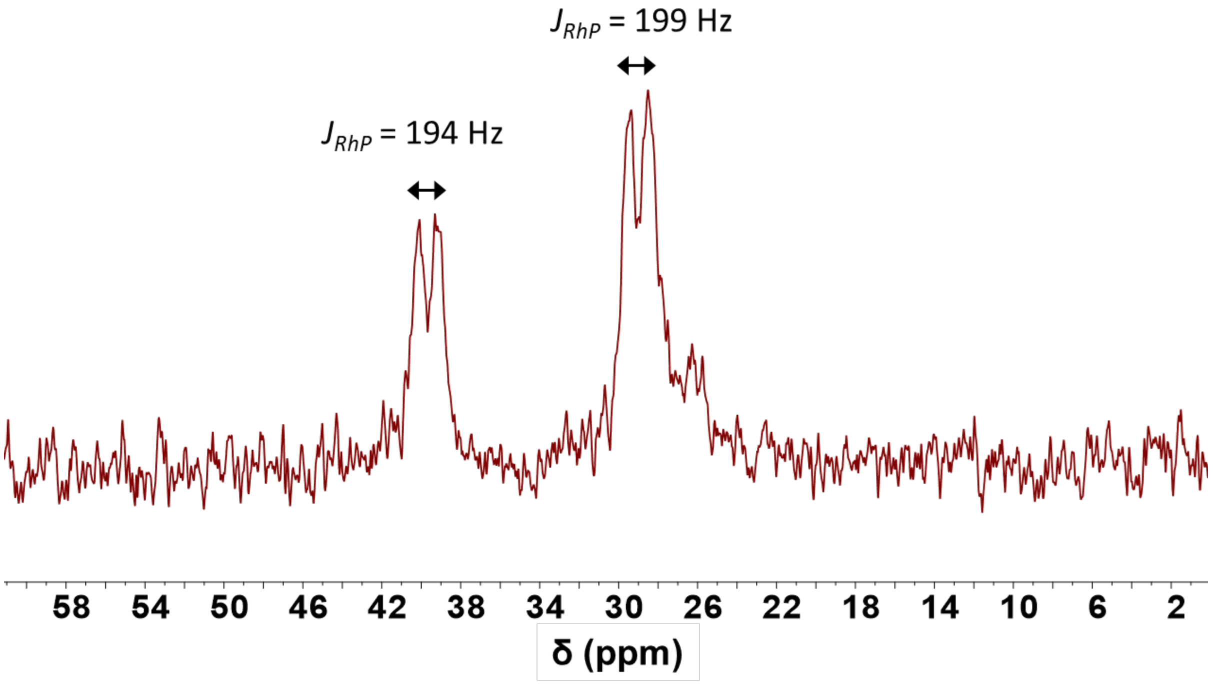

Figure S45. $^{31}P\{^{1}H\}$ NMR spectrum of acetone bound species **3-Me** (acetone-D$_6$, 202 MHz, 298 K).

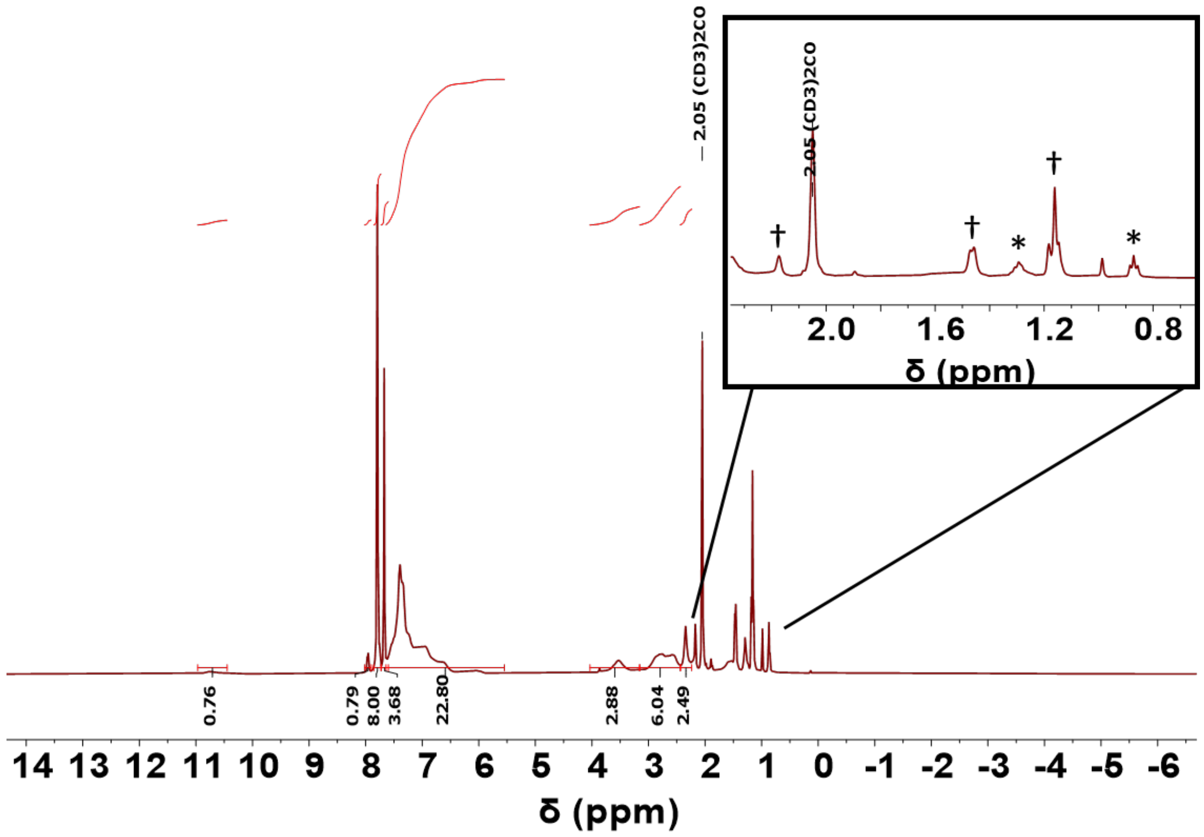

Figure S46. ^{1}H NMR spectrum of **3-Me** (acetone-D$_6$, 500 MHz, 298 K). * Denotes pentane impurity and † denotes free NBA.

4-Me

Although, the room temperature ^{31}P signal in the $^{31}P\{^1H\}$ NMR spectrum of **4-Me** is broad (26 ppm), once the sample was cooled to 183 K, two distinct ^{31}P signals were observed as doublet of doublets, containing large *trans* J_{PP} (343 Hz) and smaller J_{RhP} (121 and 114 Hz). These suggest a trans arrangement of the ^{31}P nuclei with a fluxional process occurring on the NMR timescale and the low temperature regime exists in an asymmetric arrangement. In the ^1H NMR spectrum at 298 K a broad hydride signal was observed at δ -19.51 with integrals 2H relative to the total of the aromatic signals. The methyl signals at this temperature are also broad (2.1-2.5 ppm). Upon cooling, this hydride signal splits into two major signals and two minor signals in a 5:1 ratio that integrate in total to 2H, suggestive of two isomers with very similar structures. The multiplicity of the hydride signal in the major component could be deciphered as a dddd, comprising of J_{RhH}, 2 x $J_{P(\mathrm{cis})H}$, and $J_{H(cis)H}$. These could deduced as ca. 30, 12 [both $J_{P(\mathrm{cis})H}$], and 8 Hz respectively through selective decoupling experiments. The methyl signals also separate, with eight observable signals (two asymmetric isomers). This data suggests the formation of two isomers of a dihydride complex, possibly caused by restricted rotation of the substituted aryl groups. Assuming a similar arrangement to the Xantphos equivalent, $[Rh(H)_2(Xantphos)(acetone)][BAr^F{}_4]$,[8] there is also likely to be an acetone molecule bound, which rapid coordination/de-coordination could be the cause of the fluxionality observed.

$^{31}P\{^1H\}$ NMR (202 MHz, acetone-D$_6$, 298 K): δ 26.0 (br s).

$^{31}P\{^1H\}$ NMR (202 MHz, acetone-D$_6$, 183 K): δ 43.1 (dd, J_{RhP} = 121 Hz, J_{PP} = 343 Hz) and 23.3 (dd, J_{RhP} = 114 Hz, J_{PP} = 343 Hz).

^1H NMR (500 MHz, acetone-D$_6$, 298 K): δ 7.97 (br d, 2H, *J* = H, Ar), 7.85-7.72 (overlapping signals, 10 H, *o*-C*H* BAr$^F{}_4$ and Ar), 7.67 (s, 4H, *p*-C*H* BAr$^F{}_4$), 7.62-7.18 (br multiplet, 20 H, Ar), 2.50-2.10 (br multiplet, 12H, CH_3), -19.51 (br s, 2H, Rh-*H*).

^1H NMR (500 MHz, acetone-D$_6$, 183 K) major isomer: δ 8.28 (multiplet, 2H, Ar), 7.98-7.81 (overlapping signals, 10 H, *o*-C*H* BAr$^F{}_4$ and Ar), 7.77 (s, 4H, *p*-C*H* BAr$^F{}_4$), 7.69 (multiplet, 2H, Ar), 7.62-7.18 (br multiplet, 16 H, Ar), 2.55 (s, 3H, CH_3), 2.36 (s, 3H, CH_3), 2.08 (s, 3H, CH_3), 1.87 (s, 3H, CH_3), -18.36 (dddd, 1H, J_{RhH} = 24 Hz, $J_{P(cis)H}$ = 12 Hz, $J_{P(cis)H}$ = 12 Hz, $J_{H(cis)H}$ = 8 Hz, Rh-*H*) and -20.32 (dddd, 1H, J_{RhH} = 30 Hz, $J_{P(cis)H}$ = 12 Hz, $J_{P(cis)H}$ = 12 Hz, $J_{H(cis)H}$ = 8 Hz, Rh-*H*)).

$^1H\{^{31}P\}$ NMR (500 MHz, acetone-D$_6$, 183 K) major isomer, selected data: δ -18.34 (dd, 1H, J_{RhH} = 24 Hz and $J_{H(cis)H}$ = 8 Hz, Rh-*H*) and -20.31 (dd, 1H, J_{RhH} = 30 Hz and $J_{H(cis)H}$ = 8 Hz, Rh-*H*).

^1H NMR (500 MHz, acetone-D$_6$, 183 K) minor isomer, selected data: δ 4.18 (s, 3H, CH_3), 3.81 (s, 3H, CH_3), 3.68 (s, 3H, CH_3), 2.47 (s, 3H, CH_{3f}), -18.02 (br s, 1H, J_{RhH} = 24 Hz, $J_{P(cis)H}$ = 12 Hz, $J_{P(cis)H}$ = 12 Hz, $J_{H(cis)H}$ = 8 Hz, Rh-*H*) and -19.97 (dddd, 1H, J_{RhH} = 30 Hz, $J_{P(cis)H}$ = 12 Hz, $J_{P(cis)H}$ = 12 Hz, $J_{H(cis)H}$ = 8 Hz, Rh-*H*)).

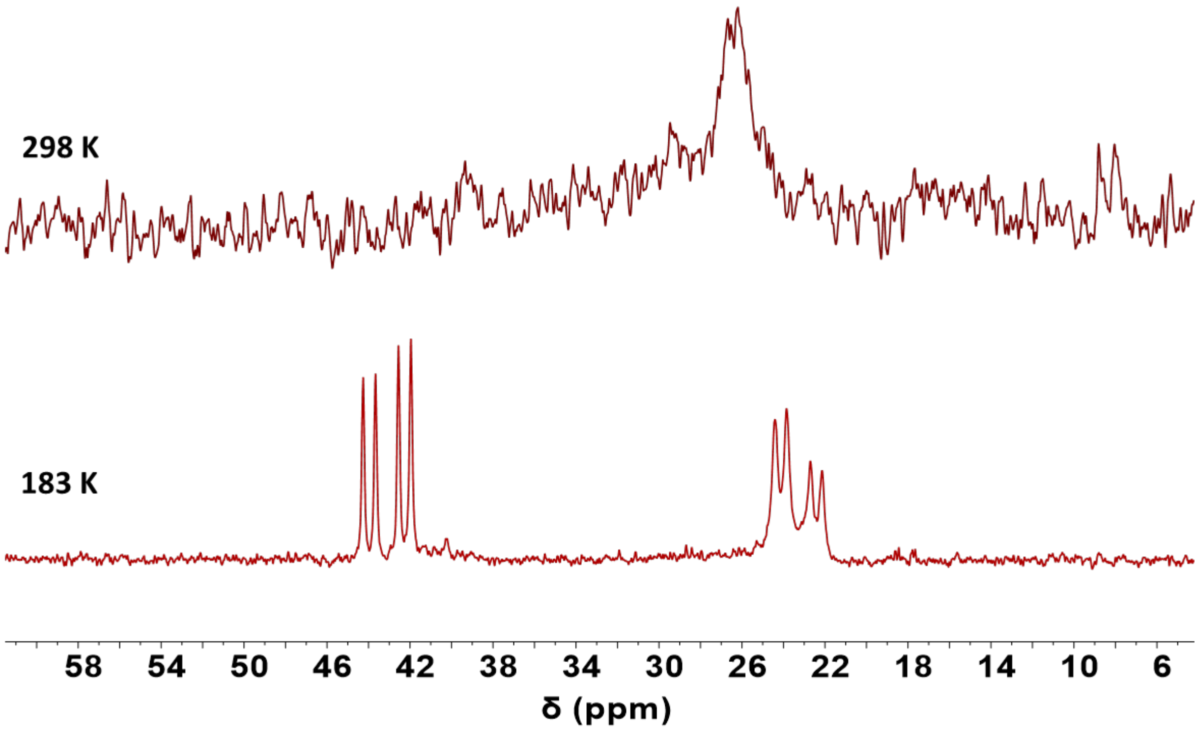

Figure S47. $^{31}P\{^{1}H\}$ NMR spectra of **4-Me** at 298 K and 183 K (202 MHz, acetone-D$_6$).

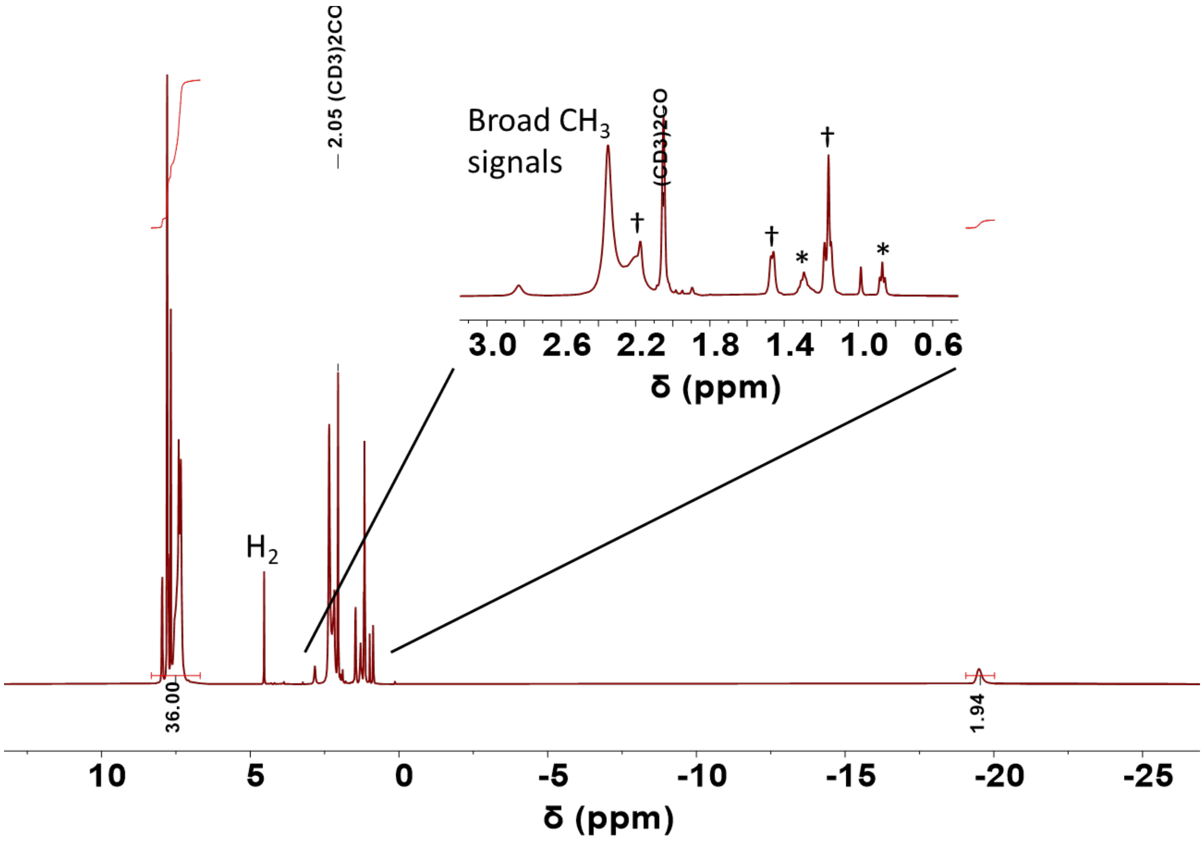

Figure S48. ^{1}H NMR spectrum of **4-Me** (500 MHz, acetone-D$_6$, 298 K). † Denotes free NBA and * shows pentane impurity.

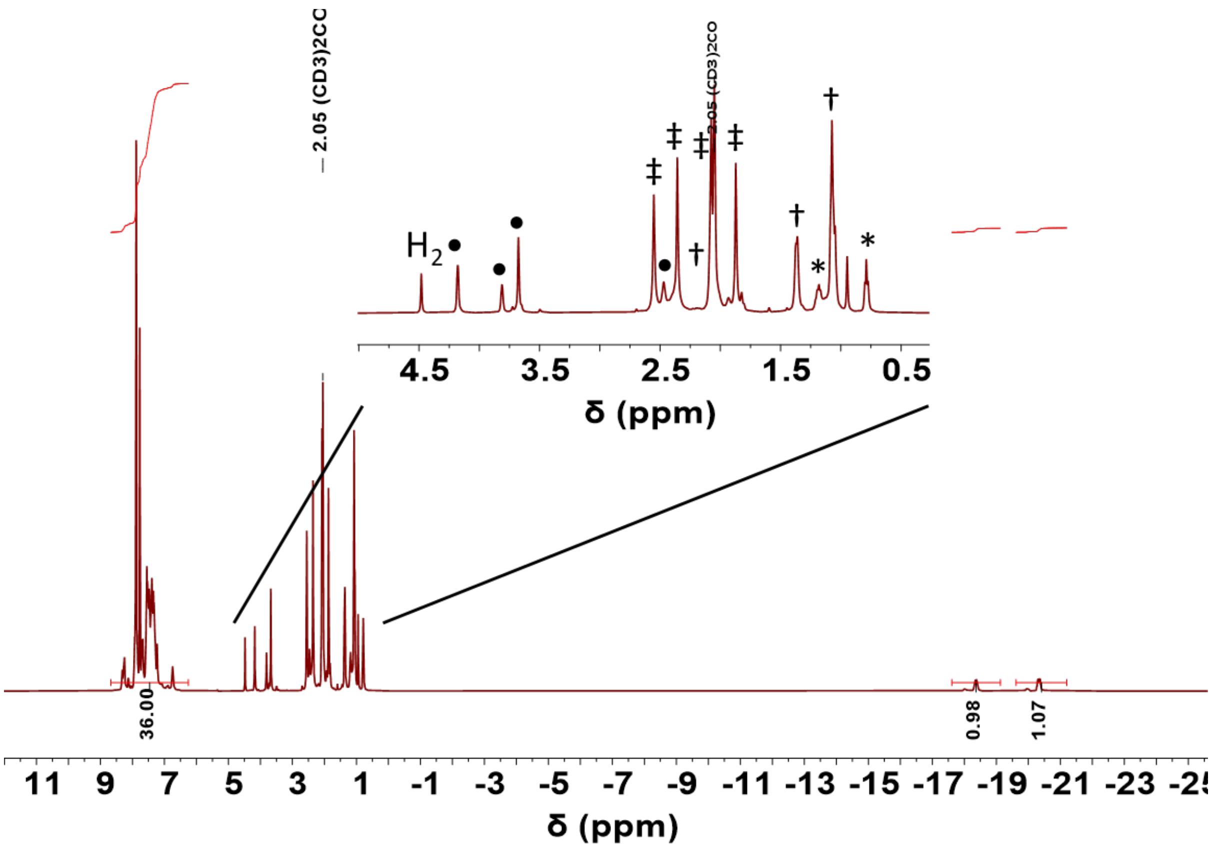

Figure S49. ^{1}H NMR spectrum of **4-Me** (500 MHz, acetone-D$_6$, 183 K). † Denotes free NBA and * shows pentane impurity and • and ‡ are the four different CH_3 environments of the minor and major isomers respectively.

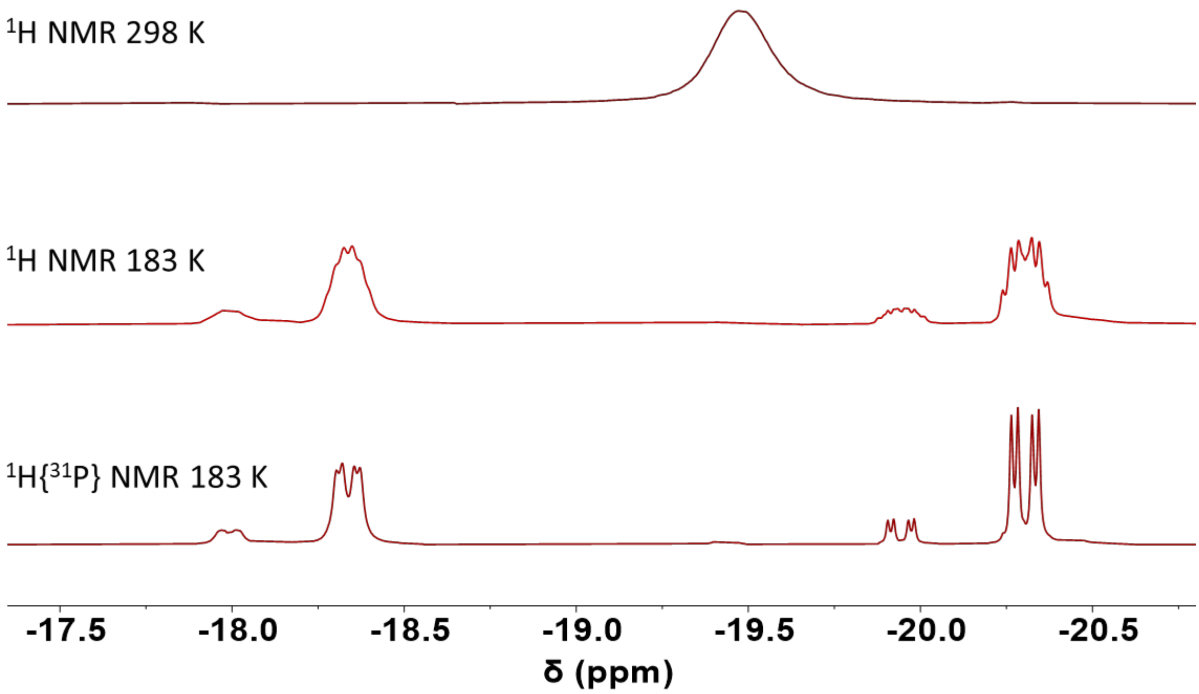

Figure S50. The hydride region of the ^{1}H NMR spectra of **4-Me** at 298 and 183 K (500 MHz, acetone-D$_6$).

Hydrogenation of 2-OMe

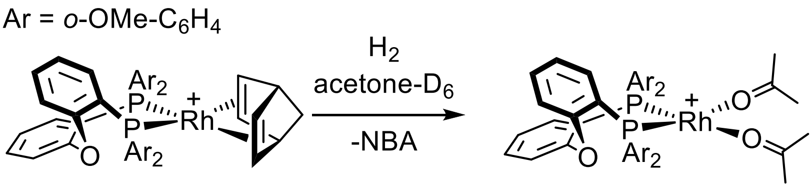

Scheme S4. Formation of **3-OMe** from the hydrogenation of **2-OMe**. $[BAr^F{}_4]^-$ anions omitted for clarity.

A sample of **2-OMe** (~20 mg) was dissolved in 0.5 ml acetone-D_6 in a high-pressure J. Youngs NMR tube and degassed by three successive freeze-pump thaws. The NMR tube was then put under an atmosphere of 2 bar H_2 then shaken and left for 30 minutes, resulting in a colour change from orange to red. The solution was then degassed again by five successive freeze-pump-thaws and put under an atmosphere of argon. The spectroscopic data did not change upon H_2 removal, which suggests no hydride formation. Persistent vacuum on removal of solvent resulted in decomposition of the resultant species.

3-OMe is tentatively characterised as a bis-acetone complex, [Rh(DPEphos-OMe)(acetone)$_2$][BAr$^F{}_4$]. Although mostly broad signals are observed in the room temperature ^{31}P and ^{1}H NMR spectra, free NBA and absence of hydride signals supports formation of [Rh(DPEphos-OMe)(acetone)$_2$][BAr$^F{}_4$], similar to the parent DPEphos-H equivalent.[7] At 193 K, multiple different isomers were observed in the ^{31}P{^{1}H} NMR spectrum with large J_{RhP} (205-223 Hz) and no observable *trans*-J_{PP}. There appears to be at least two asymmetric isomers with mutually coupled ^{31}P signals ($J_{PP(cis)}$ = 58 Hz) and one isomer of a symmetric orientation with no observable J_{PP}. These relatively large $J_{PP(cis)}$ are typical of complexes including weakly bound solvent ligands, such as acetone.[7] The low temperature ^{1}H NMR spectrum shows many NBD and aromatic environments, as expected with multiple isomers, however, a potential aryl anagostic interaction was observed at 9.16 ppm as a doublet of doublets, suggestive of a Rh(I) square planar arrangement.

^{31}P{^{1}H} NMR (162 MHz, acetone-D$_6$, 298 K): δ 44.7 (dd, J_{RhP} = 168 Hz and J_{PP} = 27 Hz), 36.6 (br multiplet) and 31.6 (br multiplet).

^{31}P {^{1}H} NMR (162 MHz, acetone-D$_6$, 193 K): δ 44.3 (complex multiplet), 37.5 (dd, J_{RhP} = 223 Hz and J_{PP} = 58 Hz), 36.2 (d, J_{RhP} = 213 Hz), 33.2 (dd, J_{RhP} = 220 Hz and J_{PP} = 58 Hz), 30.3 (dd, J_{RhP} = 205 Hz and J_{PP} = 58 Hz), 29.4 (dd, J_{RhP} = 210 Hz and J_{PP} = 58 Hz) and 19.7 (complex multiplet).

^{1}H NMR (400 MHz, acetone-D$_6$, 298 K): δ 8.09 (complex multiplet, 2H, Ar), 7.79 (8H, *o*-C*H* BAr$^F{}_4$), 7.67 (s, 4H, *p*-C*H* BAr$^F{}_4$), 7.64-6.26 (complex m, 22H, Ar), 3.69 (complex multiplet, 12H, OC*H*$_3$).

^{1}H NMR (400 MHz, acetone-D$_6$, 193 K) selected data: δ 9.16 (dd, 1H, anagostic Ar-*H*).

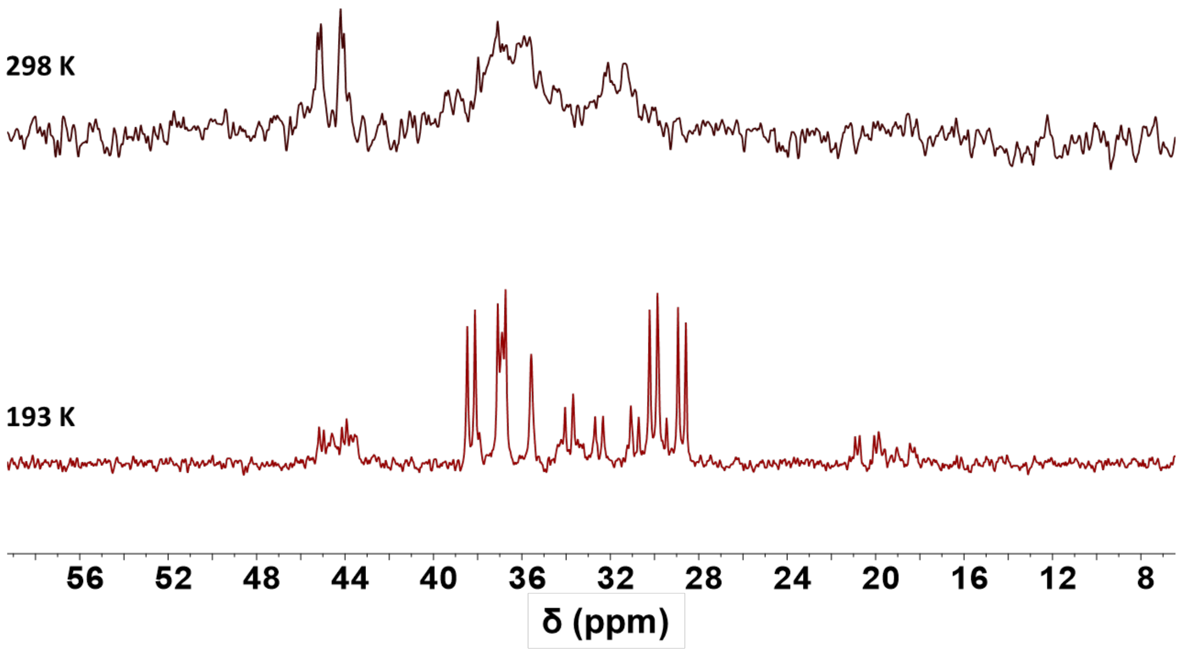

Figure S51. $^{31}P\{^1H\}$ NMR spectrum of **3-OMe** at 298 K and 193 K (acetone-D_6, 162 MHz).

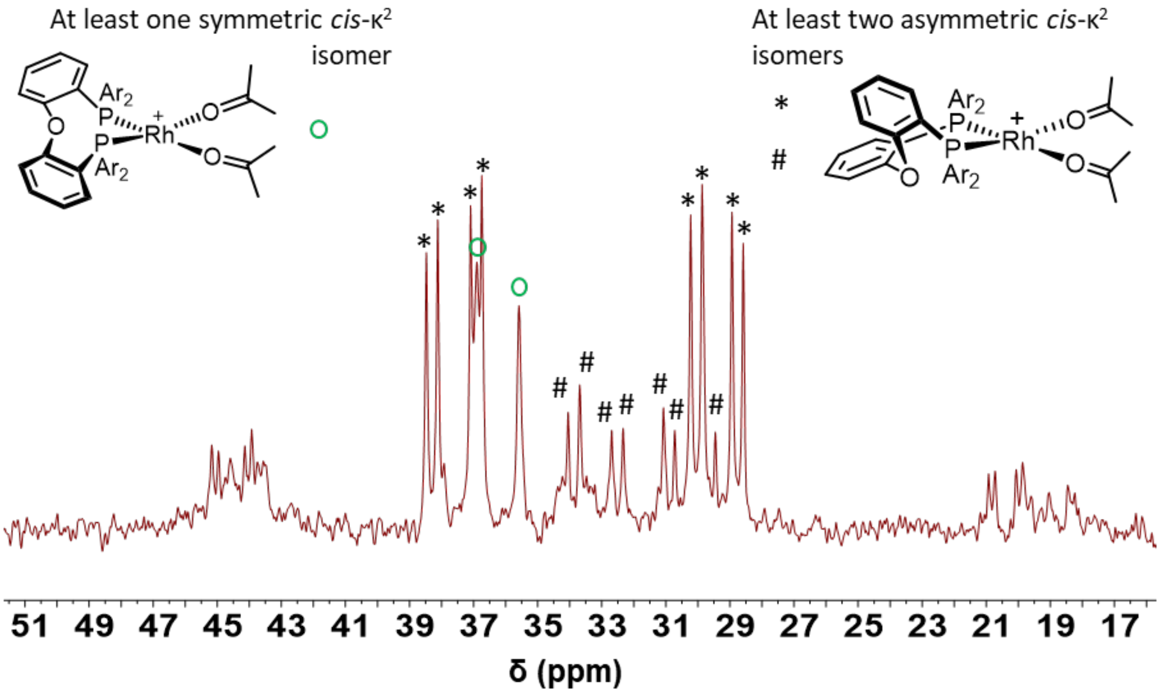

Figure S52. Low temperature (193 K) $^{31}P\ \{^1H\}$ NMR spectrum of **3-OMe** tentatively assigned as two isomers with a *cis*-κ^2 ligand arrangement, one *mer*-κ^3 isomer and another unknown species.

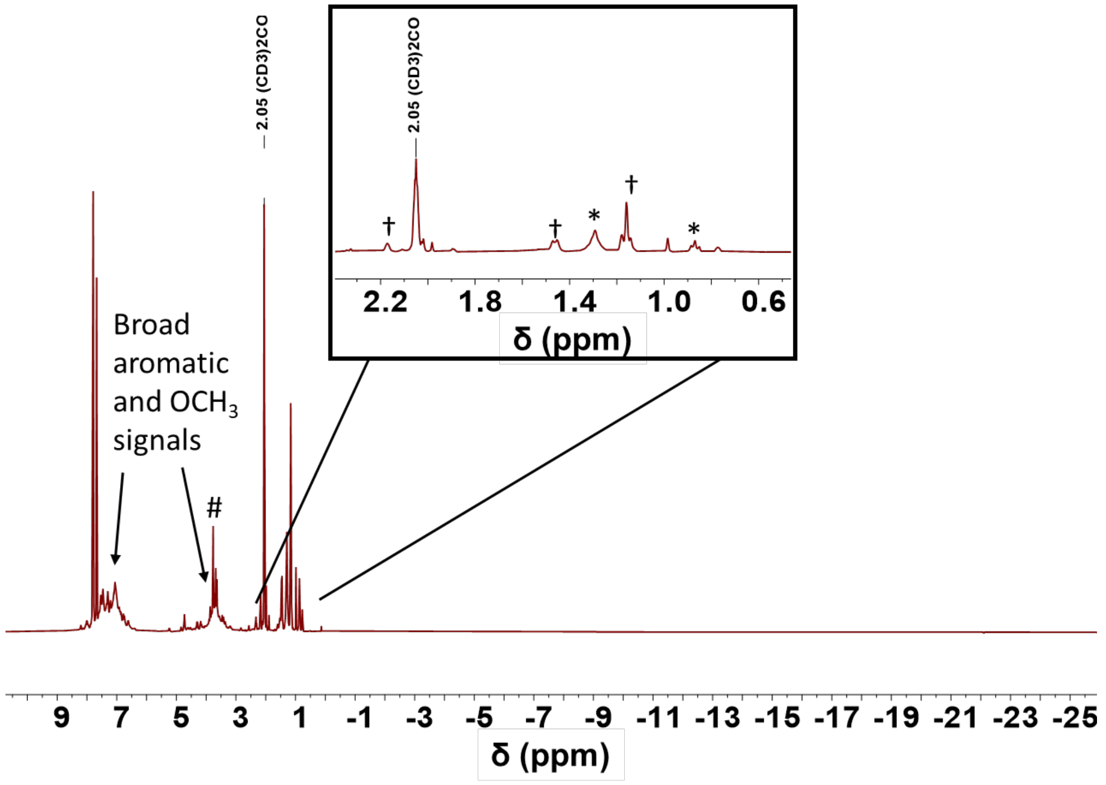

Figure S53. ^{1}H NMR spectrum of acetone bound species **3-OMe** (acetone-D6, 400 MHz, 298 K). † Denotes free NBA, * shows pentane impurity and # is 2-propanol from hydrogenation of acetone-D6. There are no observable hydrides.

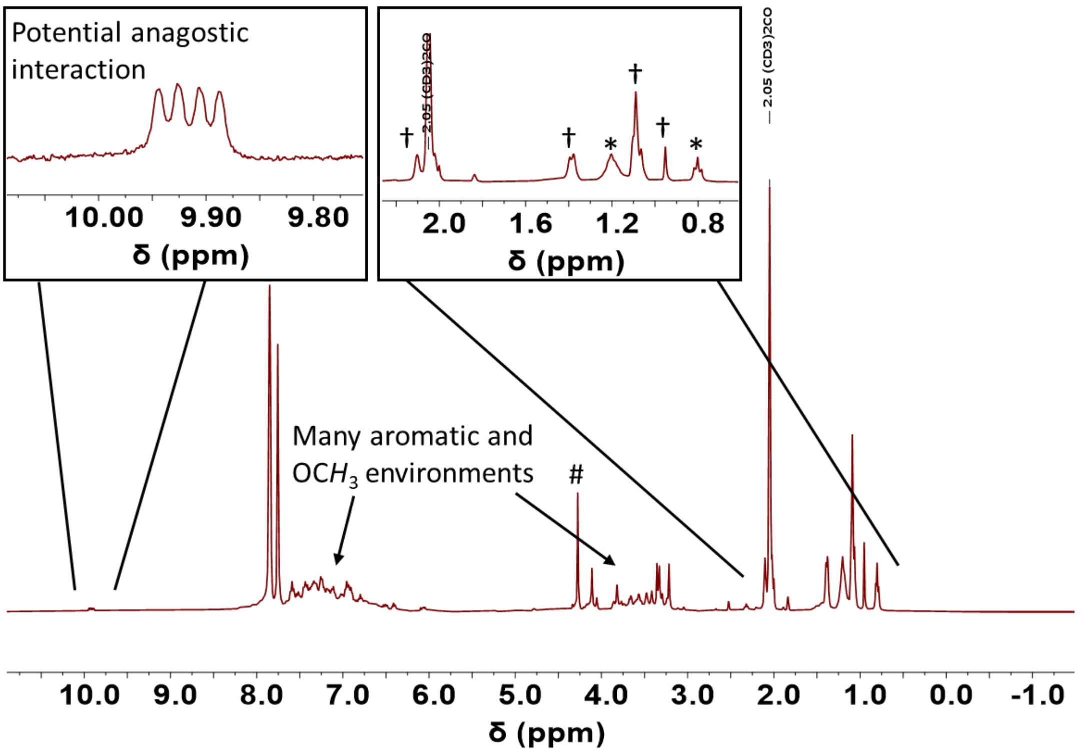

Figure S54. ^{1}H NMR spectrum of acetone bound species **3-OMe** (acetone-D6, 400 MHz, 193 K). † Denotes free NBA, * shows pentane impurity and # is 2-propanol from hydrogenation of acetone-D6.

Formation of 4-^{i}Pr

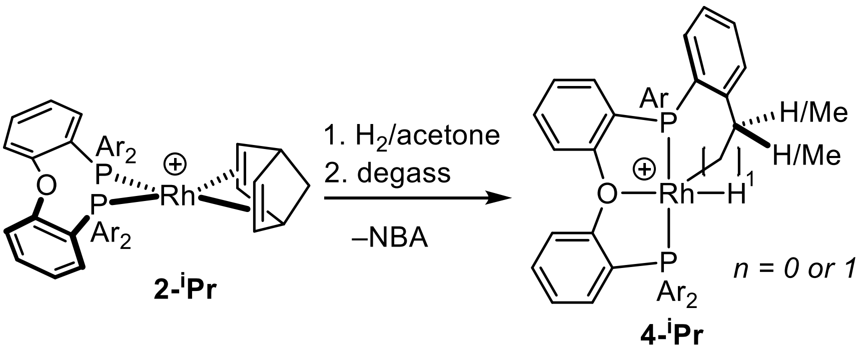

4-^{i}Pr

Scheme S5. Formation of **4-^{i}Pr** from hydrogenation of **2-^{i}Pr**. $[BAr^{F}_{4}]^{-}$ anions omitted for clarity.

4-^{i}Pr is characterised as a Rh(III) cyclometallated species in which a low energy barrier fluxional process is occurring. At room temperature the $^{31}P\{^{1}H\}$ NMR spectrum is broad but cooling the sample to 183 K reveals multiple isomers of a Rh(III) species, as shown by small $J_{Rh(III)P}$ of 112-122 Hz. A large *trans* J_{PP} (357-362 Hz) also suggests a *meridonal* arrangement of the **1-^{i}Pr** ligand. The hydride signal observed at room temperature in the ^{1}H NMR spectrum contains coupling to two chemically equivalent ^{31}P nuclei (J_{PH} =15 Hz) as well as J_{RhH} (29 Hz), suggesting a single hydride environment coupling to two *cis* ^{31}P nuclei. Upon cooling to 243 K, the hydride signal broadens and then further cooling to 183 K reveals multiple hydride resonances each comprising J_{PH} and J_{RhH}, in coherence with the $^{31}P\{^{1}H\}$ NMR spectroscopy. This suggests a fluxionality that does not involve breaking of the Rh-H bond. Broad aromatic and *iso*-propyl signals do not elucidate any structural information but free NBA suggests hydrogenation of the NBD fragment. The low temperature NOESY NMR spectrum supports formation of a cyclometallated structure (and not a dihydride) as there are observable NOE interactions between the three hydride signals and a total of nine methyl and methine protons (2 x coordinated methyl and 1 x methine for each isomer) which are in close proximity to the hydride (< 5 Å).

$^{31}P\{^{1}H\}$ NMR (162 MHz, acetone-D$_6$, 298 K): δ 20.8 (br s).

$^{31}P\{^{1}H\}$ NMR (162 MHz, acetone-D$_6$, 183 K): δ 39.7 (dd, J_{RhP} = 121 Hz and J_{PP} = 357 Hz, isomer **a**), 26.1 (complex multiplet that contains the second ^{31}P environment for the three major isomers), 14.0 (dd, J_{RhP} = 119 Hz and J_{PP} = 359 Hz, isomer **b**) and 5.6 (dd, J_{RhP} = 112 Hz and J_{PP} = 362 Hz, isomer **c**).

^{1}H NMR (400 MHz, acetone-D$_6$, 298 K): δ 7.99 (multiplet, 2H, Ar), 7.79 (8H, *o*-CH BAr^{F}_{4}), 7.67 (s, 4H, *p*-CH BAr^{F}_{4}), 7.64-6.44 (complex m, 22H, Ar), 3.37 (br s, 4H, methine C*H*), 1.51-0.38 (br s, 24H, CH_3) and -19.81 (dvt, J_{RhH} = 29 Hz and J_{PH} = 15 Hz).

^{1}H NMR (400 MHz, acetone-D$_6$, 183 K): δ 8.27 (multiplet, 1H, Ar), 8.04 (multiplet, 1H, Ar), 7.89 (8H, *o*-CH BAr^{F}_{4}), 7.77 (s, 4H, *p*-CH BAr^{F}_{4}), 7.74-6.18 (complex m, 22H, Ar), 3.37 (br s, 4H, methine C*H*), 1.51-0.38 (br s, 24H, CH_3). There are six signal that could be methine CH signals, all broad singlets at 4.80, 3.65, 3.34, 3.21, 3.10, 2.72 ppm. There are many signals (> 10) in the alkyl region (2-38-0.08 ppm) that overlap with other signals so they cannot be fully assigned but most likely the *iso*-propyl CH_3 groups. Multiple hydride resonances are observed overlapping from -19.40 to -19.95 ppm that integrate to one proton overall.

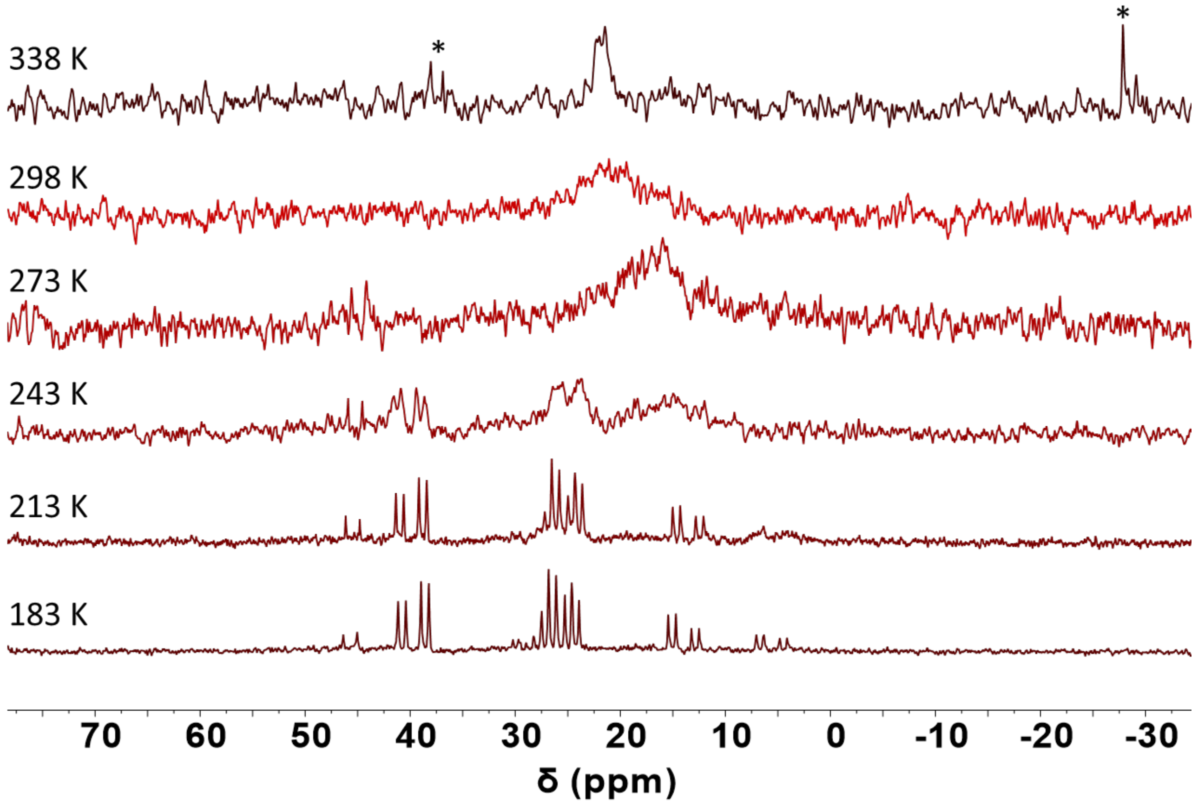

Figure S55. Variable temperature $^{31}P\{^{1}H\}$ NMR spectra of **4-^{i}Pr** formed in situ (acetone-D$_6$, 162 MHz). * Denotes unknown decomposition product.

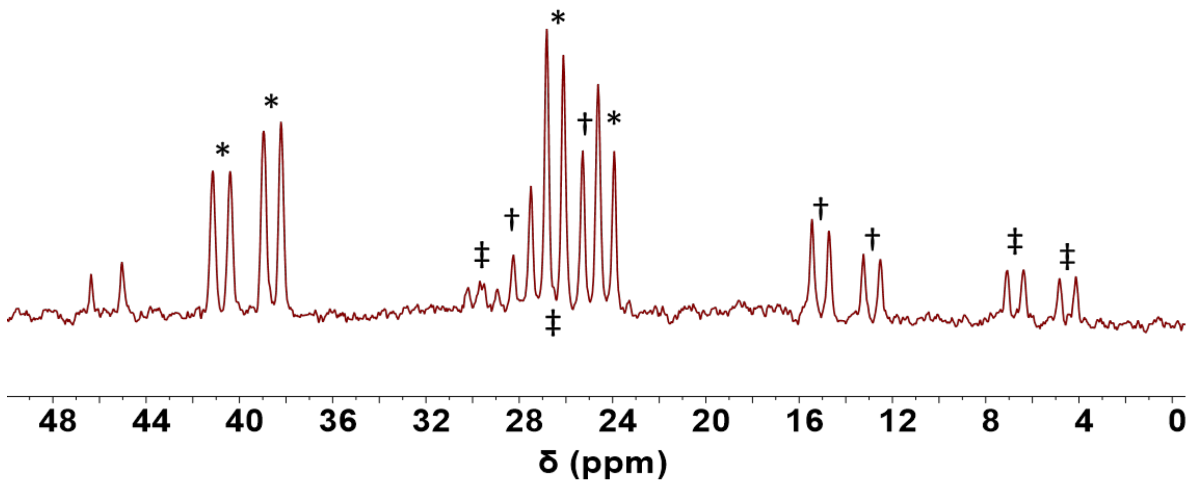

Figure S56. $^{31}P\{^{1}H\}$ NMR of **4-^{i}Pr** with assignments of the three main isomers with *, † and ‡ respectively (acetone-D$_6$, 162 MHz, 183 K).

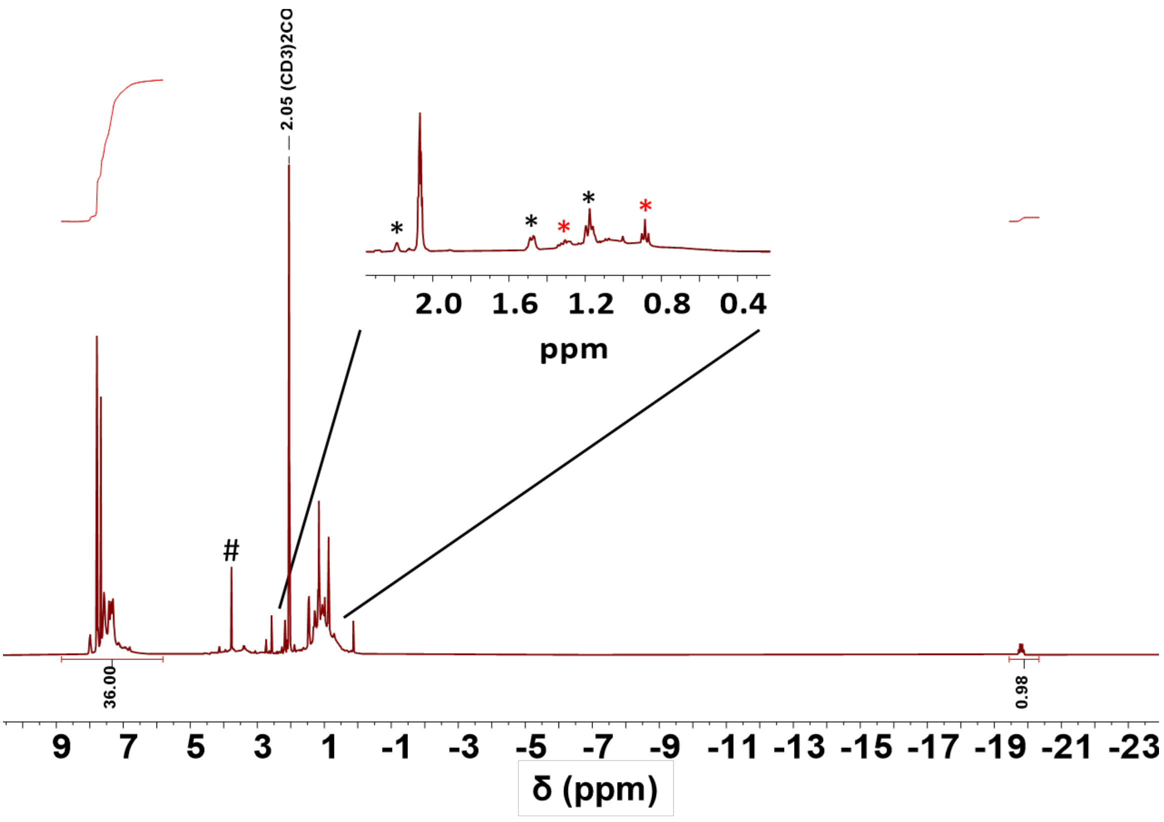

Figure S57. ^{1}H NMR spectrum of **4-[i]Pr** after degassing by five successive freeze-pump-thaws and put under an atmosphere of argon (acetone-D_6, 400 MHz, 298 K). * Denotes pentane impurity, † is free NBA and # is 2-propanol formed from hydrogenation of acetone-D_6. Integral relative to all the aromatic signals.

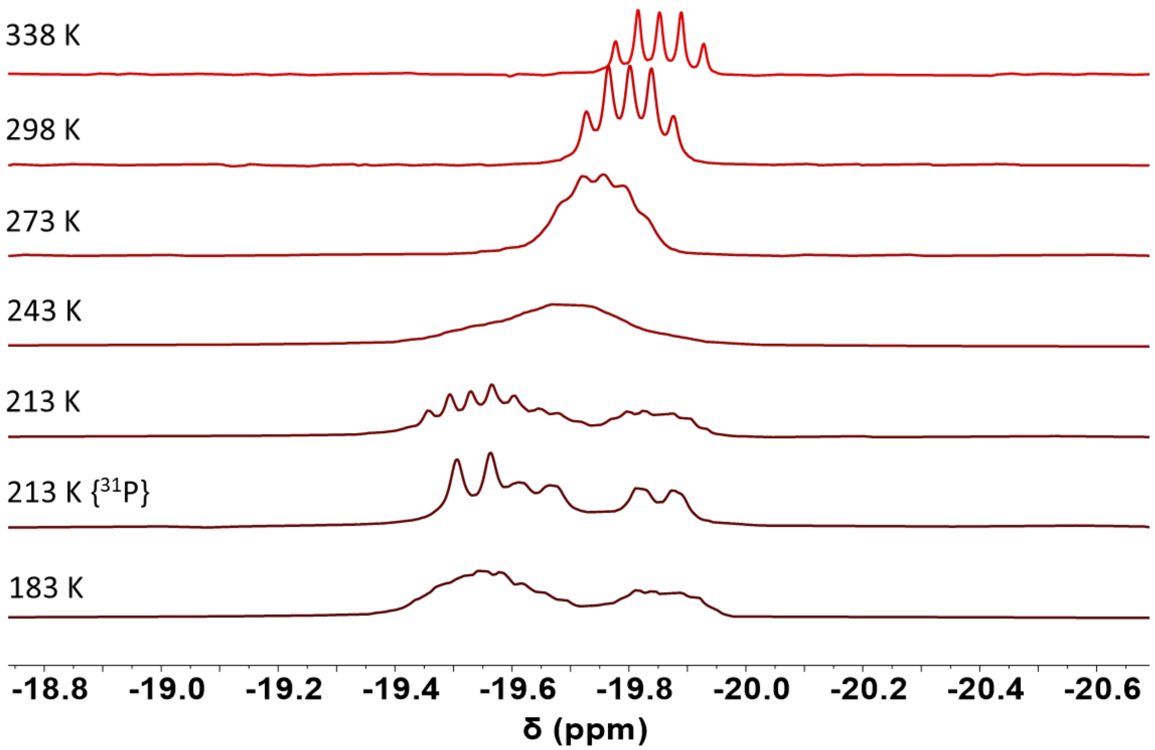

Figure S58. Variable temperature ^{1}H NMR (unless specified) spectra of the hydride signal in **4-[i]Pr** (acetone-D_6, 400 MHz).

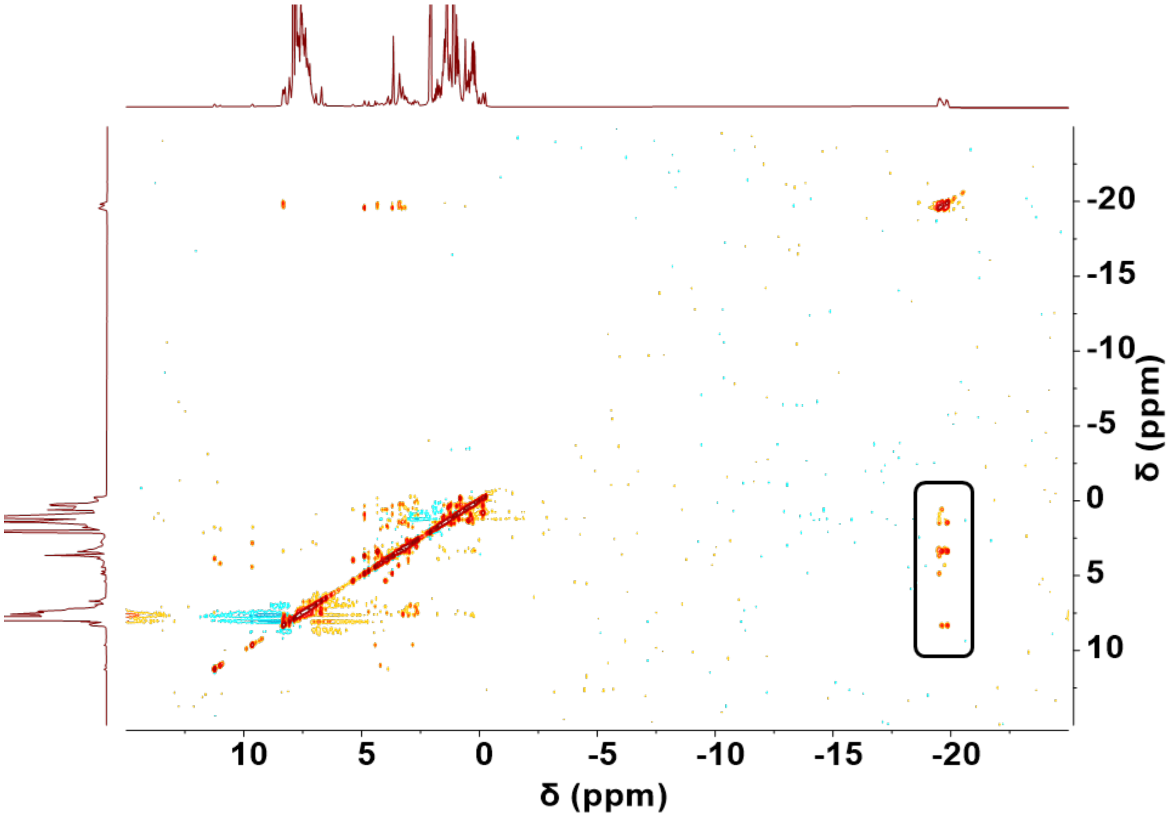

Figure S59. 1H/1H NOESY spectrum of **4-^{i}Pr** (500/500 MHz, acetone-D$_6$, 183 K). Region highlighted shows interactions between the hydride signals and alkyl and aryl protons that are within close proximity (< 5 Å).

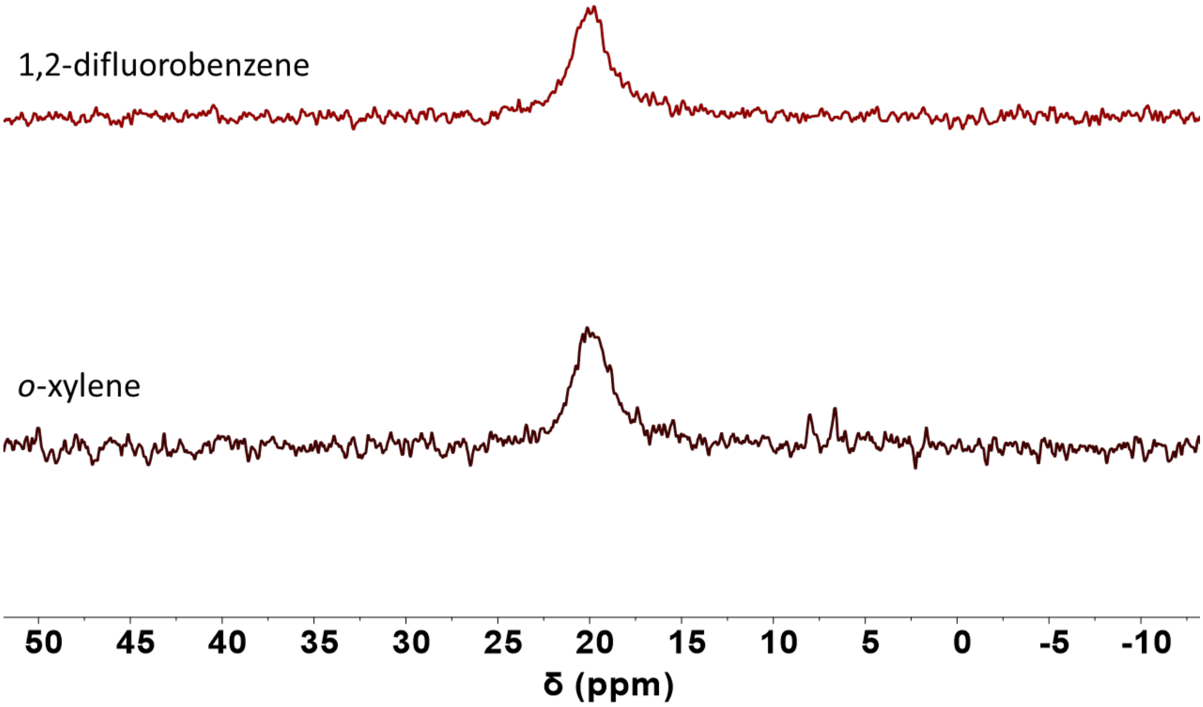

Figure S60. ^{31}P{^{1}H} NMR spectra of **4-^{i}Pr** formed in-situ by addition of 2 bar H_2 to **4-^{i}Pr** in 1,2-difluorobenzene or *o*-xylene solvents (162 MHz, 298 K).

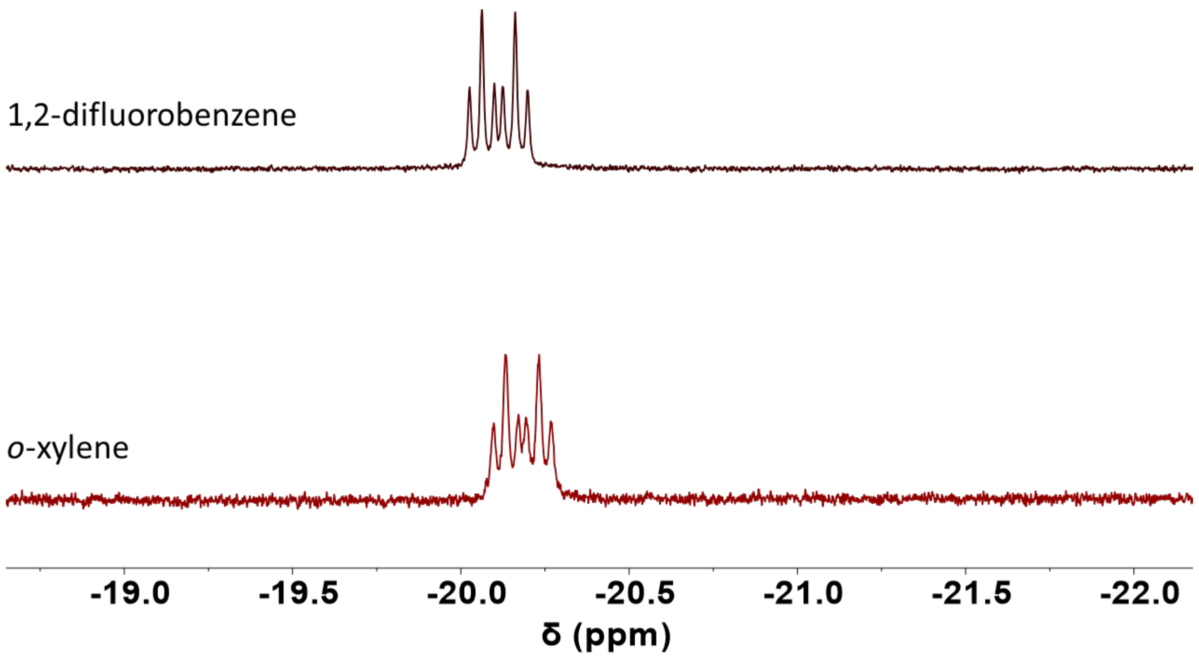

Figure S61. Selected hydride region of the ^{1}H NMR spectra of **4-^{i}Pr** formed in-situ by addition of 2 bar H_2 to **2-^{i}Pr** in 1,2-difluorobenzene or *o*-xylene solvents (400 MHz, 298 K).

Reformation of 2-^{i}Pr by addition of NBD to 4-^{i}Pr

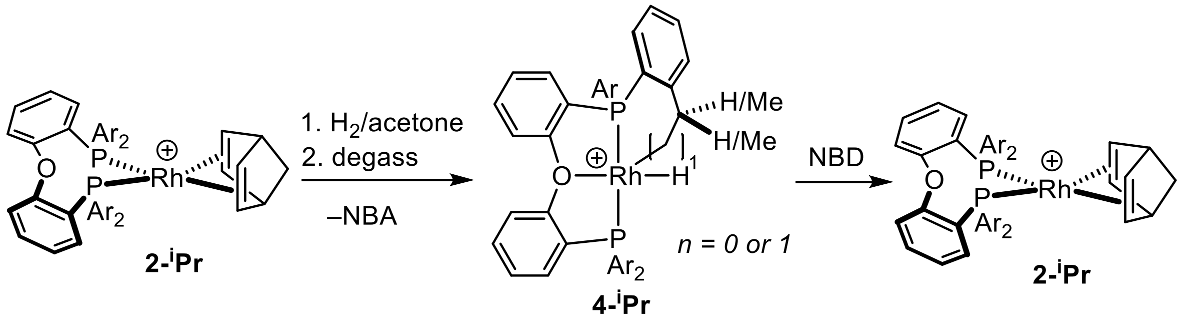

Scheme S6. Reformation of **2-^{i}Pr** by the addition of NBD to **4-^{i}Pr** made in-situ.

A sample of **2-^{i}Pr** (20 mg) was dissolved in acetone-D$_6$ (0.5 ml) in a high-pressure J. Youngs NMR tube and was degassed by three successive freeze-pump-thaws. The NMR tube was put under an atmosphere of 2 bar H_2, shaken and left for 30 minutes as the colour changed from orange to yellow. Formation of **4-^{i}Pr** was confirmed by in-situ ^{31}P and ^{1}H NMR spectroscopy. The H_2 was removed from the NMR tube (six successive free-pump-thaws) then refilled with argon. NBD (0.1 ml) was added to the yellow solution that immediately turned orange as **2-^{i}Pr** was reformed, confirmed by ^{31}P{^{1}H} and ^{1}H NMR spectroscopy. The characteristic downfield shifted *ortho*-proton in **2-^{i}Pr** at 9.34 ppm is observed after NBD addition as the hydride signal at -19.81 ppm is no longer observable.

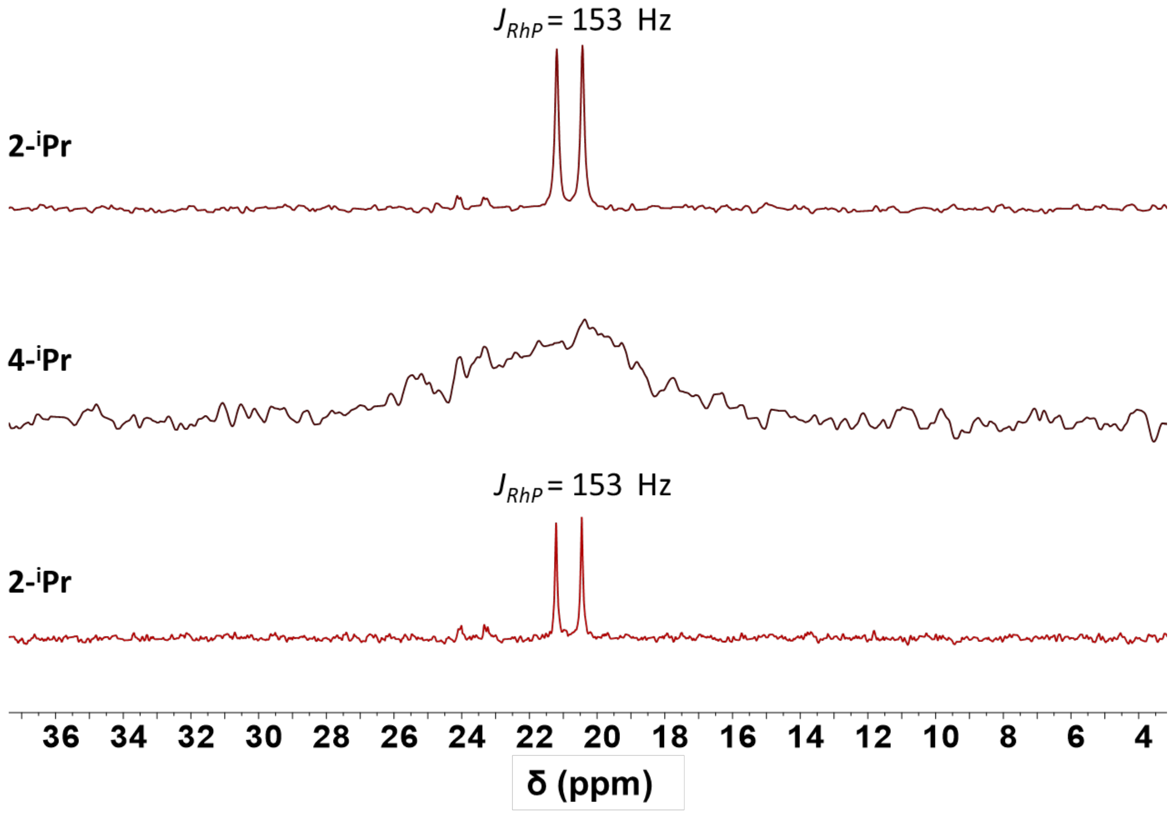

Figure S62. ^{31}P{^{1}H} NMR spectra of **2-^{i}Pr** before addition of H_2 (top), after 30 minutes under 2 bar H_2 (**4-^{i}Pr**) (middle) and after addition of NBD (bottom) (202 MHz, acetone-D$_6$, 298 K).

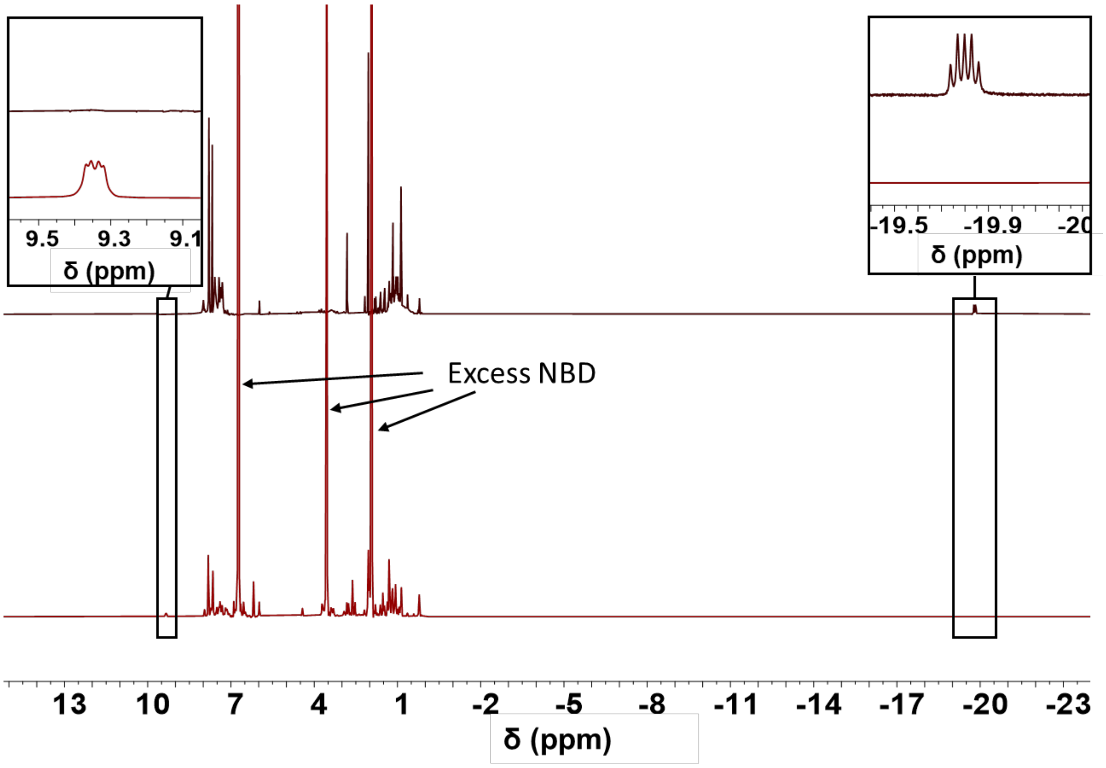

Figure S63. ^{1}H NMR spectra of **4-^{i}Pr** (top) and the same sample after addition of NBD to reform **2-^{i}Pr** (bottom) (500 MHz, acetone-D_6, 298 K).

Deuterium Incorporation into 4-^{i}Pr

A sample of **2-^{i}Pr** was dissolved in *o*-xylene solvent in a high-pressure J. Youngs NMR tube and was degassed (by three successive freeze-pump-thaws) and put under an atmosphere of 2 bar H_2, shaken and left for 30 minutes which formed 4-**^{i}Pr**. This sample was then degassed (three successive freeze-pump-thaws) and put under an atmosphere of 2 bar D_2 for 2 weeks (including three recharges) to form an isotopologue of **4-^{i}Pr, 4-^{i}Pr-D$_x$**. NBD was added (100 eq.) which resulted in an instant colour change from yellow to orange and the reformation of the NBD complex, **2-^{i}Pr-D$_x$**, which was confirmed by $^{31}P\{^1H\}$ NMR spectroscopy. The solvent was removed *in vacuo* and the resulting orange oil was washed with pentane and the resulting orange powder was dried over Schlenk line vacuum overnight. A sample of this solid was analysed by ESI-MS and the deuterium isotope distribution was determined by use of a purpose-built python script to find the solution to a linear matrix equation, **AX=B**. In which, **A** is a matrix describing the isotopic pattern at each m/z, **B** a matrix giving the intensity of peaks in the experimental mass spectrum, and **X** the distribution of deuterium incorporation which was determined by least-squares regression. Python 3 was run through the anaconda software distribution and regression analysis carried out using the SciPy packages.[9-11]

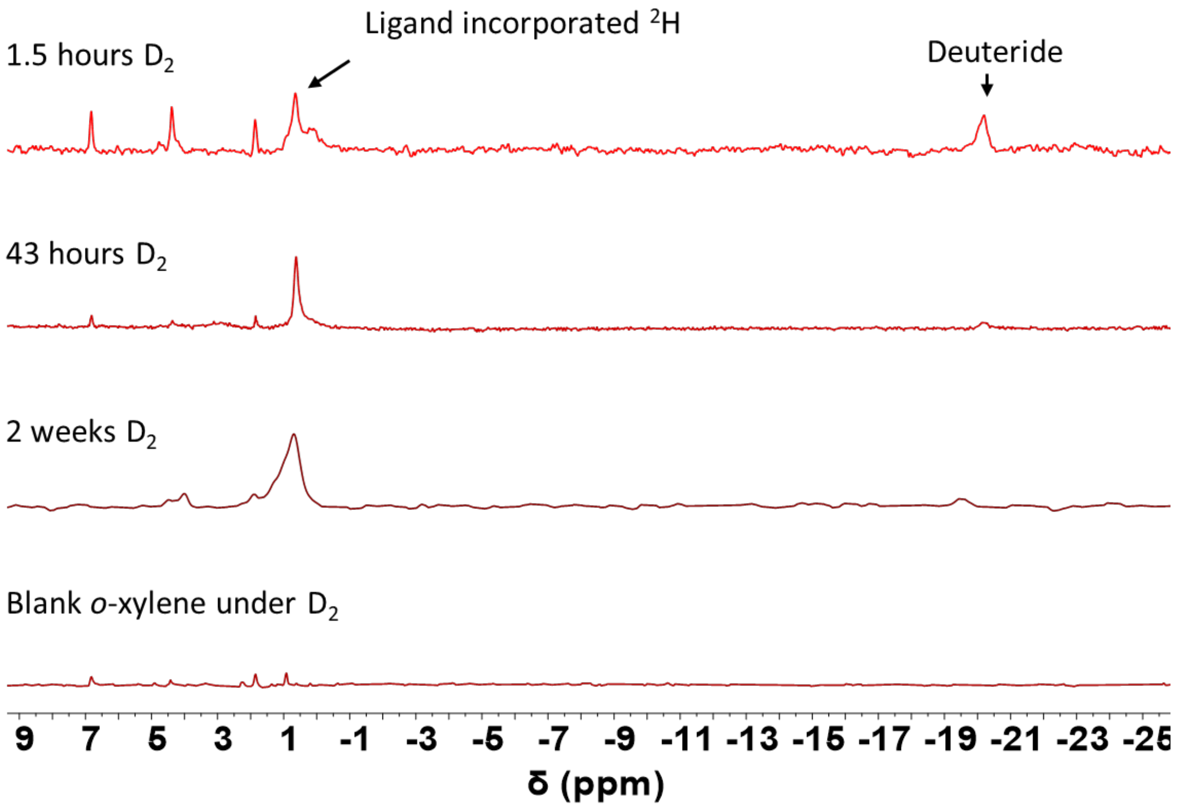

Figure S64. ^{2}H NMR spectra of a sample of **4-^{i}Pr-D$_x$** under an atmosphere of 2 bar D_2 for 1.5 hours, 43 hours and two weeks compared to blank sample of *o*-xylene also under 2 bar D_2 (61 MHz, *o*-xylene, 298 K).

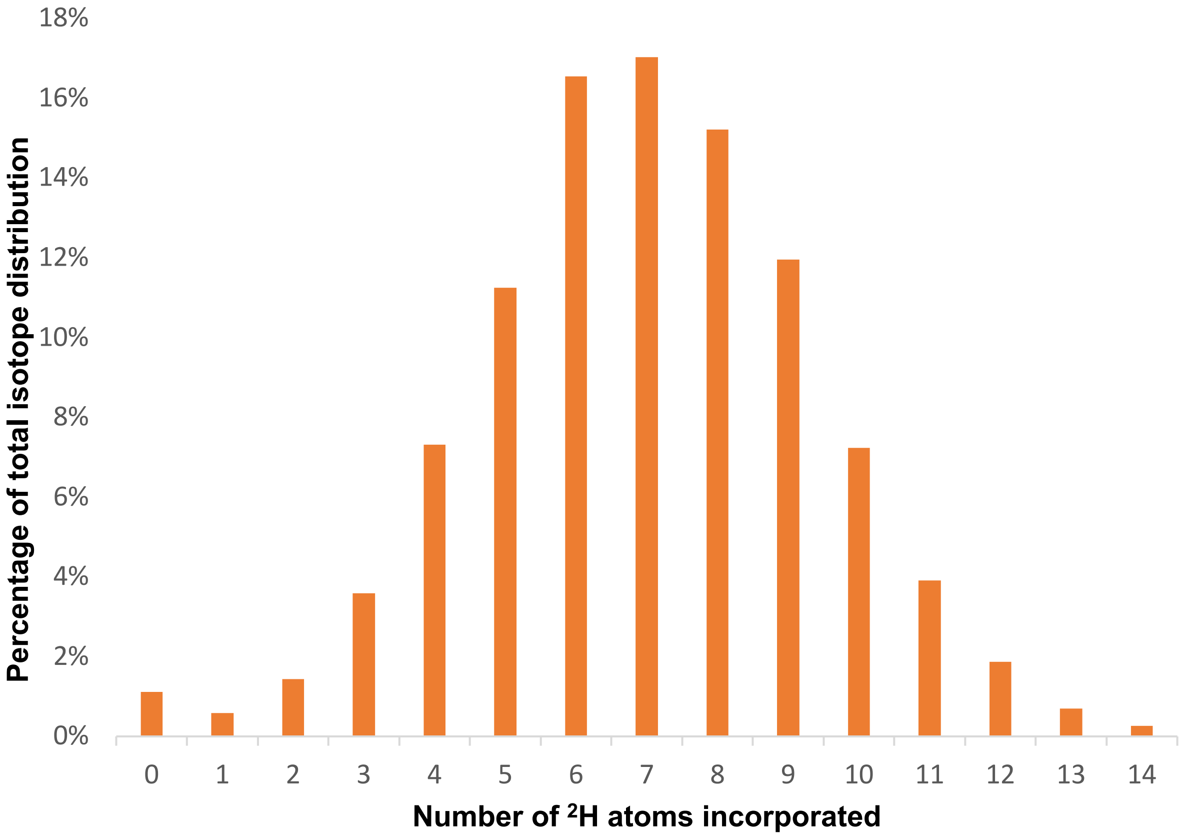

Figure S65. Deuterium isotope distribution calculated via a python script using ESI-MS data of **2-^{i}Pr-D$_{x}$**.

Formation of [Rh(κ^3-P,O,P-DPEphos-^{i}Pr)(CO)][Bar$^F{}_4$] 5-^{i}Pr

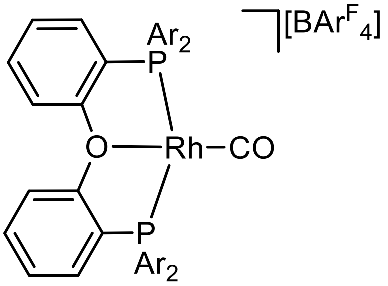

A sample of **2-^{i}Pr** (20 mg, 0.008 mmol) in 1,2-difluorobenzene (0.5 ml) was put under an atmosphere of 2 bar H_2 and left for 30 minutes. The H_2 was removed via three freeze-pump-thaws and the sample was put under an atmosphere of 2 Bar CO resulting in an immediate colour change from yellow to orange. The CO was removed via three times freeze-pump-thaws and the sample refilled with argon. The solution was transferred to a small J. Youngs flask then layered with pentane and kept at -20 ºC yielding orange crystals suitable for single crystal x-ray crystallography after two days that were separated via filtration and dried under Schlenk line vacuum (< 1 x 10^{-1} mbar) overnight (9 mg, 0.005 mmol, 63%).

^{31}P{^{1}H} NMR (162 MHz, acetone-D$_6$, 298 K): δ 19.9 (d, J_{RhP} = 123 Hz).

^{1}H NMR (500 MHz, acetone-D$_6$, 298 K): δ 7.97-7.55 (complex m, 25H, *o*-C*H* BAr$^F{}_4$ (8H), *p*-C*H* BAr$^F{}_4$ (4H) and Ar (13H)), 7.53-7.21 (m, 10H, Ar), 7.02 (br s, 1H, Ar), 4.74 (br s, 2H, methine C*H*) 3.10 (br s, 2H, methine C*H*), 1.49 (br s, 6H, CH_3), 1.17 (br s, 12H, CH_3), 0.40 (br s, 6H CH_3).

^{1}H NMR (500 MHz, acetone-D$_6$, 183 K): selected data δ 1.41 (d, J_{HH} = 4 Hz, 6H, CH_3), 1.19 (d, J_{HH} = 4 Hz, 6H, CH_3), 1.00 (d, J_{HH} = 4 Hz, 6H, CH_3), 0.14 (d, J_{HH} = 5 Hz, 6H, CH_3).

ESI-MS (1,2-difluorobenzene): *m/z* [M]$^+$ 837.249 (Calc. 837.250) with the correct isotope pattern.

Elemental analysis found (calc. for $C_{87}H_{72}BF_{24}OP_2Rh$): C 56.73 (57.19) H 3.73 (3.79).

IR (CO stretch): 2023 cm^{-1}

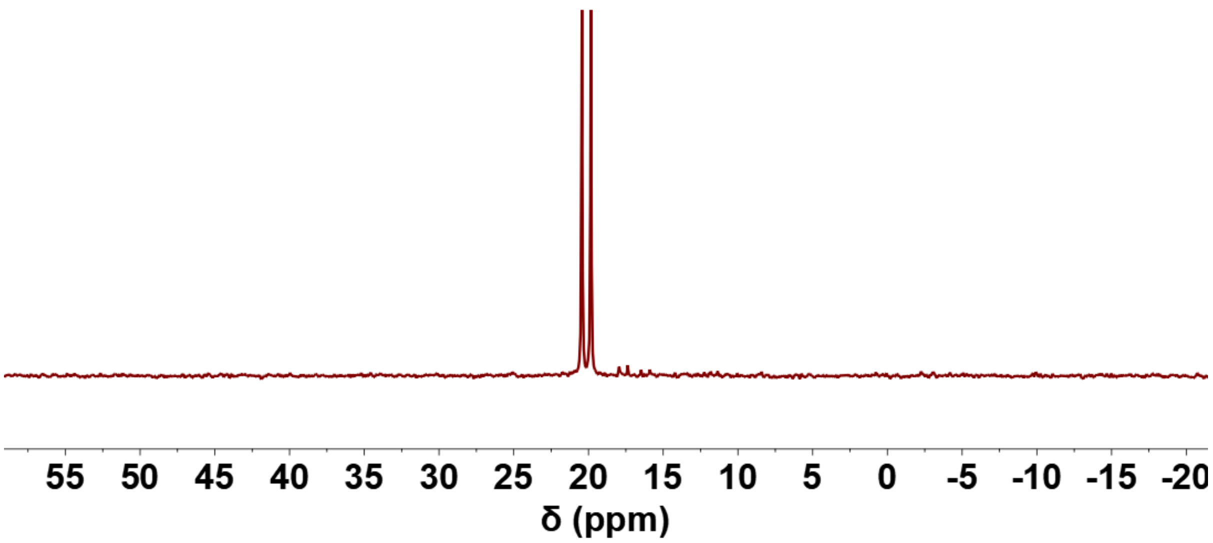

Figure S66. $^{31}P\{^{1}H\}$ NMR spectrum of **5-^{i}Pr** (acetone-D$_6$, 400 MHz, 298 K).

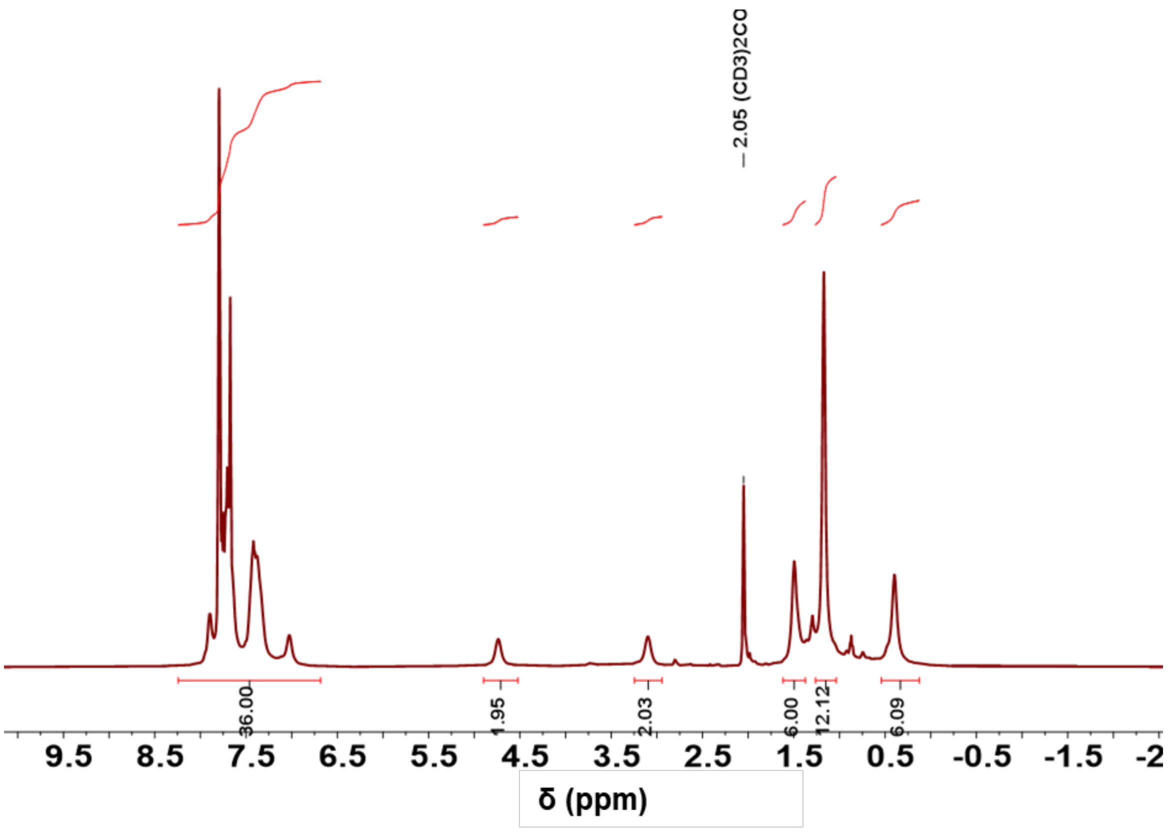

Figure S67. ^{1}H NMR of **5-^{i}Pr** (acetone-D$_6$, 500 MHz, 298 K). Integrals relative to the total aromatic peaks in both the cation and anion.

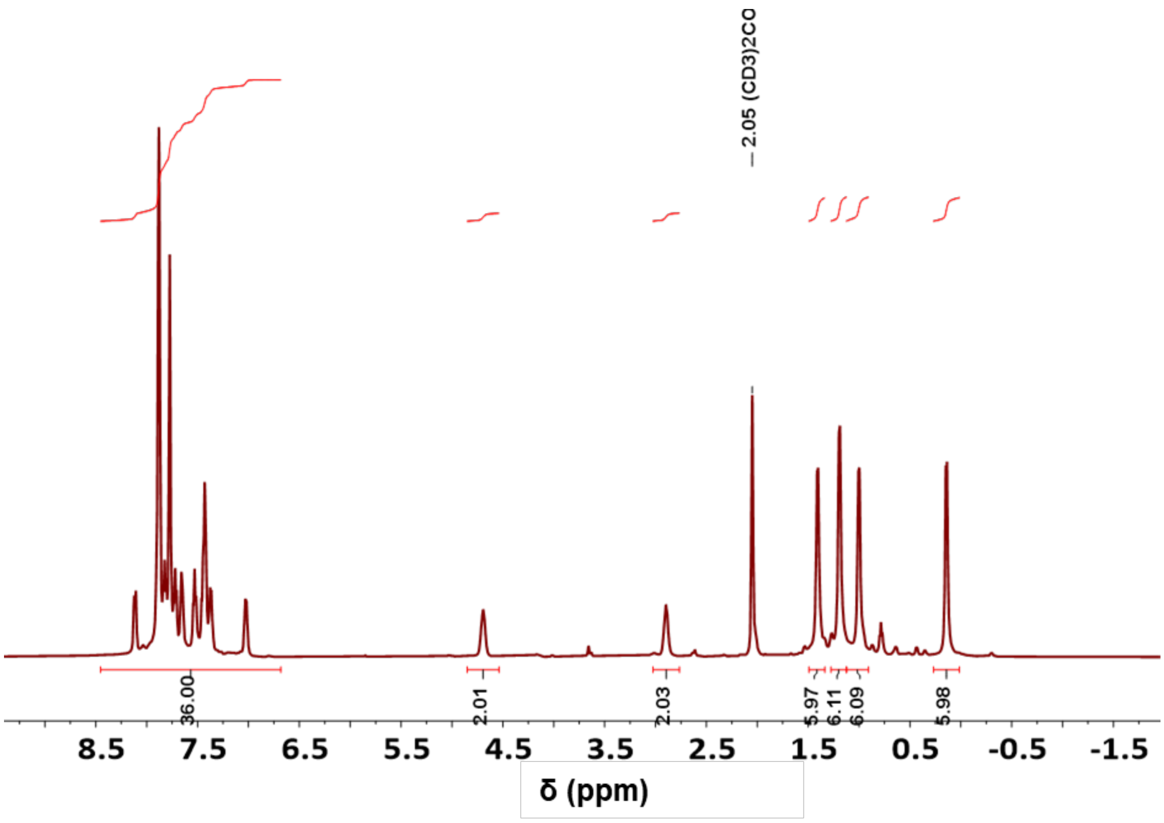

Figure S68. ^{1}H NMR of **5-[i]Pr** (acetone-D_6, 500 MHz, 183 K). Integrals relative to the total aromatic peaks in both the cation and anion.

Formation of [Rh(κ^2-P,P-(DPEphos-^{i}Pr'-η^1-BH_2NMe_3)][BAr$^F_4$] 6-^{i}Pr

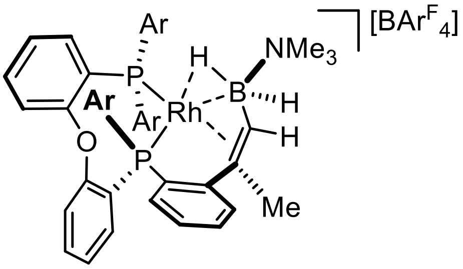

A sample of **2-^{i}Pr** (30 mg, 0.017 mmol) in 1,2-difluorobenzene was put under an atmosphere of 2 bar H_2 and left for 30 minutes. The sample was degassed by three successive freeze-pump-thaws and the sample put under an atmosphere of argon. $H_3B{\cdot}NMe_3$ (37 mg, 0.051 mmol, 3 equiv.) in 1,2-difluorobenzene (0.5 ml) was added to the yellow solution which formed a blue solution. 3,3-dimethylbutene (11 µl, 0.085 mmol, 5 equiv.) was then added and the solution was left for seven days with occasional shaking. The solvent was mostly removed *in vacuo* and then pentane was added to crash out a green precipitate. The solid was isolated by filtration and dried under Schlenk line vacuum (<1 x 10^{-1} mbar) to yield a green solid (27.1 mg, 0.015 mmol, 88% yield). Crystals suitable for x-ray diffraction were obtained by dissolving in minimal 1,2-difluorobenzene and layering with pentane.

^{31}P{^{1}H} NMR (162 MHz, CD_2Cl_2, 298 K): δ 29.5 (dd, J_{RhP} = 187 Hz and J_{PP} = 31 Hz) and 8.5 (dd, J_{RhP} = 165 Hz and J_{PP} = 32 Hz)

^{1}H NMR (500 MHz, CD_2Cl_2, 298 K): δ 7.93 (dd, *J* = 7 Hz and 14 Hz, 1H, Ar) 7.72 (s, 8H, *o*-CH BAr$^F_4$), 7.56 (s, 4H, *p*-CH BAr$^F_4$), 7.60-6.94 (complex m, 16H Ar), 6.82 (m, 2H, Ar), 6.65 (m, 3H, Ar), 6.46 (dd, *J* = 8 and 11 Hz, 1H, Ar), 6.36 (dd, *J* = 8 and 11 Hz, 1H, Ar), 4.92 (m, 1H, anagostic methine C*H*), 4.75 (m, 1H, anagostic methine C*H*), 3.86 (s, 1H, C=C-*H*), 2.48 (s, 9H, N(CH_3)$_3$), 2.32 (m, 1H, methine C*H*), 1.72 (s, 3H, C=C-CH_3), 1.24 (d, J_{HH} = 6 Hz, 3H, CH_3), 1.09 (d, J_{HH} = 6 Hz, 3H, CH_3), 0.83 (d, J_{HH} = 7 Hz, 3H, CH_3) 0.81 (d, J_{HH} = 7 Hz, 3H, CH_3), 0.76 (d, J_{HH} = 6 Hz, 3H, CH_3), 0.30 (d, J_{HH} = 7 Hz, 3H, CH_3) and -7.54 (br s, 1H, agostic B-*H*). Second B*H* signal was not observable without ^{11}B decoupling due to the quadrupolar nature of the bonded B atom.

^{1}H{^{11}B} NMR (600 MHz, CD_2Cl_2, 298 K) selected data: δ 3.86 (s, 1H, C=C-*H*), 1.89 (br d, $^2J_{HH}$ = 14 Hz, 1H, B*H*) and -7.54 (ddd, $J_{PP(trans)}$ = 52 Hz, $^2J_{HH}$ = 14 Hz, J_{RhH} = 14 Hz, 1H, agostic B-*H*).

^{11}B NMR (128 MHz, CD_2Cl_2, 298 K): δ -6.3 to -14.1 (br s, *B*H_2) and -9.1 (s, BAr$^F_4$)

ESI-MS (1,2-difluorobenzene): *m/z* [M]$^+$ 878.33 (Calc. 878.33) with the correct isotope pattern.

Multiple samples were submitted for elemental analysis, but no results were within 0.4% of the theoretical percentage mass for carbon, hydrogen, or nitrogen. Persistent pentane after recrystallisation may be the cause of inconsistent elemental analysis; see ^{1}H, ^{31}P and ^{11}B NMR spectroscopy data below for evidence of purity otherwise.

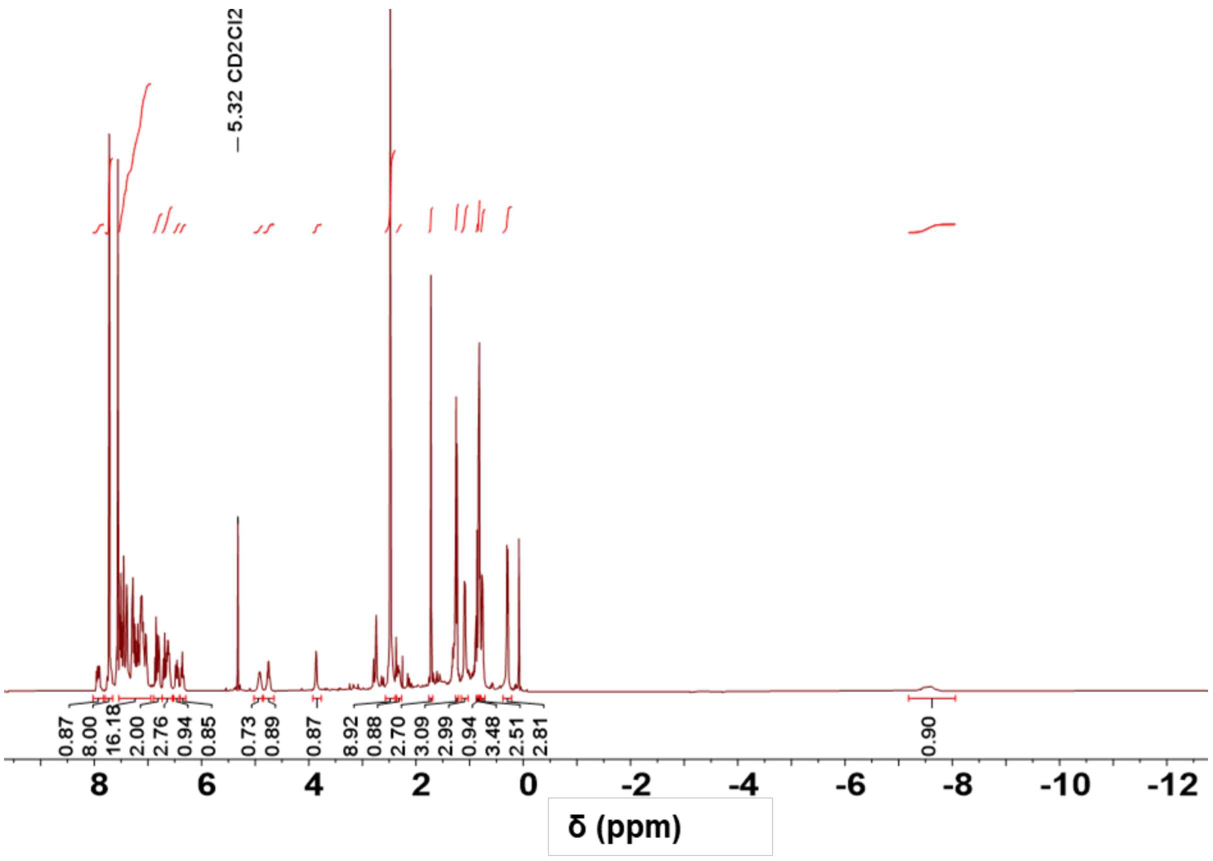

Figure S69. ^{1}H NMR spectrum of **6-^{i}Pr** (CD_2Cl_2, 400 MHz, 298 K).

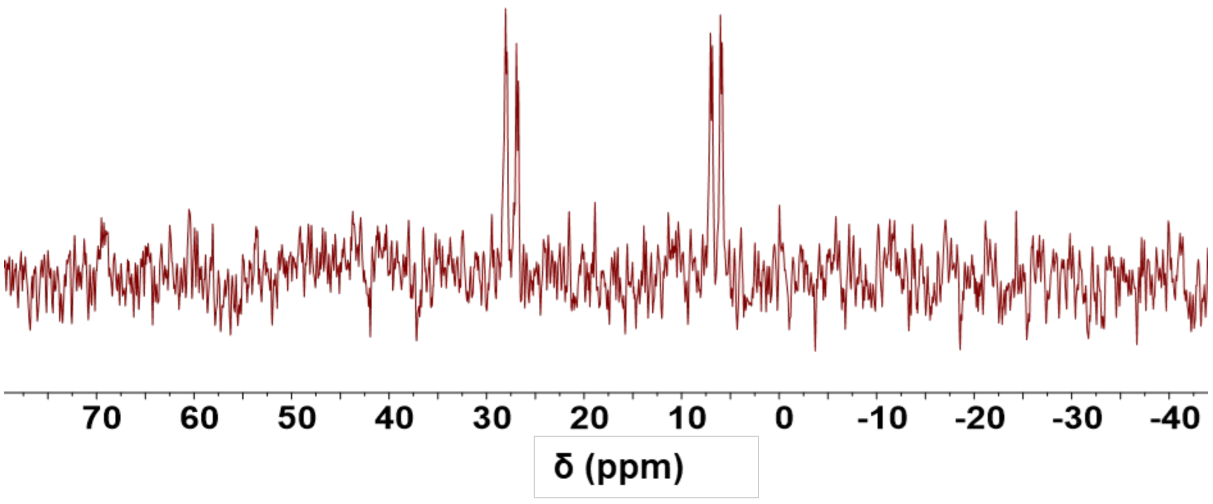

Figure S70. $^{31}P\{^{1}H\}$ NMR spectrum of **6-^{i}Pr** (CD_2Cl_2, 162 MHz, 298 K).

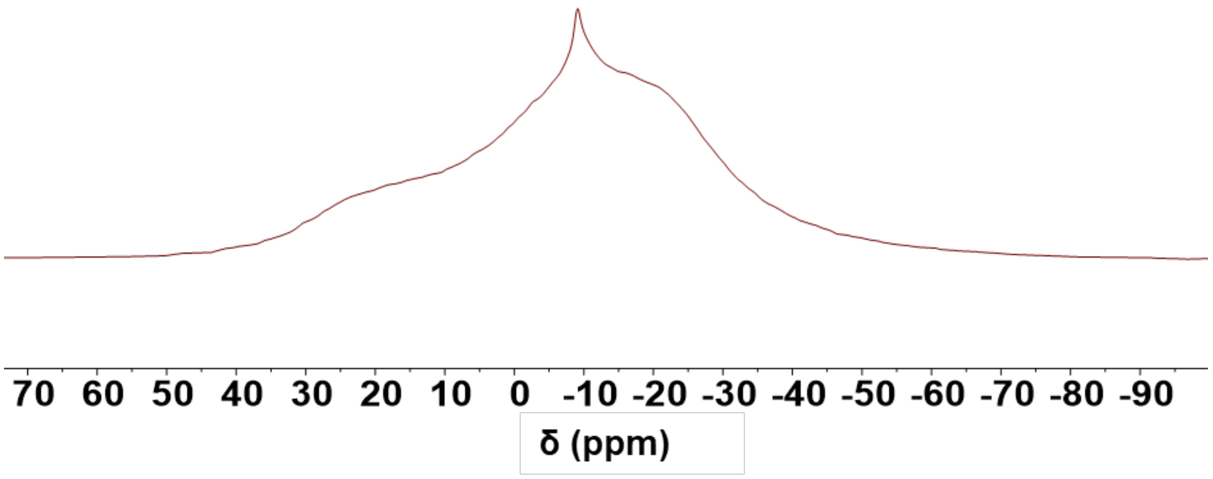

Figure S71. ^{11}B NMR spectrum of **6-^{i}Pr** (CD_2Cl_2, 128 MHz, 298 K). The $[BAr^F{}_4]^-$ signal at -9.1 ppm and the broad signal of the BH_2 (-6.3 to -14.1 ppm) are overlapping. The large broad peak from 50 to -50 ppm is the background boron resonance.

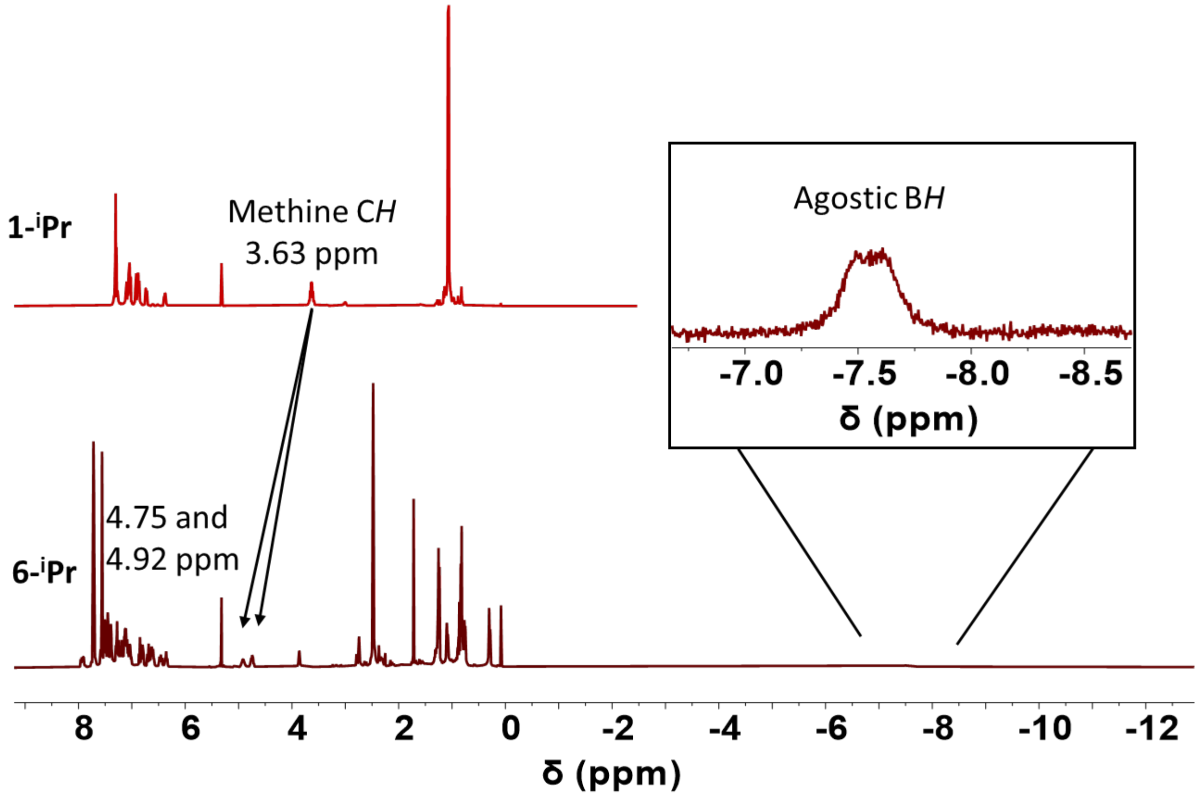

Figure S72. ^{1}H NMR of **1-^{i}Pr** (top) and **6-^{i}Pr** (bottom) illustrating the upfield shifted agostic B*H* and downfield shifted methine C*H* signals (500 MHz, CD_2Cl_2, 298 K).

Crystallographic and Refinement Data

Single crystal X-ray diffraction data for all samples were collected as follows: a typical crystal was mounted on a MiTeGen Micromounts using perfluoropolyether oil and cooled rapidly to the collection temperature in a stream of nitrogen gas using an Oxford Cryosystems Cryostream unit.[12] The structures of **2-^{i}Pr**, **2-Me**, **2-OMe**, **5-^{i}Pr** and **6-^{i}Pr** were collected at the Oxford Chemical Crystallography Service from the University of Oxford, with an Agilent SuperNova diffractometer (Cu Kαradiation, λ = 1.54180 Å). **1-^{i}Pr** and **2-H** were collected at the Department of Chemistry, University of York on an Oxford Diffraction SuperNova diffractometer using an EOS CCD camera.

Raw frame data were reduced using CrysAlisPro.[13] The structures were solved using SHELXT[14] and refined using full-matrix least squares refinement on all F2 data using the ShelXL-20XX[15] using the interface OLEX2.[16] All hydrogen atoms were placed in calculated positions (riding model). Disorder of the -CF_3 groups was treated by introducing a split site model and restraining geometries and displacement parameters.

Tables of data

	1-^{i}Pr	2-H	2-Me	2-OMe
Chemical formula	$C_{48}H_{52}OP_2$	$C_{83}H_{62}BF_{25}OP_2Rh$	$C_{79}H_{56}BF_{24}OP_2Rh$	$C_{79}H_{56}BF_{24}O_5P_2Rh$
Formula weight	706.83	1725.98	1652.89	1716.89
Temperature (K)	110	110	150	150
Crystal system	triclinic	triclinic	triclinic	triclinic
Space group	P-1	P-1	P-1	P-1
a (Å)	8.3692(5)	13.4274(2)	13.1702(2)	12.9929(4)
b (Å)	13.6847(9)	13.6083(2)	15.7363(3)	16.2926(6)
c (Å)	18.8298(8)	21.8679(3)	17.8817(3)	17.9735(6)
α (deg)	105.445(5)	106.3620(10)	81.607(2)	82.461(3)
β (deg)	99.637(4)	96.9450(10)	84.6750(10)	84.317(3)
γ (deg)	99.462(5)	91.2350(10)	84.5080(10)	84.684(3)
Volume (Å^3)	1999.2(2)	3799.34(10)	3637.75(11)	3741.1(2)
Z	2	2	2	2
ρ_{calc} g/cm^3	1.174	1.509	1.509	1.524
μ/mm^{-1}	1.242	3.177	3.271	3.246
Reflection collected	11012	76451	68981	34319
Independent reflections	6053	14513	15179	15530
Restraints / parameters	0 / 468	406 / 1193	690 / 1111	1441 / 1304
R_{int}	0.0531	0.0403	0.0457	0.0252
R_1 [I > 2σ(I)]	0.0537	0.0428	0.0421	0.0409
wR_2 [all data]	0.1449	0.1162	0.1146	0.1115
GooF	1.006	1.037	1.055	1.032
Residual electron density (e Å^{-3})	0.30 / -0.28	1.31 / -0.88	1.23 / -0.71	0.72 / -0.49
CCDC no.	2064124	2064125	2064127	2064128

	2-^{i}Pr	5-^{i}Pr	6-^{i}Pr
Chemical formula	$C_{87}H_{72}BF_{24}OP_2Rh$	$C_{81}H_{64}BF_{24}O_2P_2Rh$	$C_{83}H_{72}B_2F_{24}NOP_2Rh$
Formula weight	1765.10	1700.98	1741.88
Temperature (K)	150	150	150
Crystal system	monoclinic	monoclinic	triclinic
Space group	$P2_1/c$	$P2_1/n$	P-1
a (Å)	20.43890(10)	20.5769(2)	14.2087(6)
b (Å)	18.04470(10)	16.50650(10)	17.3935(5)
c (Å)	24.4986(2)	24.0640(2)	18.5698(6)
α (deg)	90	90	114.334(3)
β (deg)	113.5620(10)	110.1080(10)	98.207(3)
γ (deg)	90	90	103.273(3)
Volume (Å^3)	8282.12(11)	7675.20(12)	3921.4(3)
Z	4	4	2
ρ_{calc} g/cm^3	1.416	1.472	1.475
μ/mm^{-1}	2.909	3.126	3.064
Reflection collected	155774	93147	37813
Independent reflections	17246	15989	16262
Restraints / parameters	148 / 1175	0 / 1036	84 / 1087
R_{int}	0.0500	0.0430	0.0373
R_1 [I > 2σ(I)]	0.0306	0.0438	0.0510
wR_2 [all data]	0.0801	0.1242	0.1440
GooF	1.021	1.030	1.016
Residual electron density (e Å^{-3})	0.71 / -0.33	1.24 / -0.57	0.93 / -0.39
CCDC no.	2064126	2064129	2064130

Additional Comments on Crystal Structures and Refinement Data

1-^{i}Pr

The crystals were grown several times in different conditions and were of consistently poor quality as a result no diffraction was observed beyond 0.88 Å and hence the diffraction was only collected to this resolution. The data collected solves well showing the connectivity of the ligand with no disorder and a satisfactory R factor of 5.4%.

PROBLEM: The value of sine(theta_max)/wavelength is less than 0.575
RESPONSE: Diffraction only seen to 0.88 \%A resolution see
_publ_section_exptl_refinemen

2-H

The 1,2-$C_6H_4F_2$ is has rotational disorder and is additionally disordered across a symmetry element, this was modelled using fragmentDB with occupancies of each component fixed to 0.25.

2-OMe

Positional disorder was seen on two of the phenyl rings and three of the OMe groups, these were modelled with two components, RIGU and SADI restraints were applied to conserve sensible geometry.

2-^{i}Pr

Disordered solvent was also observed in the difference Fourier map, this appeared likely to be disordered pentane. However, a satisfactory refinement of the disordered solvent was not found and hence a solvent mask was applied.

6-^{i}Pr

The rhodium centre was modelled as disordered over two positions (ca. 95:5) with the minor rhodium component displaying an unusual binding mode to the ligand. It could not be determined whether this was a genuine isomer or as a consequence of absorption by the rhodium centre, consequently, this component was not considered further in the analysis. For atoms bound to the metal centre, B1 (H1a and H1b) and C39 (H39), the attached hydrogen atoms were located in the difference Fourier map and freely refined. All other hydrogen atoms were placed geometrically.

References

Experimental

1. E. W. Abel, M. A. Bennet, G. Wilkinson, *J. Chem. Soc. (Resumed)*, 1959, 3178-3182.
2. A. T. Lubben, J. S. McIndoe, A. S. Weller, *Organometallics*, 2008, **27**, 3303-3306.
3. Y. Zhu, V. H. Rawal, *J. Am. Chem. Soc.*, 2012, **134(1)**, 111-114.
4. J. Wang, G. Meng, K. Xie, L. Li, H. Sun, Z. Huang, *ACS Catalysis*, 2017, **7**, 7421-7430.
5. G. M. Moxham, H. Randell-Sly, S. K. Brayshaw, A. S. Weller, M. C. Willis, *Angew. Chem. Int. Ed.*, 2006, **45**, 7618-7622.
6. H. J. Hogben, M. Kryzstyniak, G. T. P. Charnock, P. J. Hore, I. Kuprov, *J. Magn. Reson.*, 2011, **208(2)**, 179-194.
7. G. L. Moxham, H. Randell-Sly, S. K Brayshaw, A. S. Weller, M. C. Willis, Chem. Eur. J., 2008, **14**, 8383-8397.
8. R. J. Pawley, G. L. Moxham, R. Dallanegra, A. B. Chaplin, S. K. Brayshaw, A. S. Weller, M. C. Willis, *Organometallics*, 2010, **29**, 1717-1728.
9. Python Software Foundation. Python Language Reference, version 3.7.1 Available at http://www.python.org.
10. Anaconda Software Distribution. Computer software. Version 2-2.4.0. Anaconda, November 2016. Available at https://anaconda.com.
11. E. Jones, E. Oliphant, P. Peterson, SciPy: Open Source Scientific Tools for Python, 2001. Available at http://www.scipy.org/ [Online; accessed 2019-05-14].
12. B. J. Cosier, A. M. Glazer, *J. Appl. Crystallogr.*, 1986, **19(2)**, 105.
13. Oxford Diffraction Ltd.; 2011.
14. G. M. Sheldrick, *Acta Crystallogr. Sect. A Found. Crystallogr.*, 2008, **64(1)**, 112.
15. G. M. Sheldrick, G. M. *Acta Crystallogr. Sect. A Found. Crystallogr.*, 2015, **71(1)**, 3.
16. O. V. Dolomanov, L. J. Bourhis, R. J. Gildea, J. A. K. Howard, H. Puschmann, *J. Appl. Crystallogr.*, 2009, **42(2)**, 339.

Computational

1. Gaussian 16, Revision A.03,
M. J. Frisch, G. W. Trucks, H. B. Schlegel, G. E. Scuseria, M. A. Robb, J. R. Cheeseman, G. Scalmani, V. Barone, G. A. Petersson, H. Nakatsuji, X. Li, M. Caricato, A. V. Marenich, J. Bloino, B. G. Janesko, R. Gomperts, B. Mennucci, H. P. Hratchian, J. V. Ortiz, A. F. Izmaylov, J. L. Sonnenberg, D. Williams-Young, F. Ding, F. Lipparini, F. Egidi, J. Goings, B. Peng, A. Petrone, T. Henderson, D. Ranasinghe, V. G. Zakrzewski, J. Gao, N. Rega, G. Zheng, W. Liang, M. Hada, M. Ehara, K. Toyota, R. Fukuda, J. Hasegawa, M. Ishida, T. Nakajima, Y. Honda, O. Kitao, H. Nakai, T. Vreven, K. Throssell, J. A. Montgomery, Jr., J. E. Peralta, F. Ogliaro, M. J. Bearpark, J. J. Heyd, E. N. Brothers, K. N. Kudin, V. N. Staroverov, T. A. Keith, R. Kobayashi, J. Normand, K. Raghavachari, A. P. Rendell, J. C. Burant, S. S. Iyengar, J. Tomasi, M. Cossi, J. M. Millam, M. Klene, C. Adamo, R. Cammi, J. W. Ochterski, R. L. Martin, K. Morokuma, O. Farkas,
J. B. Foresman, and D. J. Fox, Gaussian, Inc., Wallingford CT, 2016.
2. A. D. Becke, Phys. Rev. A, 1988, **38**, 3098
3. J. P. Perdew, Phys. Rev. B, 1986, **33**, 8822
4. D. Andrae, U. Haussermann, M. Dolg, H. Stoll, Preuss, Theor. Chim. Acta 1990, **77**, 123
5. A. Hollwarth, M. Bohme, S. Dapprich, A. W. Ehlers, A. Gobbi, V. Jonas, K. F. Kohler, R. Stegmann, A. Veldkamp, G. Frenking, Chem. Phys. Lett. 1993, **208**, 237
6. W. J. Hehre, R. Ditchfield, J. A. Pople, Chem. Phys. 1972, **56**, 2257
7. P. C. Hariharan, J. A. Pople, Theor. Chim. Acta **1973**, 28, 213
8. S. Grimme, S. Ehrlich and L. Goerigk, J. Comp. Chem., 2011, **32**, 1456-1465
9. G. Scalmani and M. J. Frisch, J. Chem. Phys., 2010, **132**, 114110
10. R. F. W. Bader, Atoms in Molecules: A Quantum Theory; Oxford University Press, 1990
11. AIMAll (Version 17.11.14), Todd A. Keith, TK Gristmill Software, Overland Park KS, USA, 2017 (aim.tkgristmill.com)
12. NBO 6.0. E. D. Glendening, J. K. Badenhoop, A. E. Reed, J. E. Carpenter, J. A. Bohmann, C. M. Morales, C. R. Landis, and F. Weinhold (Theoretical Chemistry Institute, University of Wisconsin, Madison, WI, 2013); http://nbo6.chem.wisc.edu/
13. E. R. Johnson, S. Keinan, P. Mori-Sanchez, J. Contreras-Garcia, A. J. Cohen, and W. Yang, J. Am. Chem. Soc. 2010, **132**, 6498-6506
14. J. Contreras-Garcia, E. R. Johnson, S. Keinan, R. Chaudret, J-P. Piquemal, D. N. Beratan, and W. Yang. J. Chem. Theory Comput. 2011, **7**, 625-632
15. Chemcraft - graphical software for visualization of quantum chemistry computations. https://www.chemcraftprog.com
16. P. Ren, J. W. J. Ponder, Phys. Chem. B 2003, **107**, 5933
17. P. Ren, J. W. J Ponder, Comput. Chem. 2002, **23**, 1497
18. Pappu, R. V.; Hart, R. K.; Ponder, J. W. J. Phys. Chem. B 1998, **102**, 9725
19. L. J. L. Häller, M. J. page, S. Erhardt, S. A. Macgregor, M. F. Mahon, M. Abu Naser, A. Vélez and M. K. Whittlesey, J. Am. Chem. Soc. 2010, 132, **51**, 18408
20. G. te Velde, F. M. Bickelhaupt, E. J. Baerends, C. Fonseca Guerra, S. J. A. van Gisbergen, J. G. Snijders and T. Ziegler, *J. Comp. Chem.* 2001, **22**, 931-967.
21. E.J. Baerends, T. Z., A.J. Atkins, J. Autschbach, O. Baseggio, D. Bashford, A. Bérces, F.M. Bickelhaupt, C. Bo, P.M. Boerrigter, L. Cavallo, C. Daul, D.P. Chong, D.V. Chulhai, L. Deng, R.M. Dickson, J.M. Dieterich, D.E. Ellis, M. van Faassen, L. Fan, T.H. Fischer, A. Förster, C. Fonseca Guerra, M. Franchini, A. Ghysels, A. Giammona, S.J.A. van Gisbergen, A. Goez, A.W. Götz, J.A. Groeneveld, O.V. Gritsenko, M. Grüning, S. Gusarov, F.E. Harris, P. van den Hoek, Z. Hu, C.R. Jacob, H. Jacobsen, L. Jensen, L. Joubert, J.W. Kaminski, G. van Kessel, C. König, F. Kootstra, A. Kovalenko, M.V. Krykunov, E. van Lenthe, D.A. McCormack, A. Michalak, M. Mitoraj, S.M. Morton, J.

Neugebauer, V.P. Nicu, L. Noodleman, V.P. Osinga, S. Patchkovskii, M. Pavanello, C.A. Peeples, P.H.T. Philipsen, D. Post, C.C. Pye, H. Ramanantoanina, P. Ramos, W. Ravenek, J.I. Rodríguez, P. Ros, R. Rüger, P.R.T. Schipper, D. Schlüns, H. van Schoot, G. Schreckenbach, J.S. Seldenthuis, M. Seth, J.G. Snijders, M. Solà, M. Stener, M. Swart, D. Swerhone, V. Tognetti, G. te Velde, P. Vernooijs, L. Versluis, L. Visscher, O. Visser, F. Wang, T.A. Wesolowski, E.M. van Wezenbeek, G. Wiesenekker, S.K. Wolff, T.K. Woo, A.L. Yakovlev *ADF 2019.3*, SCM, Theoretical Chemistry, Vrije Universiteit, Amsterdam, The Netherlands, 2019.

22. A. D. Becke, *J. Chem. Phys.* 1993, **98**, 5648-5652.
23. E. Van Lenthe and E. J. Baerends, *J. Comp. Chem.* 2003, **24**, 1142-1156.
24. E. van Lenthe, A. Ehlers and E.-J. Baerends, *J. Chem. Phys.* 1999, **110**, 8943-8953
